# Multi-ancestry sequencing analysis in 293,141 participants identifies predisposition DNA-repair genes associated with HCC risk

**DOI:** 10.1016/j.jhepr.2026.102019

**Published:** 2026-08-29

**Authors:** Alexis M. Garofalo, Perapa Chotiprasidhi, Josephine Princy Johnson, Karina Sato-Espinoza, Jun Ma, Hunter Miller, Daniel O'Brien, Lindsay Guare, Katie M. Cardone, Nicole Palmiero, Zachary Rodriguez, David E. Kaplan, Julie A. Lynch, Philip S. Tsao, Daniel J. Rader, Lewis R. Roberts, Jose D. Debes, Emuobor Odeghe, Olufunmilayo Lesi, Ganiyat Oyeleke, Ângelo Z. Mattos, Marco Arrese, Enrique Carrera, Jhon Prieto, Andre Boonstra, Javier Diaz-Ferrer, Edith N. Okeke, Jun Wang, Lifang Hou, Adwoa Agyei-Nkansah, Mary Yeboah Afihene, Yaw Asante Awuku, Albert Nyanga, Kyong-Mi Chang, Samuel O. Antwi, Marijana Vujković, Anurag Verma, Kirk J. Wangensteen

**Affiliations:** 1 Department of Medicine, Division of Translational Medicine and Human Genetics, University of Pennsylvania Perelman School of Medicine, Philadelphia, PA, USA; 2 Department of Medicine, Division of Gastroenterology and Hepatology, Mayo Clinic, Rochester, MN, USA; 3 Corporal Michael J. Crescenz VA Medical Center, Philadelphia, PA, USA; 4 Department of Pathology, University of Pennsylvania Perelman School of Medicine, Philadelphia, PA, USA; 5 Department of Genetics, University of Pennsylvania Perelman School of Medicine, Philadelphia, PA, USA; 6 Department of Medicine, Division of Gastroenterology and Hepatology, University of Pennsylvania Perelman School of Medicine, Philadelphia, PA, USA; 7 VA Informatics and Computing Infrastructure, VA Salt Lake City Health Care System, Salt Lake City, UT, USA; 8 Department of Internal Medicine, Division of Epidemiology, University of Utah School of Medicine, Salt Lake City, UT, USA; 9 VA Palo Alto Healthcare System, Palo Alto, CA, USA; 10 Department of Medicine, Stanford University School of Medicine, Stanford, CA, USA; 11 Department of Medicine, University of Minnesota, Minneapolis, MN, USA; 12 University of Lagos, Lagos, Nigeria; 13 Graduate Program in Medicine, Division of Hepatology, Federal University of Health Sciences of Porto Alegre, Porto Alegre, Brazil; 14 Departamento de Gastroenterología, Escuela de Medicina, Pontificia Universidad Católica de Chile, Santiago, Chile; 15 Department of Gastroenterology and Hepatology, Hospital Especialidades Eugenio Espejo, Quito, Ecuador; 16 School of Medicine, Universidad San Francisco de Quito, Quito, Ecuador; 17 Centro de Enfermedades Hepáticas y Digestivas, Bogotá, Colombia; 18 Department of Gastroenterology and Hepatology, Erasmus University Medical Center, Rotterdam, The Netherlands; 19 Department of Digestive Diseases, Division of Hepatology, Hospital Nacional Edgardo Rebagliati Martins, Lima, Peru; 20 Jos University Teaching Hospital, Jos, Nigeria; 21 Department of Preventive Medicine, Division of Cancer Epidemiology and Prevention, Northwestern University, Chicago, IL, USA; 22 Korle Bu Teaching Hospital, Accra, Ghana; 23 Kwame Nkrumah University of Science and Technology, Kumasi, Ghana; 24 University of Health and Allied Sciences, Ho, Ghana; 25 Mbingo Baptist Hospital, Mbingo, Cameroon; 26 Department of Quantitative Health Sciences, Division of Epidemiology, Mayo Clinic, Jacksonville, FL, USA; 27 Department of Epidemiology, University of Pennsylvania Perelman School of Medicine, Philadelphia, PA, USA

**Keywords:** Liver cancer, Rare variants, Clinical genetics, Genetic association study, Biobanks, Electronic health records, Precision medicine, Cancer genetics, Lynch syndrome, *BRCA2*, *MSH6*

## Abstract

**Background & Aims:**

Genetic testing for Lynch and *BRCA1/2*-associated hereditary cancer syndromes is recommended in patients with colon or pancreatic cancer, but their association with hepatocellular carcinoma (HCC) risk is unknown. We evaluated associations between rare germline variants in DNA-repair genes and HCC risk across ancestrally diverse cohorts.

**Methods:**

We analyzed whole exome and whole genome sequencing data from 2,594 HCC cases and 290,547 cancer-free controls from diverse biobanks and cohorts: Penn Medicine BioBank, All of Us, Mayo Clinic, ESCALON, and the Million Veteran Program. Participants were classified into six population groups. We focused on six DNA-repair genes previously implicated in HCC: *BRCA2*, *BRIP1*, *MSH6*, *PMS2*, *CHEK2*, and *FANCA*. Gene-level burden analyses of rare predicted loss-of-function (pLoF) and damaging missense variants were performed in European and African populations, and across all six ancestry groups.

**Results:**

In the European population, *MSH6*, a Lynch syndrome-associated gene, had the strongest association with HCC, with a 2.75-fold increased HCC risk at 1% minor allele frequency (MAF) (odds ratio [OR] = 2.75 [1.50, 5.04], *p* = 0.001, false discovery rate [FDR] q = 0.02), while *PMS2*, showed a nominally significant association at the same MAF threshold (OR = 1.90 [1.08, 3.34], *p* = 0.03, FDR q = 0.09). Combined analysis across all populations strengthened the *MSH6* finding (OR = 2.53 [1.43, 4.49], *p* = 0.001, FDR q = 0.01) at an MAF of 0.1%. A significant *BRCA2* association was also observed in the combined analysis at an MAF of 0.1% (OR = 2.26 [1.39, 3.67], *p* = 0.001, FDR q = 0.01).

**Conclusions:**

Rare variants in *MSH6* and *BRCA2* are significantly associated with increased HCC risk, revealing a previously unconfirmed role for DNA-repair genes in HCC susceptibility across ancestrally diverse populations. These findings may inform genetic risk stratification and surveillance strategies.

**Impact and implications:**

Rare variants in *MSH6* and *BRCA2* genes are associated with 2.3 to 2.8-fold higher HCC risk. These findings may warrant further evaluation of liver cancer risk in individuals with *MSH6*-associated Lynch syndrome or *BRCA2*-associated hereditary cancer syndromes.

## Highlights

• Rare variants in *MSH6* and *BRCA2* increase hepatocellular carcinoma risk two-to threefold.
• *MSH6* and *BRCA2* variants linked to liver cancer risk across diverse ancestries.
• *BRCA2* liver cancer risk persists after adjusting for liver disease comorbidities.
• *BRCA2* risk is stronger in females; *MSH6* association is driven primarily by males.

## Graphical abstract

**Figure:**
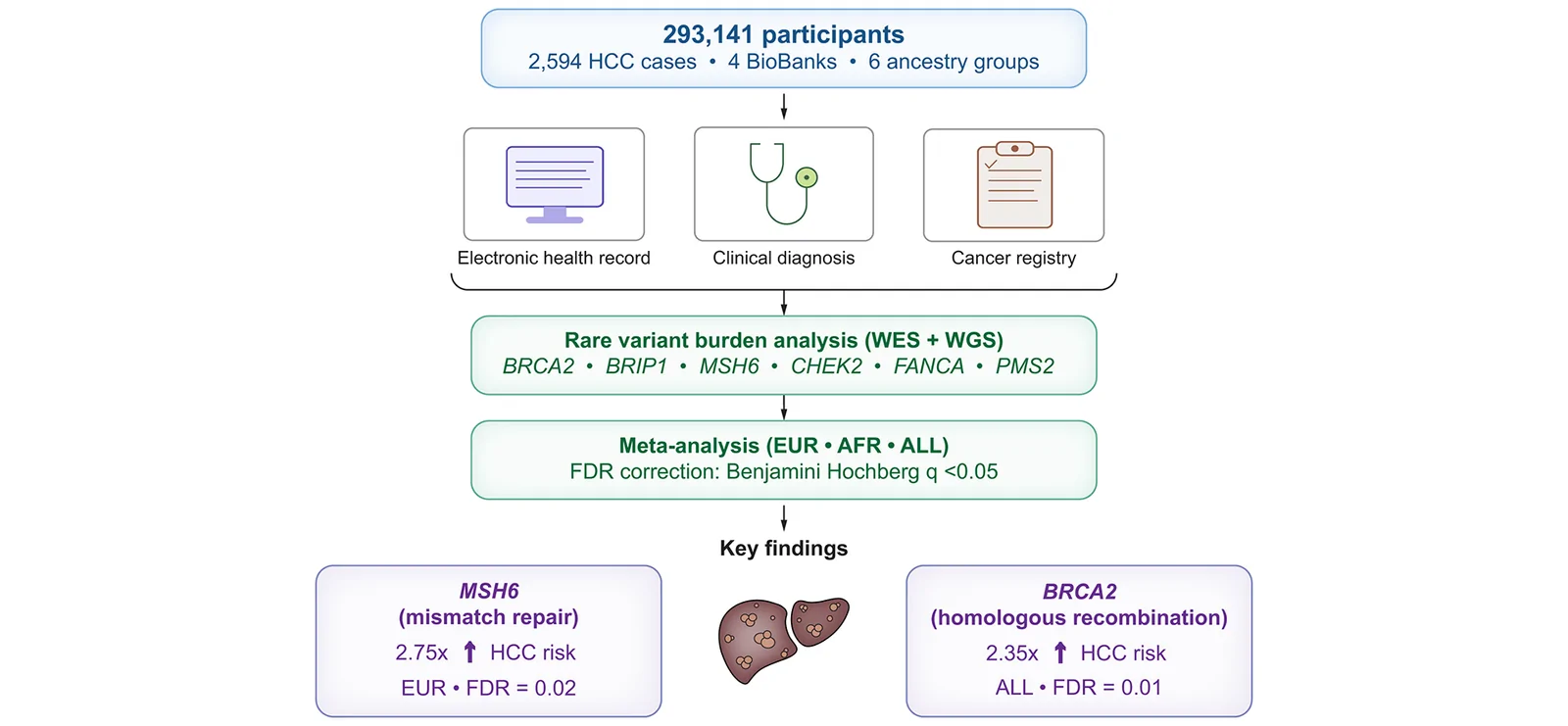

## Introduction

Hepatocellular carcinoma (HCC) is linked to environmental risk factors, but a family history of liver cancer increases HCC risk two-to fourfold, regardless of those factors, suggesting a strong hereditary component.^1^^,^^2^ Genome-wide association studies (GWAS) have identified about a dozen common variants associated with HCC.^3^^,^^4^ While common variants explain a modest proportion of HCC heritability (10–30%), rare variants with larger effect sizes may account for the remainder.^5^ Recent large-scale studies in Asian biobank populations reported a nominal *BRCA2* liver cancer association and found no clear association for *MSH6*.^6^^,^^7^ We conducted a case-control association analysis of rare variants in six DNA-repair genes previously identified in HCC cohort studies,^8^^,^^9^ using diverse biobanks with sequencing and electronic health record data.

## Materials and methods

### Study design and participants

This study included participants from several diverse biobanks: Penn Medicine BioBank (PMBB), All of Us Research Program (AOU), Mayo Clinic, and Million Veteran Program (MVP).[10], [11], [12] At the Mayo Clinic the samples were collected from internal and external biobanks and cohort studies including ESCALON^13^ (Table S1). HCC cases were identified using standardized algorithms based on International Classification of Diseases (ICD)-9/10 diagnosis codes (PMBB, AOU), AASLD guidelines (Mayo Clinic),^14^ and cancer registry data (MVP). Cancer-free controls were individuals without cancer diagnosis codes (MVP, PMBB, AOU) or cancer diagnosis (Mayo Clinic). Specific diagnostic codes, inclusion/exclusion criteria, and baseline liver disease characteristics are provided (Table S2 and S12).

Participants were classified into six population groups based on genetic similarity to the 1000 Genomes Project and, for AOU, additionally the Human Genome Diversity Project: European (EUR), African (AFR), Admixed American (AMR), East Asian (EAS), South Asian (SAS), and Middle Eastern (MID) (Fig. S6). We performed separate analyses for EUR and AFR, and a combined multi-population analysis (ALL) that includes all six population groups.

### Genomic sequencing and quality control

Genomic DNA extracted from participants’ blood or saliva samples were used for whole exome sequencing (WES) or whole exome sequencing (WGS). Detailed information on the sequencing protocols, variant calling workflows and quality control metrics for each biobank is provided in the Supplementary Methods.

### Variant annotation and classification

We developed a standardized workflow for consistent variant annotation across biobanks. Variants were functionally annotated using Ensembl Variant Effect Predictor (VEP) and filtered to rare variants (minor allele frequency [MAF] ≤1% or missing in the Genome Aggregation Database [gnomAD]) with matched annotation from NCBI and EBI (MANE) transcripts.[15], [16], [17] We classified variants into three functional categories: predicted loss-of-function (pLoF), damaging missense, and combined pLoF + damaging missense variants. The pLoF tier was the highest level of confidence of pathogenic/likely pathogenic status according to ClinVar and/or predicted impact (Table S3–S5). A complete list of rare variants included in the six-gene-burden analysis across all four biobanks is provided (Table S6).

### Statistical analysis

For each biobank, we performed gene-burden association tests using the Scalable and Accurate Implementation of Generalized mixed model (SAIGE-GENE+) for EUR, AFR, and ALL populations.^18^ Models were adjusted for age, sex, and genetic ancestry principal components. We tested three maximum MAF thresholds (1%, 0.1%, and 0.01%) to evaluate the contribution of rare to ultra-rare variants.

Next, we conducted fixed-effects inverse variance weighted meta-analysis for EUR and AFR populations, with random-effects inverse variance weighted meta-analysis for ALL populations using the metafor R package (R Foundation for Statistical Computing, Vienna, Austria). Statistical significance was determined by false discovery rate (FDR) correction (Benjamini–Hochberg, q <0.05) across all genes, variant bins, and MAF thresholds.

## Results

### Study population and variant distribution

We analyzed genetic sequencing data on 293,141 participants across several diverse biobanks, including 2,594 HCC cases and 290,547 cancer-free controls. HCC cases were predominantly male and older than controls (Table 1).

**Table 1:** Baseline characteristics of hepatocellular carcinoma cases and cancer-free controls by study site.

Metric	PMBB	AOU	Mayo	MVP
Demographics, % male				
HCC cases	354 (72.10)	483 (54.58)	459 (71.50)	569 (98.78)
Cancer-free controls	13,422 (47.23)	76,895 (37.01)	1,903 (65.15)	47,735 (92.79)
Demographics, % female				
HCC cases	137 (27.90)	402 (45.42)	183 (28.50)	7 (1.22)
Cancer-free controls	14,999 (52.77)	130,866 (62.99)	1,018 (34.85)	3,709 (7.21)
Age				
HCC cases	60.44 ± 10.99	63.30 ± 10.60	59.2 ±13.63	63.5 ± 6.8
Cancer-free controls	50.35 ± 16.99	49.50 ± 16.64	62.04 ± 12.83	62.5 ± 13.9
Total N (case | control)				
HCC cases | cancer-free controls	491 | 28,421	885 | 207,761	642 | 2,921	576 | 51,444
Ancestry subgroups (case | control)				
EUR: HCC cases | cancer-free controls	352 | 19,002	515 | 111,262	314 | 2,162	326 | 32,406
AFR: HCC cases | cancer-free controls	121 | 7,518	166 | 45,261	192 | 468	191 | 14,316
AMR: HCC cases | cancer-free controls	5 | 457	177 | 43,040	120 | 214	54 | 3,938
EAS: HCC cases | cancer-free controls	10 | 582	15 | 4,539	0 | 0	4 | 683
SAS: HCC cases | cancer-free controls	0 | 524	7 | 2,825	0 | 0	1 | 33
MID: HCC cases | cancer-free controls	0 | 0	5 | 834	0 | 0	0 | 68
Total carrier counts case + control (npLOF | ndamag | npLOF + damaging)^∗^				
BRCA2	287 | 93 | 380	934 | 538 | 1,469	9 | 0 | 9	277 | 120 | 397
FANCA	116 | 40 | 156	771 | 286 | 1,055	20 | 36 | 56	437 | 74 | 511
MSH6	180 | 10 | 190	233 | 44 | 277	5 | 18 | 23	82 | 299 | 381
BRIP1	56 | 95 | 151	398 | 802 | 1,200	6 | 9 | 14	93 | 96 | 189
CHEK2	328 | 99 | 427	2,077 | 505 | 2,582	44 | 8 | 52	539 | 33 | 572
PMS2	283 | 218 | 501	1,831 | 1,513 | 3,344	11 | 36 | 45	938 | 230 | 1,168

Baseline characteristics of study participants across biobanks. Data are presented as n (%) for categorical variables and mean ± standard deviation for continuous variables. ^∗^Carrier counts are shown for gene annotations at an MAF of 0.01 only. Carrier counts presented as npLOF | ndamaging | npLOF + damaging for each gene. AFR, African; AMR, Admixed American; EAS, East Asian; EUR, European; HCC, hepatocellular carcinoma; MAF, minor allele frequency; MID, Middle Eastern; SAS, South Asian.

Variant carriers were identified for each gene in all biobanks, with counts varying by gene and biobank (Table 1; Fig. S1 and Table S10 and S11).

### Rare variants in *MSH6, PMS2,* and *BRCA2* are associated with higher HCC risk

Gene-burden meta-analyses in the EUR population showed a 2.75-fold increased risk of HCC in the pLoF + damaging missense burden analysis for *MSH6*, at 1% MAF (odds ratio [OR] = 2.75 [1.50, 5.04], *p* = 0.004, FDR q = 0.02) (Fig. 1A and Table S7). We observed similar findings in the ALL group at 1% MAF (OR = 2.39 [1.33, 4.29], *p* = 0.004, FDR q = 0.02) and 0.1% MAF (OR = 2.53 [1.43, 4.49], *p* = 0.001, FDR q = 0.01) (Fig. 1A and Table S9). We also observed a nominally significant association for the pLoF + damaging missense burden in *PMS2* in EUR populations at 1% MAF (OR = 1.90 [1.08, 3.34], *p* = 0.03, FDR q = 0.09) (Fig. 1B and Table S7). In the ALL group, *BRCA2* pLoF burden was significantly associated with increased risk of HCC at both 1% and 0.1% MAF thresholds (OR = 2.35 [1.39, 3.60], *p* = 0.001, FDR q = 0.01) and (OR = 2.26 [1.39, 3.67], *p* = 0.001, FDR q = 0.01) (Fig. 1C and Table S9). In the AFR population, a nominally significant association for the pLoF + damaging missense burden in *BRCA2* at 0.01% MAF was not significant after multiple testing correction (OR = 3.28 [0.99, 10.92], *p* = 0.05, FDR q = 0.26) (Fig. 1C and Table S8). A sensitivity analysis requiring ClinVar pathogenic/likely pathogenic annotation for both pLoF and damaging missense categories was not feasible for *MSH6* or *PMS2*, as too few HCC cases carried qualifying variants in these genes (Table S13 and Fig. S7).

**Fig. 1:**
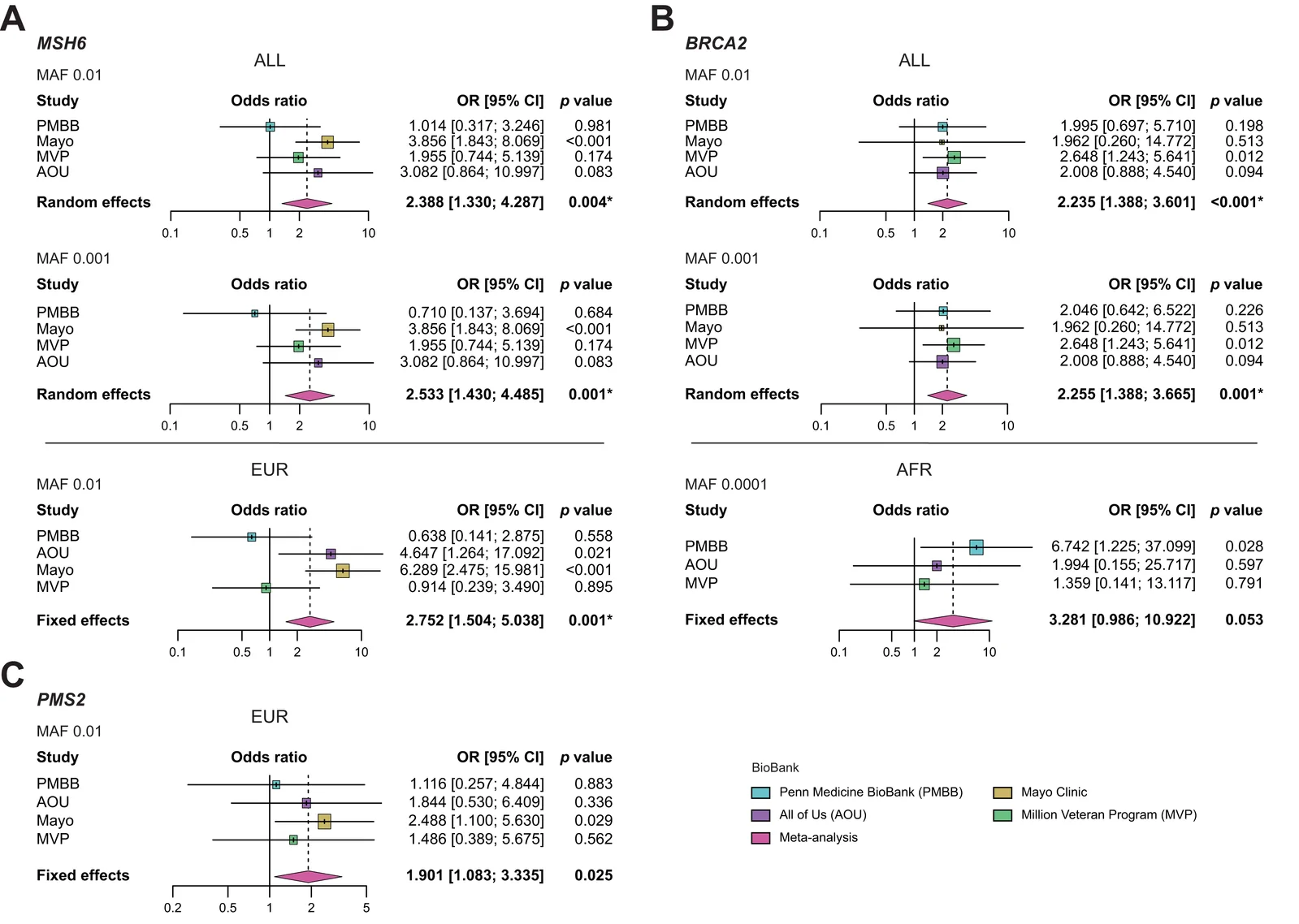
**Meta-analysis forest plots of six-gene-burden for HCC risk.** Forest plots of odds ratios and 95% confidence intervals for gene-burden associations with HCC risk for: (A) *MSH6*; (B) *PMS2*; and (C) *BRCA2*. Results stratified by population: ALL, EUR (European), and AFR (African) with MAF thresholds (0.01, 0.001, and 0.0001). BioBank results: PMBB, AOU, Mayo, and MVP. Box size = study weight; diamonds = pooled estimates. Asterisks (∗) indicate FDR <0.05 (Benjamini–Hochberg). The *p* values were from inverse variance weighted meta-analysis. Significant: *MSH6* EUR/ALL (*p* ≤0.004, FDR ≤0.02) and *BRCA2* ALL (*p* = 0.001, FDR = 0.01). Non-significant: *PMS2* EUR (*p* = 0.03, FDR = 0.09) and *BRCA2* AFR (*p* = 0.05, FDR = 0.26). ALL, all ancestry groups combined; AOU, All of Us Research Program; FDR, false discovery rate; HCC, hepatocellular carcinoma; MAF, minor allele frequency; Mayo, Mayo Clinic; MVP, Million Veteran Program.

### The rare variant association in *BRCA2* is independent of liver disease comorbidities

Carriers of *BRCA2* and *MSH6* rare variants were not enriched for specific liver disease comorbidities among HCC cases (Table S14 and Fig. S3). Comorbidity-adjusted burden analyses confirmed *BRCA2* pLoF remained significant in the ALL group at the 1% MAF threshold (OR = 2.37 [1.42, 3.97], *p* = 0.001, FDR q = 0.02), 0.1% MAF (OR = 2.35 [1.39, 3.98], *p* = 0.001, FDR q = 0.02), and 0.01% MAF (OR = 2.88 [1.59, 5.20], *p* = 4.56 × 10^-4^, FDR q = 0.02) (Table S15 and Fig. S2).

### Sex-stratified associations and co-occurring cancer enrichment in variant carriers

Sex-stratified burden analyses revealed significant associations for *BRCA2* pLoF + damaging missense in females (ALL_F: OR = 3.51 [1.63, 7.56], *p* = 0.001, FDR q = 0.05) and *MSH6* pLoF + damaging missense in males (ALL_M: OR = 2.75 [1.57, 4.82], *p* = 3.98 × 10^-4^, FDR q = 0.04) at MAF thresholds of 0.01% and 1%, respectively (Table S16 and Fig. S5). Age at HCC diagnosis did not differ significantly between carriers and non-carriers of *BRCA2* or *MSH6* variants across cohorts (Fig. S4). Among HCC cases, carriers of damaging missense variants showed significant enrichment for co-occurring cancers compared to non-carriers: *MSH6* carriers were enriched for breast cancer (OR = 22.6 [2.93, 174.7], *p* = 0.003, FDR q = 0.033) and ovarian cancer (OR = 24.5 [3.18, 189.8], *p* = 0.002, FDR q = 0.031), and *FANCA* carriers were enriched for prostate cancer (OR = 4.66 [1.61, 13.49], *p* = 0.005, FDR q = 0.046). *CHEK2* results were excluded because of insufficient carrier counts (Table S17).

## Discussion

Our study provides evidence that rare variants in *MSH6* and *BRCA2* are associated with increased HCC risk across ancestrally diverse populations, supporting a role for these genes in liver cancer susceptibility. These associations are biologically plausible given the roles of *MSH6* and *PMS2* in mismatch repair and *BRCA2* in homologous recombination, pathways whose disruption increases cancer susceptibility.

Analyses of Asian populations by other groups identified a nominal *BRCA2*-liver cancer signal that did not reach stringent significance thresholds and no *MSH6* association.^6^^,^^7^ In contrast, our analysis across diverse biobanks represents a comprehensive and ancestrally diverse investigation of rare germline HCC susceptibility.

The *BRCA2* pLoF association remained significant after adjusting for major liver disease comorbidities (HBV, HCV, alcohol-related liver disease, and metabolic dysfunction-associated steatohepatitis/metabolic dysfunction-associated steatotic liver disease), suggesting the risk does not depend on the type of environmental factor. Sex-stratified analyses revealed that the pLoF + damaging missense association for *BRCA2* was stronger in females and the *MSH6* association was driven primarily by males, highlighting possible sex-related heterogeneity in genetic susceptibility to HCC. Among HCC cases, *MSH6* damaging missense carriers showed enrichment for breast and ovarian cancers, consistent with known *MSH6* biology, while *BRCA2* carriers showed no such enrichment. These results should be interpreted with caution given the limited number of participants with variants in these genes. Together, these findings have potential clinical implications. While genetic testing is already recommended for patients with pancreatic and colon cancers,^19^^,^^20^ its role in HCC requires further study. Carrier status for variants in these genes may inform HCC surveillance strategies. While HCC risk in the general population is relatively low, underlying liver disease increases this risk by more than 40-fold.^21^ Lynch syndrome or *BRCA2* carrier status could further increase this risk. Whether Lynch syndrome or *BRCA2* carrier status should inform treatment selection with immune checkpoint inhibitors or poly ADP-ribose polymerase (PARP) inhibitors in HCC warrants further investigation.^22^^,^^23^ Patients with HCC who also have Lynch syndrome or *BRCA2* variants are at heightened risk for developing additional primary cancers in other organs, making comprehensive surveillance important.

Several limitations of our study should be acknowledged. First, variant carriers were not present for all six genes across all biobanks, limiting meta-analyses. Second, although we used a multi-tiered approach for high-confidence pLoF variant classification, only *BRCA2* achieved statistical significance for pLoF variants alone. *MSH6* and *PMS2* associations required inclusion of damaging missense variants, indicating lower confidence in the specific variant classes driving these associations. A ClinVar-restricted sensitivity analysis was not feasible because of insufficient case carriers, underscoring the necessity of computational predictions for rare variant burden analyses at biobank scale. Many rare variants from populations of African and Latin American ancestry may lack ClinVar classifications due to ancestral ascertainment bias in clinical variant databases.^24^^,^^25^ Third, broader analyses beyond the six genes tested here are needed to identify additional HCC susceptibility genes.

## Abbreviations

AFR, African; ALL, all ancestry groups combined; AMR, Admixed American; AOU, All of Us Research Program; EAS, East Asian; EUR, European; FDR, false discovery rate; gnomAD, Genome Aggregation Database; GWAS, genome-wide association study; HCC, hepatocellular carcinoma; MAF, minor allele frequency; MANE, Matched Annotation from NCBI and EMBL-EBI; MID, Middle Eastern; MVP, Million Veteran Program; pLoF, predicted loss-of-function; PMBB, Penn Medicine BioBank; SAIGE-GENE+, Scalable and Accurate Implementation of Generalized mixed model; SAS, South Asian; WES, whole exome sequencing; WGS, whole genome sequencing.

## Data availability

Data supporting the findings of this study are derived from four biobank resources, none of which are publicly available because of participant privacy and confidentiality protections. All of Us Research Program Controlled Tier Dataset v8 data are accessible to authorized researchers through the All of Us Researcher Workbench (https://allofus.nih.gov/article/opportunities-researchers). Million Veteran Program (MVP) data access is restricted to VA investigators on approved federally funded projects; requests can be made to the MVP Program Office. Mayo Clinic Biobank data are available to authorized researchers in collaboration with a Mayo Clinic investigator; requests can be directed to Biobank@mayo.edu. Penn Medicine BioBank (PMBB) data may be available upon reasonable request and with PMBB permission. Analysis code is deposited in the GitHub repository: https://github.com/Verma-Lab/hcc-rare-variant-analysis.

## Financial support

10.13039/100000002NIH R37CA259201, 10.13039/100000002NIH R37CA297814, 10.13039/100000002NIH K01CA237875, 10.13039/100000871Mayo Clinic Office of Research Diversity and Inclusion, 10.13039/100000054National Cancer Institute (10.13039/100000054NCI) through the 10.13039/100000871Mayo Clinic Hepatobiliary SPORE (P50 CA210964), 10.13039/100000054National Cancer Institute. Grant Number: 10.13039/100000002NIH/10.13039/100000054NCI U54CA221205, 10.13039/501100004359Swedish Research Council, and International Collaboration Grant ID: dnr 2015–05928.

## **Authors’ contributions**

Conceptualization: AV KJW; Methodology: AMG, PC, JPJ, KS, JM, HM, DO, AV, KJW; Investigation: AMG, PC, JPJ, KSE, JM, HM, DO, LG, KMC, NP, ZR; Visualization: AMG, AV ; Funding acquisition: KJW, AV,JD,LR,KMC; Project administration: KJW, AV; Supervision: KJW, AV, MV; Writing – original draft: AMG, PC, JPJ, JM, AV, KJW; Writing – review & editing: HM, DEK, JAL, PST, DJR, LRR, JDD, EO, OL, GO, AZM, MA, EC, JP, AB, JDF, ENO, JW, LH, AAN, MYA, YAA, AN, KMC, SOA

## Conflicts of interest

JAL reports grants from Alnylam Pharmaceuticals, Inc., AstraZeneca Pharmaceuticals LP, Biodesix, Inc, Janssen Pharmaceuticals, Inc., Novartis International AG, Parexel International Corporation through the University of Utah or Western Institute for Veteran Research outside the submitted work. The remaining authors declare no conflicts of interest that pertain to this work.

Please refer to the accompanying ICMJE disclosure forms for further details.
