# Supplementary material for: Multi-ancestry sequencing analysis in 293,141 participants identifies predisposition DNA-repair genes associated with HCC risk
Source: JHEP Rep. 2026 Aug 29;8(12):102019. doi: 10.1016/j.jhepr.2026.102019 (PMC13755941; doi:10.1016/j.jhepr.2026.102019)
Supplement: Supplementary file 1 [file mmc1.pdf]

Multi-ancestry sequencing analysis in 293,141 participants identifies predisposition DNA repair genes associated with HCC risk

Alexis M. Garofalo, Perapa Chotiprasidhi, Josephine Princy Johnson, Karina Sato-Espinoza, Jun Ma, Hunter Miller, Daniel O'Brien, Lindsay Guare, Katie M. Cardone, Nicole Palmiero, Zachary Rodriguez, David E. Kaplan, Julie A. Lynch, Philip S. Tsao, Daniel J. Rader, Lewis R. Roberts, Jose D. Debes, Emuobor Odeghe, Funmilayo Lesi, Ganiyat Oyeleke, Ângelo Z. Mattos, Marco Arrese, Enrique Carrera, Jhon Prieto, Andre Boonstra, Javier Diaz-Ferrer, Edith N. Okeke, Jun Wang, Lifang Hou, Adwoa Agyei-Nkansah, Mary Yeboah Afihene, Yaw Asante Awuku, Albert Nyanga, Kyong-Mi Chang, Samuel O. Antwi, Marijana Vujković, Anurag Verma*, Kirk Joseph Wangensteen*

Table of contents

Materials and methods……………………………………………………………………...2
Supplementary table legends…………………………………....………………..….7
Fig S1……………………………………………………………………………………...11
Fig. S2…………………………………………………………………………..……...17
Fig. S3…………………………………………………………………………..…...18
Fig. S4……………………………………………………………………………......20
Fig. S5…………………………………………………………………………………..22
Fig. S6…………………………………………………………………………………..24
Fig. S7…………………………………………………………………………..…...25
Acknowledgements…………………………………………………………………....27
Supplementary references ………………………………………..……………………31

Materials and methods

Phenotype definitions

For this multi-center sequencing-based association analysis, we developed harmonized definitions of HCC cases and cancer-free controls that were adapted to the available data sources across participating biobanks. In MVP, HCC cases were identified using cancer registry data, while cancer-free controls were defined based on the absence of any malignant neoplasm ICD-9/10 diagnosis codes. In Mayo Clinic, cases were defined as individuals diagnosed with HCC based on criteria consistent with the American Association for the Study of Liver Diseases (AASLD) guidelines[1], and controls were individuals without any clinical evidence of cancer. Details about the Mayo Clinic sample collection from biobanks around the world are provided in Supplementary Table S1.

In PMBB and AOU, where cancer registry was not available, we identified HCC cases using a standardized algorithm based on the presence of at least one HCC-related ICD-9 or ICD-10 diagnosis code, with cancer-free controls defined as individuals without any malignant neoplasm ICD-9 or ICD-10 codes. The specific diagnostic codes and inclusion/exclusion criteria used for case and control classification are provided in Supplementary Table S2.

In ESCALON, a prospective multi-center study across seven medical institutions in six Latin American countries (Argentina, Brazil, Chile, Colombia, Ecuador, and Peru), participants were enrolled as part of ongoing HCC biomarker surveillance and not randomly.[2] HCC cases were identified using criteria consistent with the American Association for the Study of Liver Diseases (AASLD) guidelines, based on either histological confirmation via liver biopsy or characteristic imaging findings. For imaging-based diagnosis, multiphasic computed tomography (CT) or magnetic resonance imaging (MRI) demonstrating arterial phase hyperenhancement with venous phase washout was required. Liver biopsy was reserved for cases with inconclusive imaging results or when additional histopathological characterization was clinically indicated.

Germline genetic sequencing

Genomic DNA from participants underwent different sequencing approaches across biobanks. In PMBB, whole exome sequencing (WES) was performed on 57,170 participants across two batches using different capture products. The first batch utilized a modified version of IDT's xGen probe library while the second batch used the Twist Comprehensive Exome panel, sample collections details are outlined elsewhere.[3] Sequencing and variant calling were conducted by the Regeneron Genetics Center

(RGC) using the Twist Comprehensive Exome panel and the Illumina NovaSeq 6000 platform. FASTQ files were aligned to GRCh38 using BWA-MEM[4] using alt-aware mapping following the original quality functional equivalent (OQFE) protocol[5]. Variant calling was performed using a Parabricks-accelerated version of DeepVariant v0.10 with a custom WES model. Individual sample variant calls were generated in genomic VCF (gVCF) format and aggregated using GLnexus v1.4.3[6] with the DeepVariantWES configuration to create a joint genotyped project level VCF (pVCF). The final dataset was converted to PLINK bed/bim/fam format using PLINK v1.9[7] for downstream analyses.

In AOU, short-read whole genome sequencing (WGS) was performed on more than 414,000 participants as part of the curated data repository (CDR) version 8, sample collection details, sequencing, and variant calling details are provided here.[8] Additional variant normalization and annotation preprocessing specific to AOU data were performed as detailed in the project repository.[9]

In Mayo Clinic, whole-exome sequencing (WES) was performed using two platforms: the Regeneron "Project Generation" platform and, for Tapestry program participants, the Helix Exome+ platform. ESCALON samples were also sequenced using the Helix Exome+ platform. For samples sequenced by Regeneron, sequencing and quality control procedures have been previously described in supplementary methods.[10] Sample-level QC filtering removed samples with gender discordance, exome coverage below 80% at 20X depth, contamination exceeding 5%, or identified as duplicates.

For samples sequenced by Helix, the Exome+ assay is an augmented exome capture that additionally includes capture regions outside the exome for purposes of capturing common tag SNPs. This is intended to provide a genome-wide "backbone" of interrogated genetic variation to emulate traditional genome-wide genotyping array data typically used for genome-wide association studies. The Helix Pipeline follows the GATK best practices for variant processing with the BWA-MEM aligner. It achieves a mean SNP and indel call rate of ≥99.5% for approximately 600 genes associated with hereditary cancer, cardiovascular disease, pharmacogenomics, and carrier screening; ≥98% for roughly 3,000 genes linked to single-gene disorders; and ≥96% for the whole exome.

In MVP, deep whole-genome sequencing (WGS) was performed on 109,826 participants. Sequence reads were aligned to GRCh38 using BWA-MEM v0.7.15, alignments were compressed to CRAM. Variant calling was performed with GATK 4.1.0.0 and variant calls were generated in gVCF format. Sample-level QC filtering removed samples with per-base sequence quality score <28, GC content outside 40%-41%, <95% properly mapped reads, contamination exceeding 5%, or mean sequencing depth <18x.

Genetic Ancestry Classification

Genetic ancestry was determined using cohort-specific pre-computed principal components (PCs), all derived using the 1000 Genomes Project (1KG) as a reference panel. For All of Us, ancestry classification incorporated both 1KG and the Human Genome Diversity Project (HGDP) as reference panels. Participants were classified into six ancestry groups (European [EUR], African [AFR], Admixed American [AMR], East Asian [EAS], South Asian [SAS], and Middle Eastern [MID]) based on genetic similarity to reference population clusters in PC space. To visualize ancestry distributions, HCC cases and controls were overlaid onto cohort-level PCA plots, with HCC cases and cancer-free controls distinguished by marker shape (Fig. S6). Individuals with admixed backgrounds were assigned to the ancestry group with the highest genetic similarity. Primary analyses were conducted separately for EUR and AFR populations, with an additional combined multi-population analysis (ALL) inclusive of all six groups. The relatively low representation of EAS and SAS participants reflects the underlying demographic composition of the contributing biobanks.

Variant Annotation

Variants from all biobanks were functionally annotated using the Ensembl Variant Effect Predictor (VEP) v113.0 with consistent annotation criteria applied across datasets, except MVP which used v110. All analyses used the GRCh38 reference genome. The annotation pipeline incorporated multiple specialized plugins and databases to comprehensively characterize variant functional consequences.

Core annotation plugins included Loss-of-Function Transcript Effect Estimator (LoFTEE) for identifying high-confidence loss-of-function variants, Combined Annotation Dependent Depletion (CADD) for deleteriousness prediction, Rare Exome Variant Ensemble Learner (REVEL) for predicting the pathogenicity of missense variants, and SpliceAI for splice site prediction. Additional annotations were derived from ClinVar (accessed in October, 2024, Tables S4, S5). These include variants in the six genes designated as pathogenic or likely pathogenic (P/LP) by a majority of laboratories after

2015, when ACMG first released the modern guidelines for variant annotation.[11] These P/LP variants were included in an inclusion list (Table S4). Variants for which a majority of laboratories classified them as “uncertain significance” or “benign” were placed in an exclusion list (Table S5).

Following annotation, variants were classified into three functional categories using standardized criteria applied across all biobanks: (1) High Confidence Predicted Loss-of-Function (pLoF) variants, (2) damaging missense variants, and (3) combined pLoF+damaging missense variants. To ensure consistent variant grouping across the biobanks we used the Python script detailed in the project repository[12] to classify annotated variants into the functional bins.The detailed variant classification workflow is provided in Supplementary Table S3. The number of pLoF and damaging missense variants meeting inclusion criteria at each filtering step across all biobanks is provided in Supplementary Figure S2.

Only rare variants were retained, defined as those with maximum broad ancestry minor allele frequency ≤1% in gnomAD or absent from gnomAD. Variants were then categorized using MANE (Matched Annotation from NCBI and EMBL-EBI) transcripts into two main functional classes as outlined in Supplementary Table 3, with the third category representing the union of these classes.

pLoF variants included: ClinVar pathogenic or likely pathogenic variants (curated post-2015), LOFTEE high-confidence loss-of-function variants, VEP high-impact consequences (transcript_ablation, stop_gained, frameshift_variant, stop_lost, start_lost, transcript_amplification, feature_elongation, feature_truncation), and splice site variants with SpliceAI scores ≥0.2. Variants classified as ClinVar variants of uncertain significance, likely benign, or benign were excluded. The curated ClinVar inclusion criteria are detailed in Supplementary Table S4, and exclusion criteria are provided in Supplementary Table S5.

Damaging missense protein-altering variants included missense, start-loss, stop-loss, in-frame indel, or splicing variants (not already classified as high-confidence pLoF) meeting at least one of the following criteria: REVEL score ≥0.773, CADD

score ≥28.1, SpliceAI score ≥0.2, or LOFTEE low-confidence loss-of-function classification. A complete list of rare variants included in the six-gene burden analysis across all four biobanks for the ALL population group is provided in Supplementary Table S6.

Statistical analysis

Based on previous studies detecting germline variants in *FANCA*, *BRIP1*, *CHEK2*, and *BRCA2, MSH6* and *PMS2* among HCC patients across independent cohorts, we focused our rare variant burden analysis on these six genes. This approach was chosen to maximize statistical power for detecting associations while minimizing the multiple testing burden. Gene-burden association tests were performed within each biobank to assess associations between germline variants in these six genes and HCC risk. Analyses were conducted separately for European ancestry (EUR), African ancestry (AFR), and combined multi-ancestry (ALL) populations using the SAIGE-GENE+ framework. All models included age, sex, and principal components of genetic ancestry as covariates (10 PCs for MVP, AOU, and Mayo Clinic; 6 PCs for PMBB). Three maximum minor allele frequency (MAF) thresholds were tested for each gene: 1%, 0.1%, and 0.01%.

After obtaining study-level results, to increase the number of cases and controls and gain statistical power we performed fixed effects inverse variance weighted meta-analysis for EUR and AFR across the four biobanks and random effects inverse variance weighted meta-analysis for ALL. Meta-analyses were conducted using the metafor package in R. Statistical significance was determined using a False Discovery Rate (FDR) correction with the Benjamini-Hochberg method at a cutoff of 0.05. Complete association results for EUR, AFR, and ALL populations are provided in Supplementary Tables S7, S8, and S9, respectively.

Supplementary table legends

- Table S1. Mayo Clinic Site Breakdown by Population Group. Sample distribution across different data sources and sites for Mayo Clinic stratified by population groups and case-control status. Data are presented for three population groups: European (EUR), Admixed American (AMR), and African (AFR) populations. Numbers represent individual participants classified as either hepatocellular carcinoma (HCC) cases or cancer-free controls.
- Table S2. Phenotyping algorithms and criteria for hepatocellular carcinoma case and control identification across biobanks. Inclusion and exclusion criteria used for case-control classification across participating biobanks. HCC cases were identified using ICD (International Classification of Diseases) codes in PMBB and AOU, clinical diagnosis in Mayo Clinic, and cancer registry data in MVP. Cancer-free controls excluded individuals with any cancer diagnosis. Specific ICD codes for HCC inclusion and exclusion of other hepatobiliary cancers ensured accurate phenotyping across biobanks. PMBB = Penn Medicine BioBank; AOU = All of Us Research Program; MVP = Million Veteran Program.
- Table S3. Inclusion and exclusion criteria for binning variants into functional categories. Filtering criteria and functional annotation pipeline used to classify genetic variants for burden testing. Variants were filtered by frequency (MAF $\leq$ 1% in gnomAD) and analyzed using MANE (Matched Annotation from NCBI and EMBL-EBI) transcripts. Three functional categories were defined: High Confidence predicted Loss-of-Function (pLoF) variants identified through ClinVar pathogenic classifications, high-impact VEP consequences, or SpliceAI scores $\geq$ 0.2; Damaging Missense variants determined by REVEL $\geq$ 0.773, CADD $\geq$ 28.1, or SpliceAI $\geq$ 0.2; and a combined category including both pLoF and damaging missense variants. ClinVar variants classified as variants of uncertain significance (VUS), likely benign, or benign were excluded from high confidence pLoF categories to ensure stringent functional impact prediction.
- Table S4. Curated ClinVar Inclusion list. Curated list of ClinVar pathogenic and likely pathogenic variants used for inclusion in the pLoF classification. This list includes variants with review status annotations curated after 2015 and expert-reviewed classifications that meet ACMG criteria for pathogenic or likely pathogenic designations. These variants were prioritized for inclusion in gene burden analyses based on their established clinical significance.
- Table S5. Curated ClinVar Exclusion list. Curated list of ClinVar variants classified as variants of uncertain significance (VUS), likely benign, or benign that were excluded from pLoF classification. These variants were excluded to ensure specificity and reduce potential false positive associations from variants lacking sufficient evidence of pathogenicity.

- Table S6. Rare variants included across four biobanks for six-gene burden analysis in ALL populations. Rare variants meeting pLoF or damaging missense criteria that were tested in the gene burden analysis across Penn Medicine BioBank (PMBB), Mayo Clinic, All of Us (AOU), and Million Veteran Program (MVP). For pLoF variants, ClinVar annotations derive from the curated Pathogenic/Likely Pathogenic inclusion list (Supplementary Table S4), with "NOT_FOUND_IN_CLINVAR" indicating absence from this list. For damaging missense variants, ClinVar annotations derive from VEP annotation files, with "-" indicating no ClinVar classification. Values <5 for MAC_Case and MAC_Control are censored as "<5" for privacy protection.
- Table S7. Fixed-effects meta-analysis results for six-gene burden associations with HCC risk in EUR populations. Results from fixed-effects meta-analysis across four biobanks (PMBB, AOU, Mayo Clinic, MVP) showing gene burden associations with HCC risk in European populations. Analysis includes pLoF, damaging missense, and pLoF+damaging missense variants across six DNA repair genes at three MAF thresholds: 1%, 0.1%, and 0.01%. Results indicate FDR significance status and corresponding FDR q-values after Benjamini-Hochberg correction for multiple testing.
- Table S8. Fixed-effects meta-analysis results for six-gene burden associations with HCC risk in AFR populations. Results from fixed-effects meta-analysis across three biobanks (PMBB, AOU, MVP) showing gene burden associations with HCC risk in African populations. Analysis includes pLoF, damaging missense, and pLoF+damaging missense variants across six DNA repair genes at three MAF thresholds: 1%, 0.1%, and 0.01%. Results indicate FDR significance status and corresponding FDR q-values after Benjamini-Hochberg correction for multiple testing.
- Table S9. Random-effects meta-analysis results for six-gene burden associations with HCC risk in ALL populations. Results from random-effects meta-analysis across four biobanks (PMBB, AOU, Mayo Clinic, MVP) showing gene burden associations with HCC risk in ALL populations (including EUR, AFR, AMR, EAS, SAS, MID). Analysis includes pLoF, damaging missense, and pLoF+damaging missense variants across six DNA repair genes at three MAF thresholds: 1%, 0.1%, and 0.01%. Results indicate FDR significance status and corresponding FDR q-values after Benjamini-Hochberg correction for multiple testing.
- Table S10. Cross-biobank average of carrier frequencies of rare variant categories in ALL HCC cases and cancer-free controls. Cross-biobank average carrier frequencies of rare variant categories in ALL HCC cases and cancer-free controls across six DNA repair genes. Values represent the mean carrier frequency averaged across four biobanks (PMBB, AOU, Mayo Clinic, MVP) and are reported as case frequency | control frequency for three variant categories:

pLoF, damaging missense, and pLoF+damaging missense. Variants are restricted to those included in the SAIGE-GENE+ burden test group files.

- Table S11. Cross-biobank average of ancestry-stratified rare variant carrier frequencies in HCC cases and cancer-free controls. Cross-biobank average carrier frequencies of rare pathogenic variant categories in HCC cases and cancer-free controls, stratified by genetic ancestry (EUR, AFR, AMR, EAS, SAS, MID) across six DNA repair genes. Values represent mean carrier frequency across up to four biobanks (PMBB, AOU, Mayo Clinic, MVP), reported as case frequency | control frequency for three variant categories: pLoF, damaging missense, and pLoF+damaging missense. Variants are restricted to those included in the SAIGE-GENE+ burden test group files. When a biobank reported N/A for a given ancestry-gene-category combination, reflecting absence of that ancestry cohort rather than zero frequency, it was excluded from the average. Averages were calculated using only available data. Per-biobank frequencies are not reported to protect participant privacy. Values of 0.000 indicate no observed carriers or frequencies too small to display at three decimal places.
- Table S12. Baseline disease characteristics for HCC cases and cancer-free controls across biobanks. Baseline liver disease characteristics among HCC cases and cancer-free controls across four biobanks (PMBB, AOU, Mayo Clinic, MVP). Values are reported as number (percentage) for cases and controls for each condition, including hepatitis B virus (HBV), hepatitis C virus (HCV), alcohol-related liver disease, and MASH/MASLD. Percentages are calculated within each biobank and case-control group. A total of 93 EAS, SAS, and other participants were excluded from Mayo Clinic due to unanalyzable SAIGE results.
- Table S13. Inclusion and exclusion criteria for binning variants into functional categories for ClinVar sensitivity analysis. Variants were filtered by minor allele frequency and transcript, then classified into functional categories (high-confidence pLoF, damaging missense, and combined) using ClinVar pathogenicity and VEP annotations. This sensitivity analysis restricts to higher-confidence pathogenic variants to clarify the contribution of damaging missense variants to gene-based associations.
- Table S14. Random-effects meta-analysis of rare variant carrier enrichment for liver disease comorbidities among HCC cases in six DNA repair genes across biobanks. Carrier status was defined as carrying at least one qualifying rare variant (pLoF, damaging missense, and combined), restricted to HCC cases across PMBB, AOU, Mayo Clinic, and MVP. Liver disease comorbidities: hepatitis B virus (HBV), hepatitis C virus (HCV), alcohol-related liver disease, and MASH/MASLD. Pooled odds ratios were estimated using the DerSimonian-Laird random-effects model. Q: Cochran's Q; I^2: between-study heterogeneity; τ^2: between-study variance.

- Table S15. Random-effects meta-analysis results for *BRCA2* and *MSH6* gene burden associations with HCC risk in ALL populations after adjustment for liver disease comorbidities. Results from random-effects meta-analysis across four biobanks (PMBB, AOU, Mayo Clinic, MVP) showing *MSH6* and *BRCA2* gene burden associations with HCC risk in ALL populations (including EUR, AFR, AMR, EAS, SAS, MID). Analysis includes pLoF, damaging missense, and pLoF+damaging missense variants at three MAF thresholds: 1%, 0.1%, and 0.01%. Results indicate FDR significance status and corresponding FDR q-values after Benjamini-Hochberg correction for multiple testing.
- Table S16. Random-effects sex-stratified meta-analysis results of six-gene burden associations with HCC risk. Results from random-effects meta-analysis across four biobanks (PMBB, AOU, Mayo Clinic, MVP) showing gene burden associations with HCC risk stratified by sex (male and female) in ALL populations (including EUR, AFR, AMR, EAS, SAS, MID). Analysis includes pLoF, damaging missense, and pLoF+damaging missense variants across six DNA repair genes at three MAF thresholds: 1%, 0.1%, and 0.01%. Results indicate FDR significance status and corresponding FDR q-values after Benjamini-Hochberg correction for multiple testing.
- Table S17. Random-effects meta-analysis of co-occurring cancer enrichment among HCC cases carrying rare variants in six DNA repair genes. Within each biobank (PMBB, AOU, Mayo Clinic, MVP), Fisher's exact test was used to assess enrichment of breast, ovarian, pancreatic, or prostate cancer diagnoses among HCC cases carrying rare variants (pLoF, damaging missense, or pLoF+damaging missense; MAF ≤ 1%) compared to non-carriers. Cross-biobank pooling used inverse variance-weighted meta-analysis with a DerSimonian-Laird random-effects model. Log odds ratios and standard errors were derived from 2×2 contingency tables per biobank. Columns include: gene, annotation mask, cancer type, number of contributing biobanks, total carriers, Cochran's Q statistic, I^2, Tau^2, pooled odds ratio (RE_OR), 95% confidence interval bounds, Z-value, p-value, FDR q-value (Benjamini-Hochberg), and FDR significance.

Fig S1. Summary of variant counts by biobank showing pLoF and damaging missense classifications. Bar plots showing the number of variants in six DNA repair genes (*BRCA2*, *BRIP1*, *CHEK2*, *FANCA*, *MSH6*, *PMS2*) that meet inclusion criteria at each filtering step, stratified by biobank (PMBB, AOU, Mayo Clinic, MVP; colors shown in legend) and functional annotation category (pLoF and damaging missense). Left panels show predicted loss-of-function (pLoF) variant counts; right panels show damaging missense variant counts. The "Total" bar represents final variant counts after applying all filtering criteria described in Table S3. Results demonstrate differences in variant detection across biobanks, with whole genome sequencing biobanks (AOU, MVP) identifying more variants overall than whole exome sequencing biobanks (PMBB, Mayo Clinic).

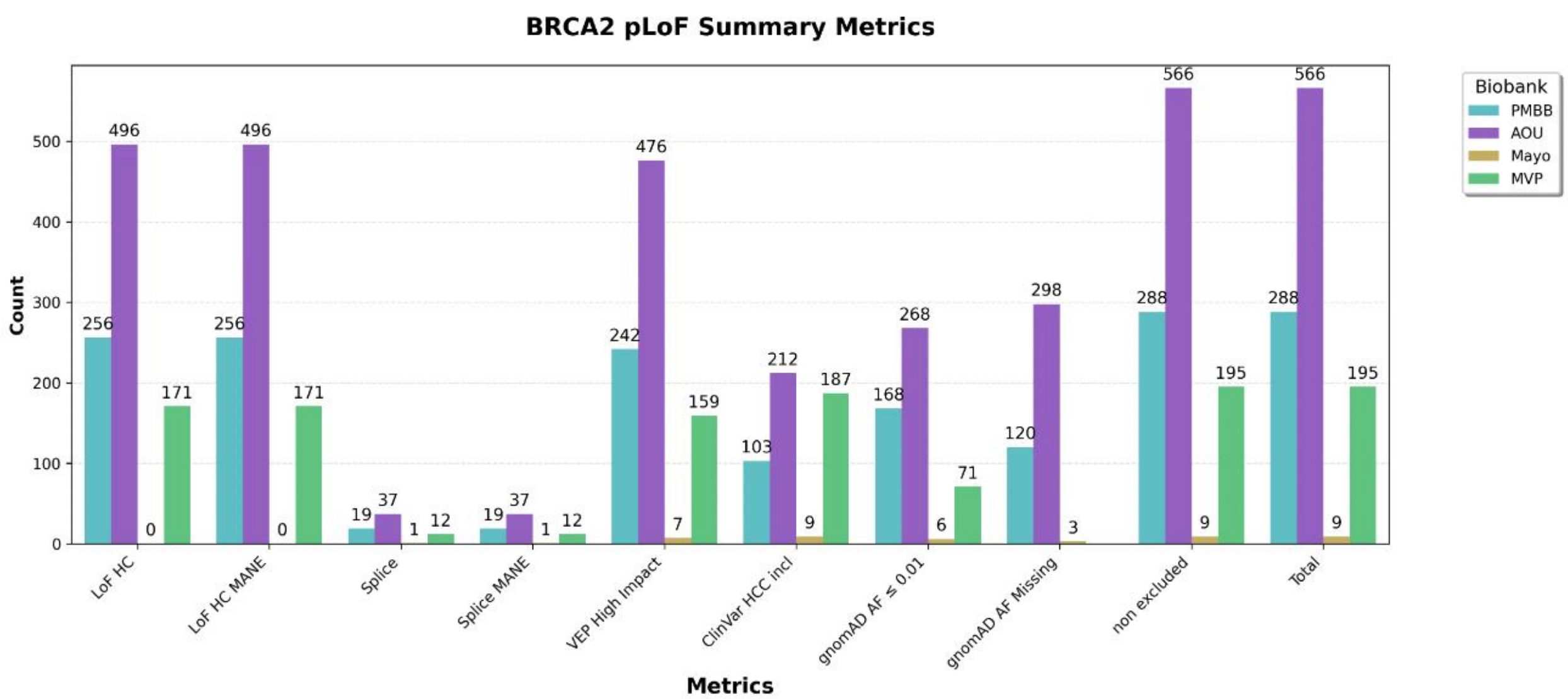

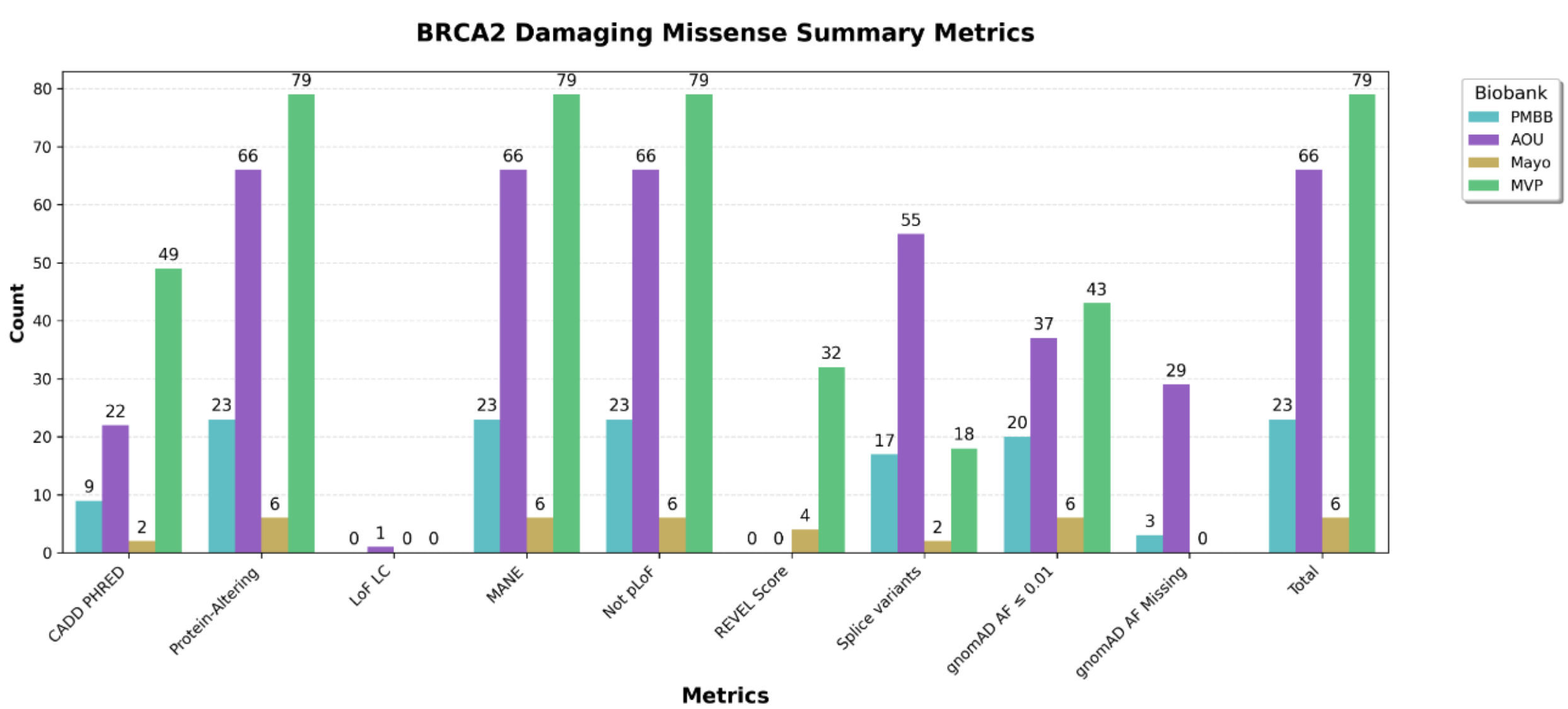

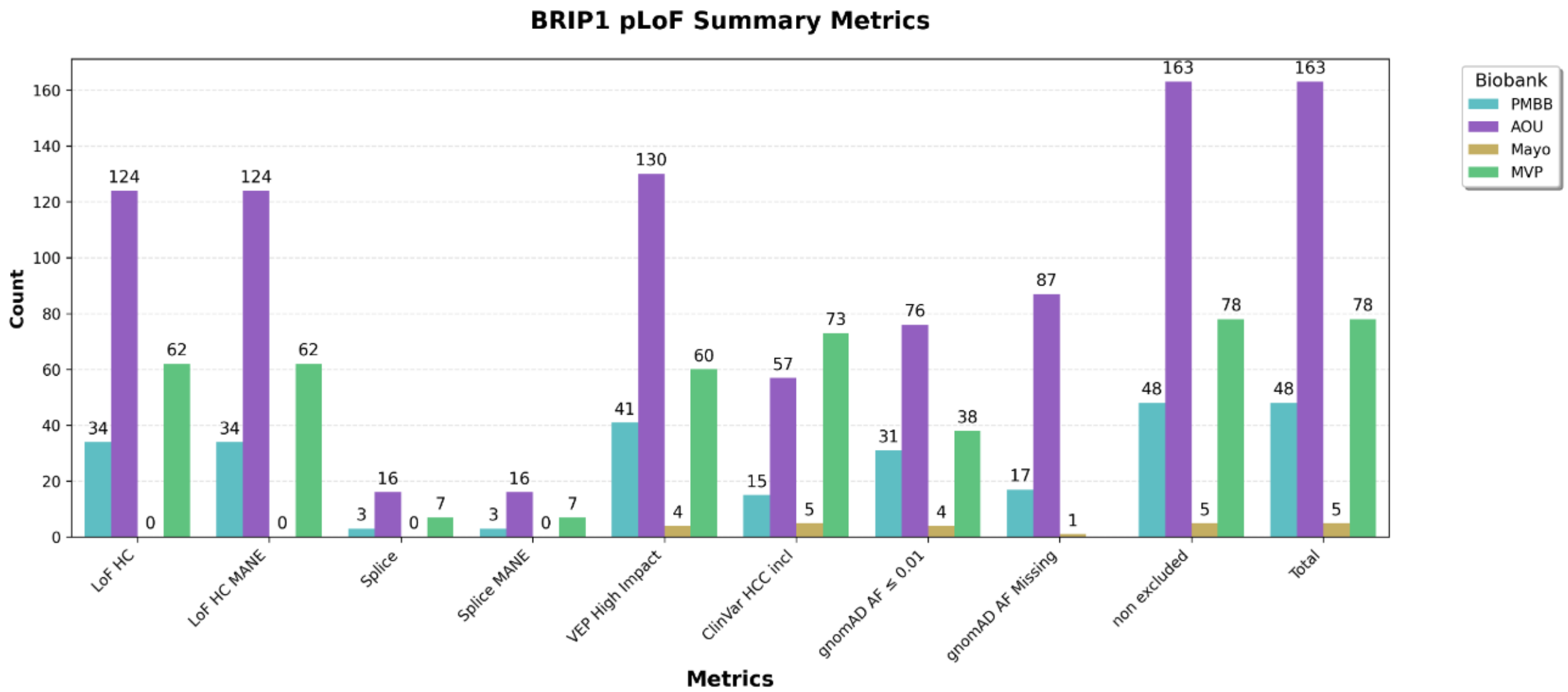
BRIP1 pLoF Summary Metrics
Count
Metrics
Biobank
PMBB
AOU
Mayo
MVP
LoF HC
LoF HC MANE
Splice
Splice MANE
VEP High Impact
ClinVar HCC incl
gnomAD AF ≤ 0.01
gnomAD AF Missing
non excluded
Total

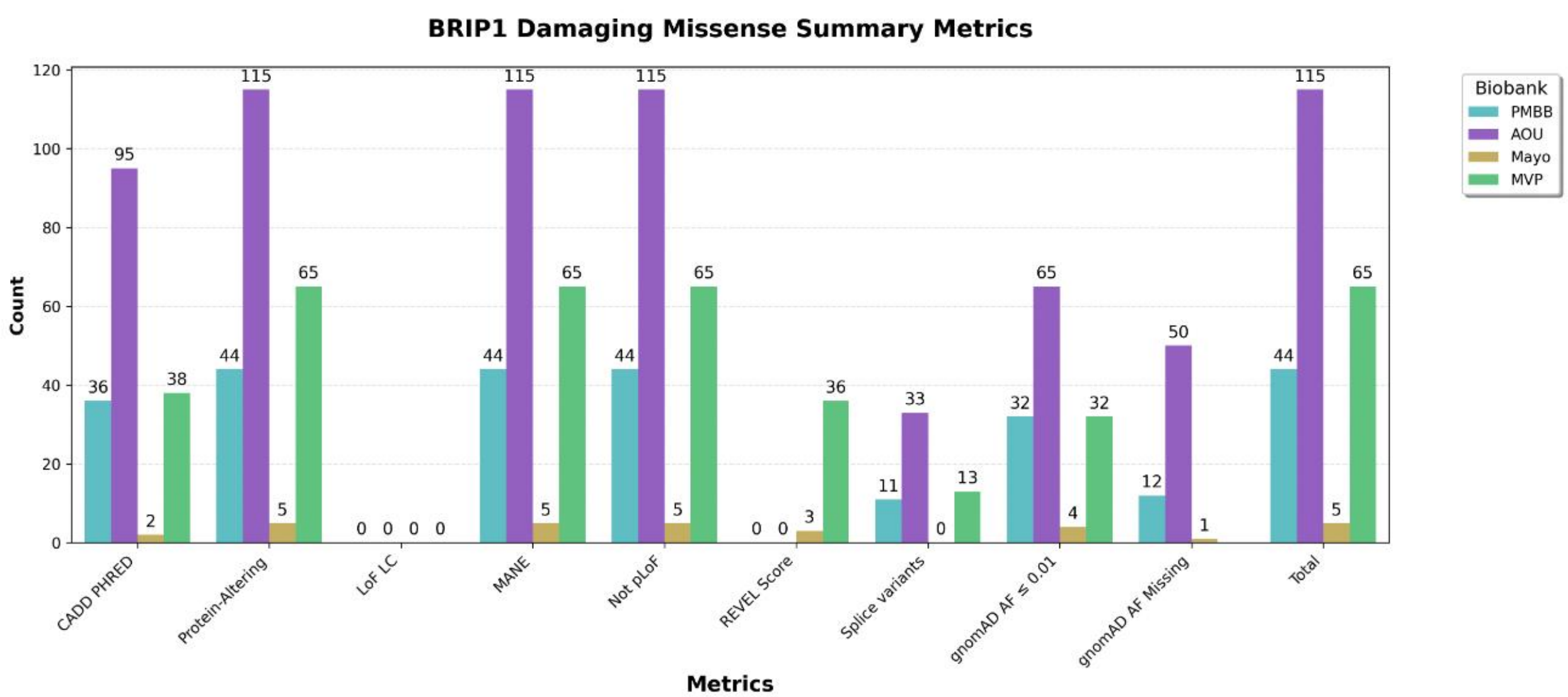
BRIP1 Damaging Missense Summary Metrics
Count
Metrics
Biobank
PMBB
AOU
Mayo
MVP
CADD PHRED
Protein-Altering
LoF LC
MANE
Not pLoF
REVEL Score
Splice variants
gnomAD AF ≤ 0.01
gnomAD AF Missing
Total

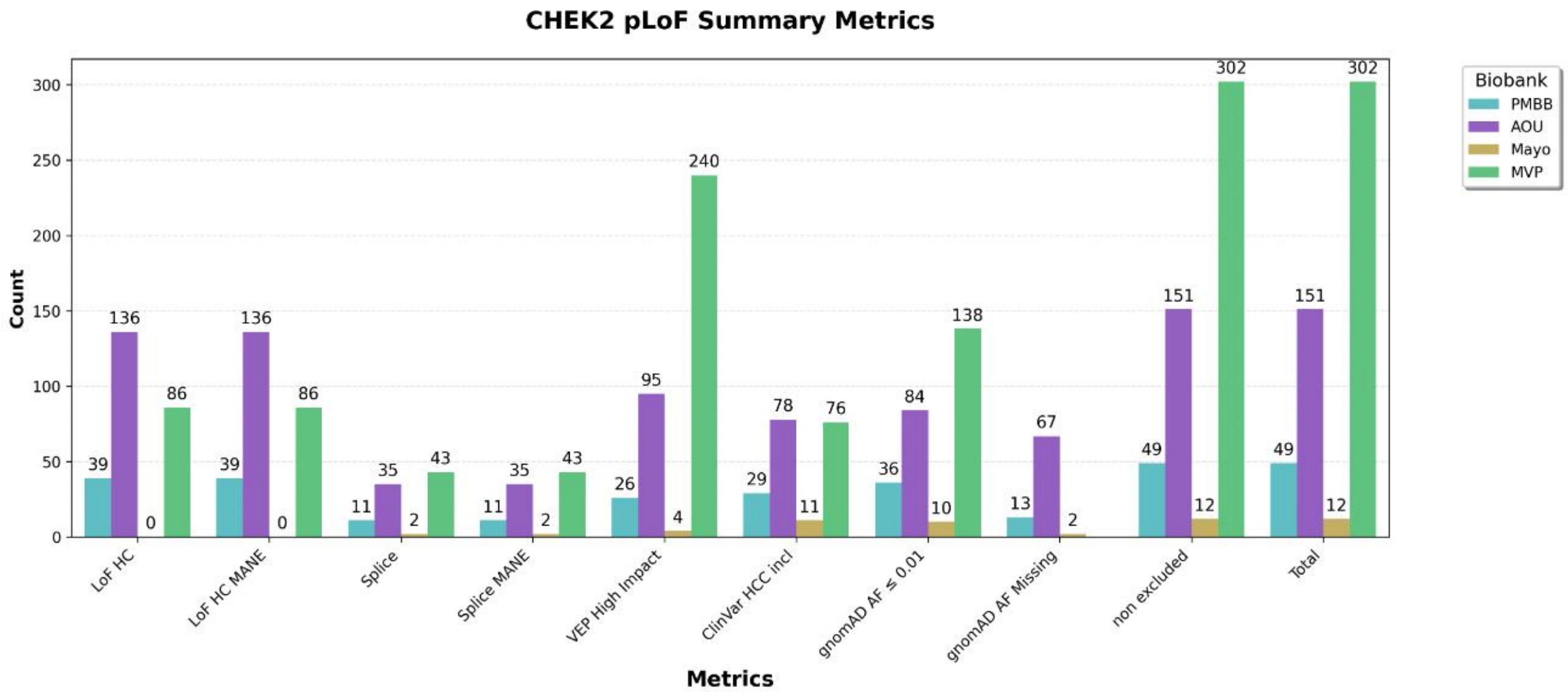
CHEK2 pLoF Summary Metrics
Count
Metrics
Biobank
PMBB
AOU
Mayo
MVP
LoF HC
LoF HC MANE
Splice
Splice MANE
VEP High Impact
ClinVar HCC incl
gnomAD AF ≤ 0.01
gnomAD AF Missing
non excluded
Total
39 136 0 86
39 136 0 86
11 35 2 43
11 35 2 43
26 95 4 240
29 78 11 76
36 84 10 138
13 67 2
49 151 12 302
49 151 12 302

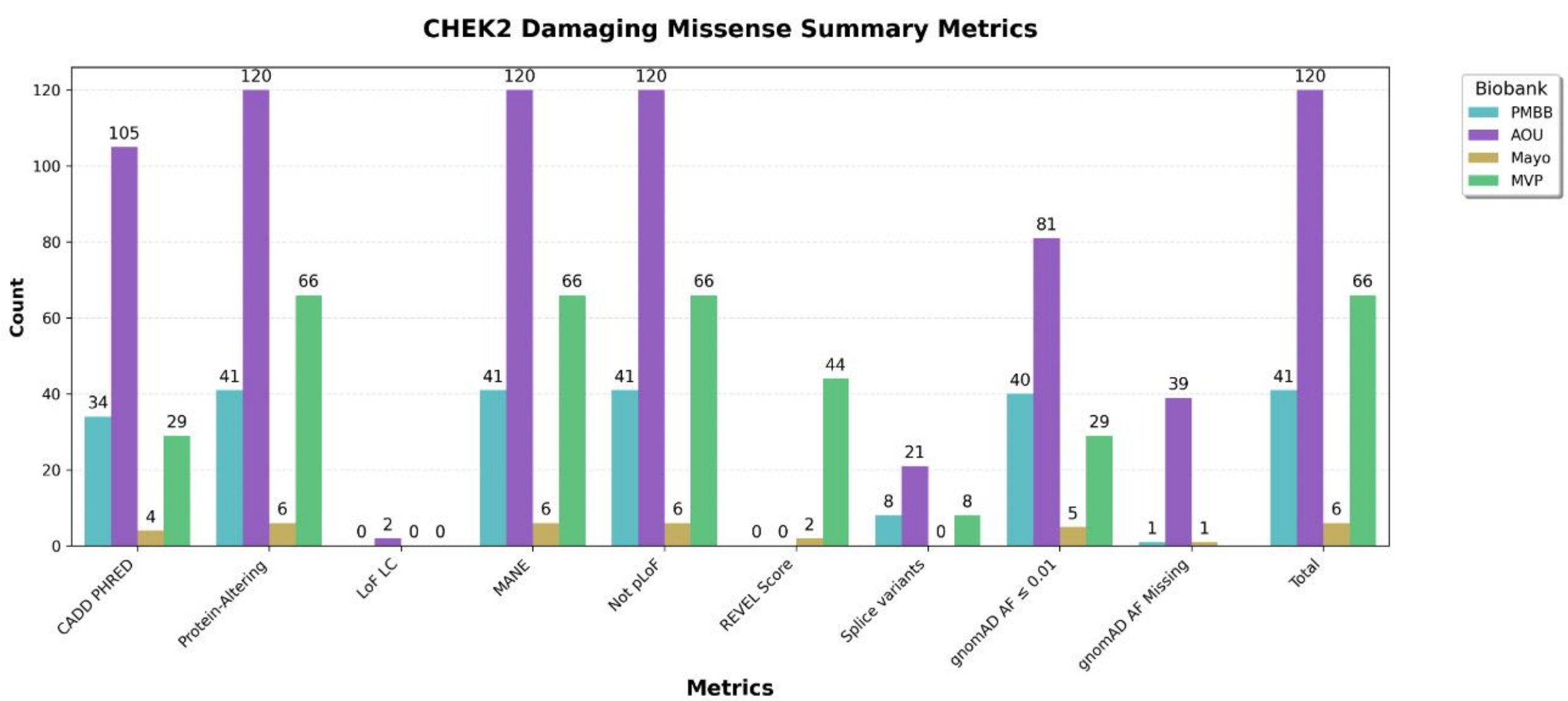
CHEK2 Damaging Missense Summary Metrics
Count
Metrics
Biobank
PMBB
AOU
Mayo
MVP
CADD PHRED
Protein-Altering
LoF LC
MANE
Not pLoF
REVEL Score
Splice variants
gnomAD AF ≤ 0.01
gnomAD AF Missing
Total
34 105 4 29
41 120 6 66
0 2 0 0
41 120 6 66
41 120 6 66
0 0 2 44
8 21 0 8
40 81 5 29
1 39 1
41 120 6 66

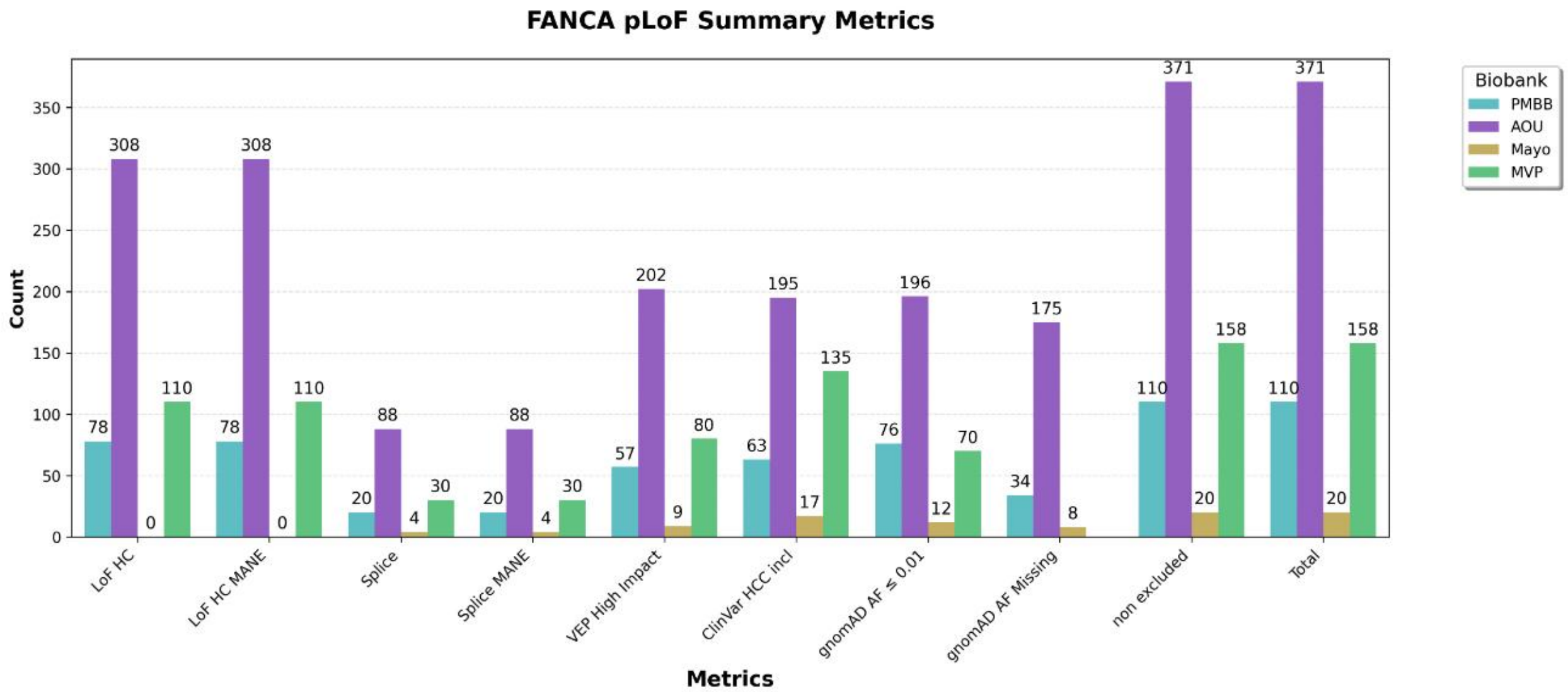

FANCA pLoF Summary Metrics
Biobank
PMBB
AOU
Mayo
MVP
Count
Metrics
LoF HC
LoF HC MANE
Splice
Splice MANE
VEP High Impact
ClinVar HCC incl
gnomAD AF ≤ 0.01
gnomAD AF Missing
non excluded
Total
78 308 0 110
78 308 0 110
20 88 4 30
20 88 4 30
57 202 9 80
63 195 17 135
76 196 12 70
34 175 8
110 371 20 158
110 371 20 158

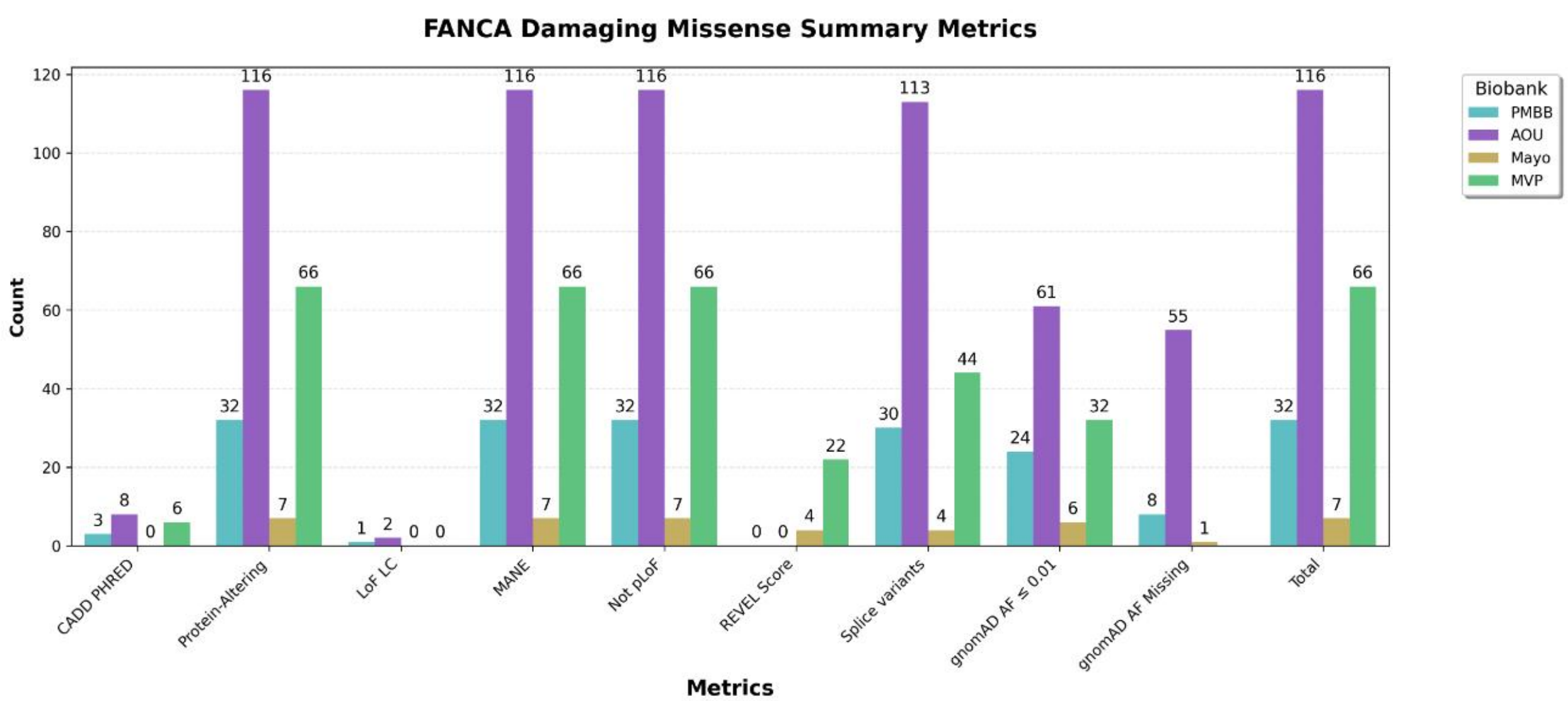

FANCA Damaging Missense Summary Metrics
Biobank
PMBB
AOU
Mayo
MVP
Count
Metrics
CADD PHRED
Protein-Altering
LoF LC
MANE
Not pLoF
REVEL Score
Splice variants
gnomAD AF ≤ 0.01
gnomAD AF Missing
Total
3 8 0 6
32 116 7 66
1 2 0 0
32 116 7 66
32 116 7 66
0 0 4 22
30 113 4 44
24 61 6 32
8 55 1
32 116 7 66

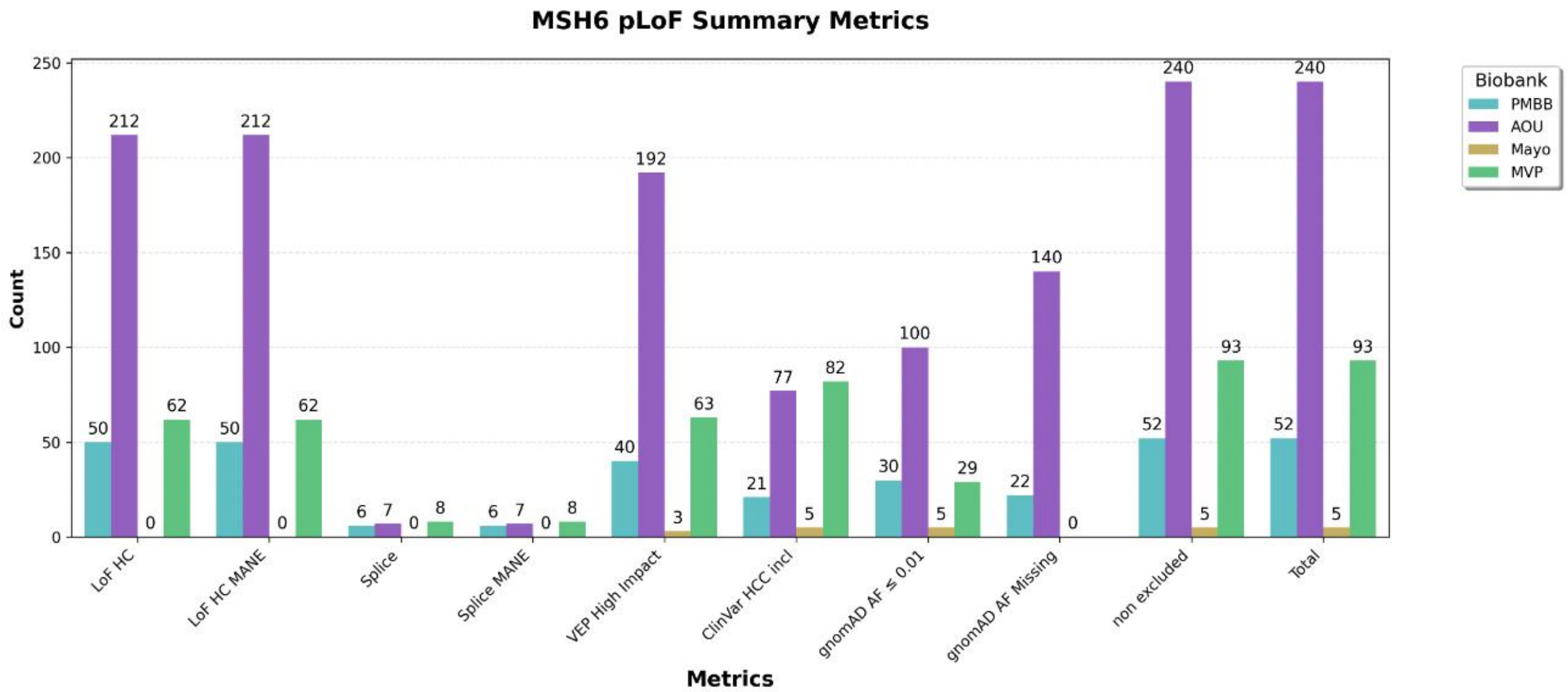
MSH6 pLoF Summary Metrics
Count
Metrics
Biobank
PMBB
AOU
Mayo
MVP
LoF HC
LoF HC MANE
Splice
Splice MANE
VEP High Impact
ClinVar HCC incl
gnomAD AF ≤ 0.01
gnomAD AF Missing
non excluded
Total

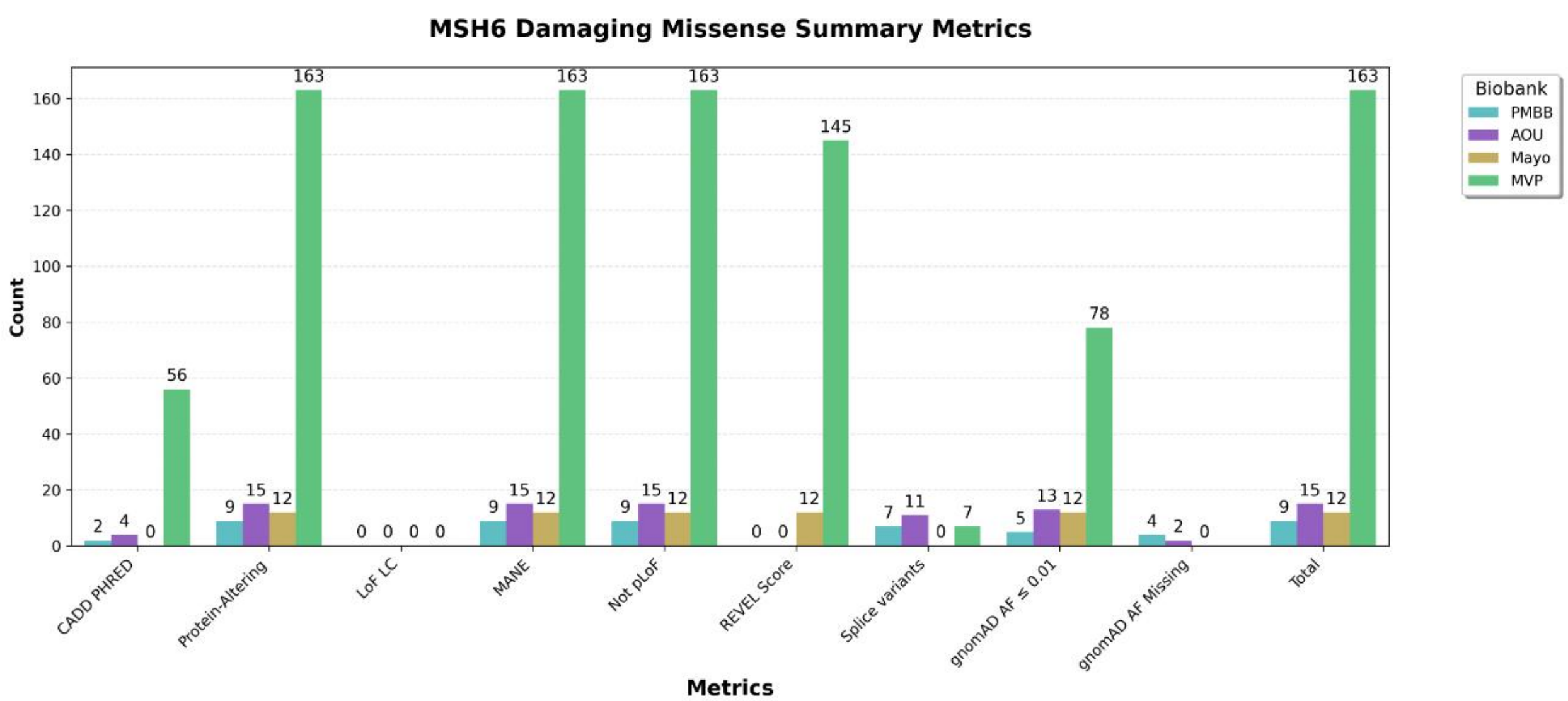
MSH6 Damaging Missense Summary Metrics
Count
Metrics
Biobank
PMBB
AOU
Mayo
MVP
CADD PHRED
Protein-Altering
LoF LC
MANE
Not pLoF
REVEL Score
Splice variants
gnomAD AF ≤ 0.01
gnomAD AF Missing
Total

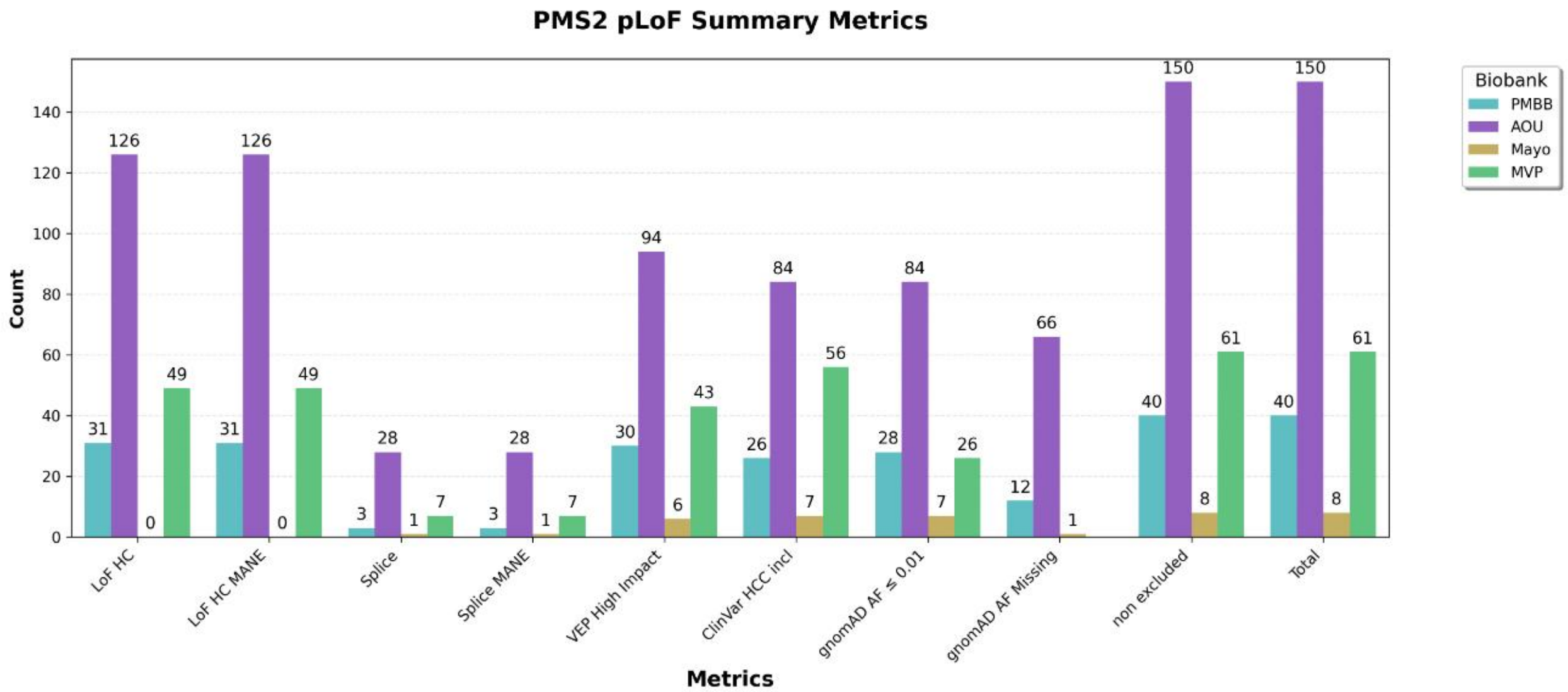

PMS2 pLoF Summary Metrics
Count
Metrics
Biobank
PMBB
AOU
Mayo
MVP
LoF HC
LoF HC MANE
Splice
Splice MANE
VEP High Impact
ClinVar HCC incl
gnomAD AF ≤ 0.01
gnomAD AF Missing
non excluded
Total

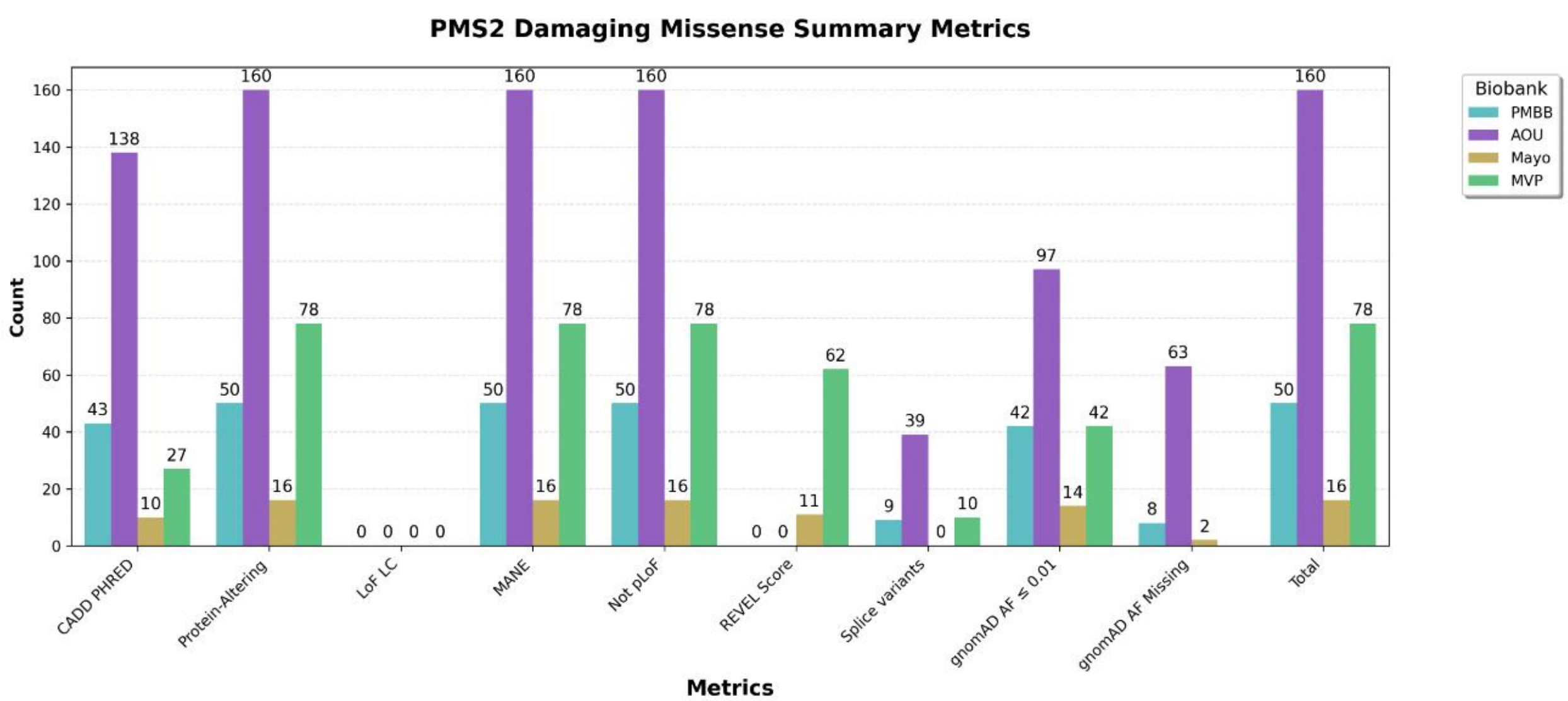

PMS2 Damaging Missense Summary Metrics
Count
Metrics
Biobank
PMBB
AOU
Mayo
MVP
CADD PHRED
Protein-Altering
LoF LC
MANE
Not pLoF
REVEL Score
Splice variants
gnomAD AF ≤ 0.01
gnomAD AF Missing
Total

Fig S2. Meta-analysis forest plot of *BRCA2* and *MSH6* gene burden associations with HCC risk in ALL populations after adjustment for liver disease comorbidities. Forest plots of odds ratios and 95% confidence intervals for gene burden associations with HCC risk across four biobanks (PMBB, MVP, AOU, Mayo Clinic) for: (A) MSH6 (B) BRCA2. Asterisks (*) indicate FDR < 0.05 (Benjamini-Hochberg). P-values from inverse variance weighted random-effects meta-analysis. Colored squares represent per-biobank odds ratios (OR); horizontal lines indicate 95% confidence intervals. The pink diamond represents the pooled random-effects meta-analytic estimate.

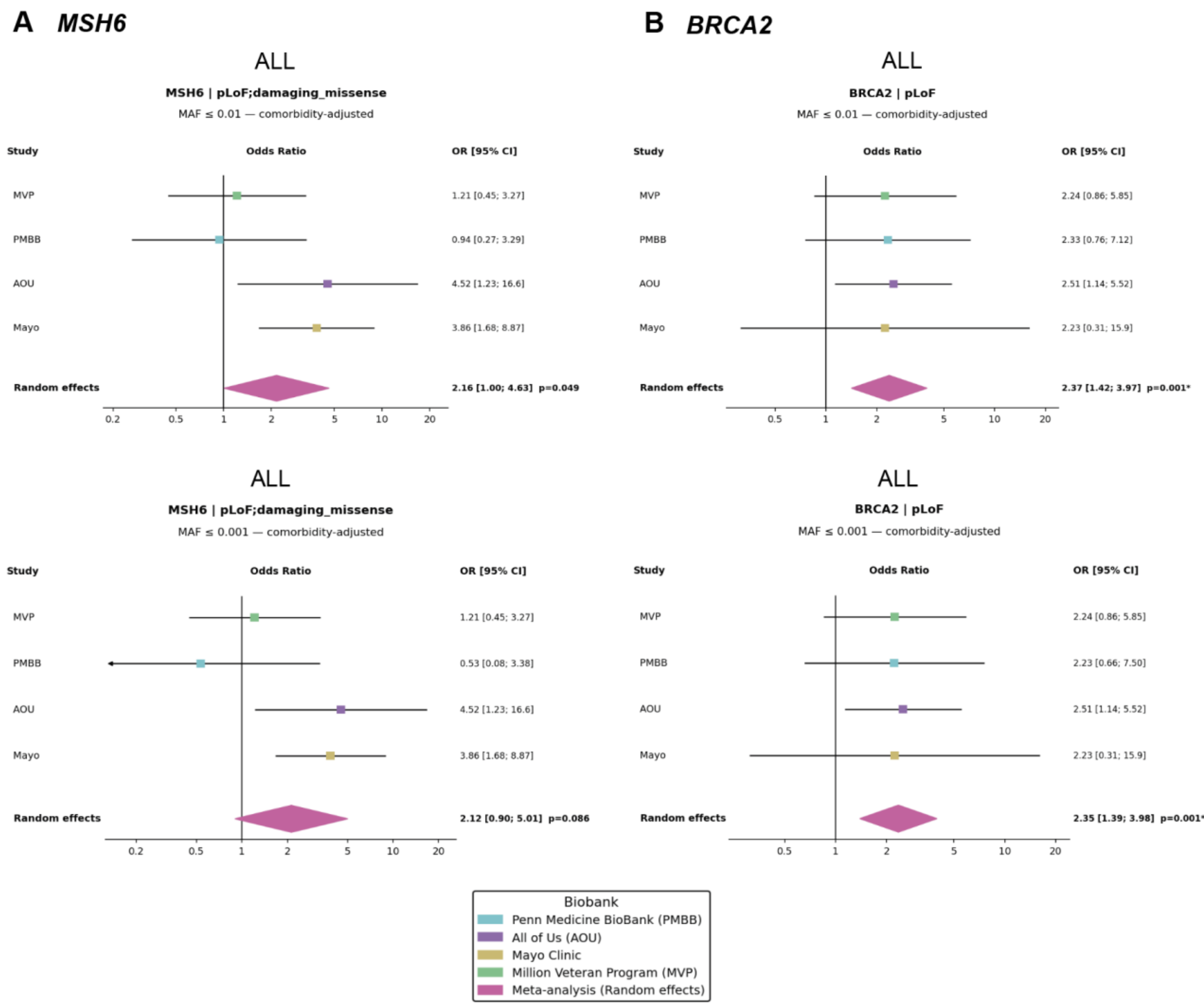

Fig S3. Meta-analysis forest plot of liver disease comorbidity enrichment in *BRCA2* and *MSH6* rare variant carriers among HCC cases in ALL populations. Forest plots of odds ratios and 95% confidence intervals comparing liver disease comorbidity prevalence in rare variant carriers versus non-carriers within HCC cases across four biobanks (PMBB, MVP, AOU, Mayo Clinic) for (A) *MSH6* and (B) *BRCA2*. Per-biobank estimates were derived using Fisher's exact test. Pooled odds ratios were estimated under a DerSimonian-Laird random-effects model.

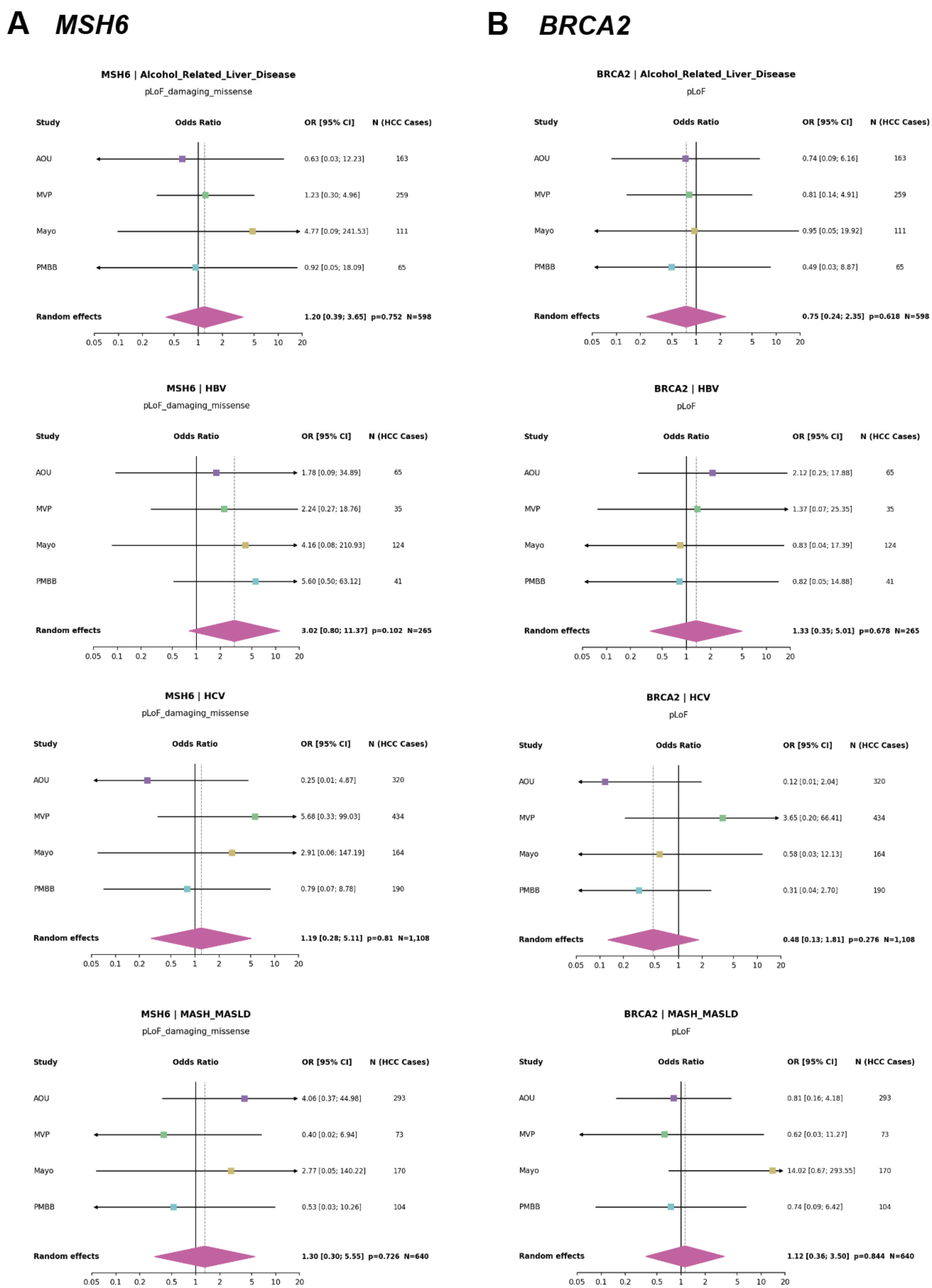
A MSH6
B BRCA2
MSH6 | Alcohol_Related_Liver_Disease
pLoF_damaging_missense
Study
Odds Ratio
OR [95% CI]
N (HCC Cases)
AOU
0.63 [0.03; 12.23]
163
MVP
1.23 [0.30; 4.96]
259
Mayo
4.77 [0.09; 241.53]
111
PMBB
0.92 [0.05; 18.09]
65
Random effects
1.20 [0.39; 3.65] p=0.752 N=598
0.05 0.1 0.2 0.5 1 2 5 10 20
BRCA2 | Alcohol_Related_Liver_Disease
pLoF
Study
Odds Ratio
OR [95% CI]
N (HCC Cases)
AOU
0.74 [0.09; 6.16]
163
MVP
0.81 [0.14; 4.91]
259
Mayo
0.95 [0.05; 19.92]
111
PMBB
0.49 [0.03; 8.87]
65
Random effects
0.75 [0.24; 2.35] p=0.618 N=598
0.05 0.1 0.2 0.5 1 2 5 10 20
MSH6 | HBV
pLoF_damaging_missense
Study
Odds Ratio
OR [95% CI]
N (HCC Cases)
AOU
1.78 [0.09; 34.89]
65
MVP
2.24 [0.27; 18.76]
35
Mayo
4.16 [0.08; 210.93]
124
PMBB
5.60 [0.50; 63.12]
41
Random effects
3.02 [0.80; 11.37] p=0.102 N=265
0.05 0.1 0.2 0.5 1 2 5 10 20
BRCA2 | HBV
pLoF
Study
Odds Ratio
OR [95% CI]
N (HCC Cases)
AOU
2.12 [0.25; 17.88]
65
MVP
1.37 [0.07; 25.35]
35
Mayo
0.83 [0.04; 17.39]
124
PMBB
0.82 [0.05; 14.88]
41
Random effects
1.33 [0.35; 5.01] p=0.678 N=265
0.05 0.1 0.2 0.5 1 2 5 10 20
MSH6 | HCV
pLoF_damaging_missense
Study
Odds Ratio
OR [95% CI]
N (HCC Cases)
AOU
0.25 [0.01; 4.87]
320
MVP
5.68 [0.33; 99.03]
434
Mayo
2.91 [0.06; 147.19]
164
PMBB
0.79 [0.07; 8.78]
190
Random effects
1.19 [0.28; 5.11] p=0.81 N=1,108
0.05 0.1 0.2 0.5 1 2 5 10 20
BRCA2 | HCV
pLoF
Study
Odds Ratio
OR [95% CI]
N (HCC Cases)
AOU
0.12 [0.01; 2.04]
320
MVP
3.65 [0.20; 66.41]
434
Mayo
0.58 [0.03; 12.13]
164
PMBB
0.31 [0.04; 2.70]
190
Random effects
0.48 [0.13; 1.81] p=0.276 N=1,108
0.05 0.1 0.2 0.5 1 2 5 10 20
MSH6 | MASH_MASLD
pLoF_damaging_missense
Study
Odds Ratio
OR [95% CI]
N (HCC Cases)
AOU
4.06 [0.37; 44.98]
293
MVP
0.40 [0.02; 6.94]
73
Mayo
2.77 [0.05; 140.22]
170
PMBB
0.53 [0.03; 10.26]
104
Random effects
1.30 [0.30; 5.55] p=0.726 N=640
0.05 0.1 0.2 0.5 1 2 5 10 20
BRCA2 | MASH_MASLD
pLoF
Study
Odds Ratio
OR [95% CI]
N (HCC Cases)
AOU
0.81 [0.16; 4.18]
293
MVP
0.62 [0.03; 11.27]
73
Mayo
14.02 [0.67; 293.55]
170
PMBB
0.74 [0.09; 6.42]
104
Random effects
1.12 [0.36; 3.50] p=0.844 N=640
0.05 0.1 0.2 0.5 1 2 5 10 20

Fig S4. Age at HCC diagnosis in *BRCA2* and *MSH6* rare variant carriers versus non-carriers among HCC cases in ALL populations. Mean age at hepatocellular carcinoma (HCC) diagnosis for carriers of rare variants in BRCA2 and MSH6 compared to non-carriers, stratified by variant category (pLoF, damaging missense, pLoF+damaging missense). Age at HCC diagnosis was defined as the interval from birth to the earliest recorded HCC diagnosis code within each biobank (PMBB, AOU, Mayo Clinic, MVP). Within each biobank, carriers were compared to non-carriers using Welch's two-sample t-test (two-sided, unequal variances). Bars represent the sample-size–weighted pooled mean age across biobanks; error bars indicate the pooled standard deviation. Δ denotes the meta-analytic mean age difference (carriers − non-carriers) estimated by the DerSimonian–Laird random-effects model. A negative Δ indicates earlier HCC diagnosis in carriers. Statistical significance was assessed using Benjamini–Hochberg false discovery rate (FDR) correction across all gene–category groups tested ($q < 0.05$). Carrier groups with fewer than 5 individuals ($n < 5$) are displayed as hatched bars with counts not shown to protect participant privacy.

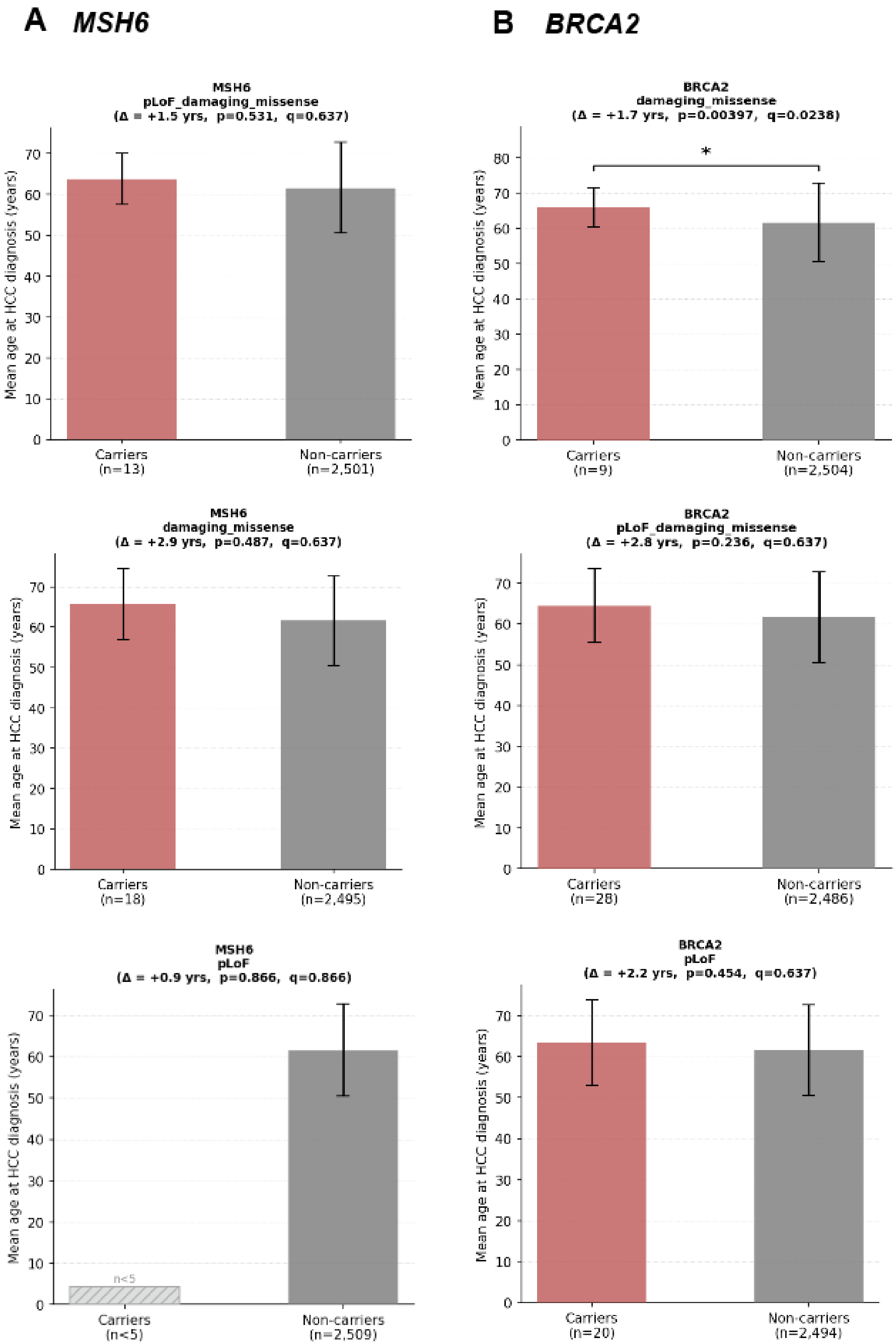
A MSH6
B BRCA2
MSH6
pLoF_damaging_missense
(Δ = +1.5 yrs, p=0.531, q=0.637)
Mean age at HCC diagnosis (years)
Carriers (n=13)
Non-carriers (n=2,501)
BRCA2
damaging_missense
(Δ = +1.7 yrs, p=0.00397, q=0.0238)
*
Carriers (n=9)
Non-carriers (n=2,504)
MSH6
damaging_missense
(Δ = +2.9 yrs, p=0.487, q=0.637)
Carriers (n=18)
Non-carriers (n=2,495)
BRCA2
pLoF_damaging_missense
(Δ = +2.8 yrs, p=0.236, q=0.637)
Carriers (n=28)
Non-carriers (n=2,486)
MSH6
pLoF
(Δ = +0.9 yrs, p=0.866, q=0.866)
n<5
Carriers (n<5)
Non-carriers (n=2,509)
BRCA2
pLoF
(Δ = +2.2 yrs, p=0.454, q=0.637)
Carriers (n=20)
Non-carriers (n=2,494)

Fig S5. Forest plots of significant sex-stratified meta-analysis of gene burden for HCC risk for *BRCA2* and *MSH6* in ALL populations*.* Forest plots of significant sex-stratified meta-analysis of gene burden for HCC Risk for (A) *MSH6* and (B) *BRCA2* pLoF+damaging missense burden associations shown at the indicated minor allele frequency (MAF) threshold. Squares indicate per-biobank odds ratio (OR) estimates with 95% confidence intervals from burden tests. Diamonds represent the pooled random-effects meta-analysis estimate (DerSimonian-Laird) for females (pink) and males (blue) separately. Horizontal lines extending beyond the plot range are shown as arrows. * FDR q < 0.05 (Benjamini-Hochberg correction across all gene-annotation-MAF-sex groups tested). Full results for all six genes, stratified by variant category (pLoF, damaging missense, pLoF+damaging missense) are available in Table S16.

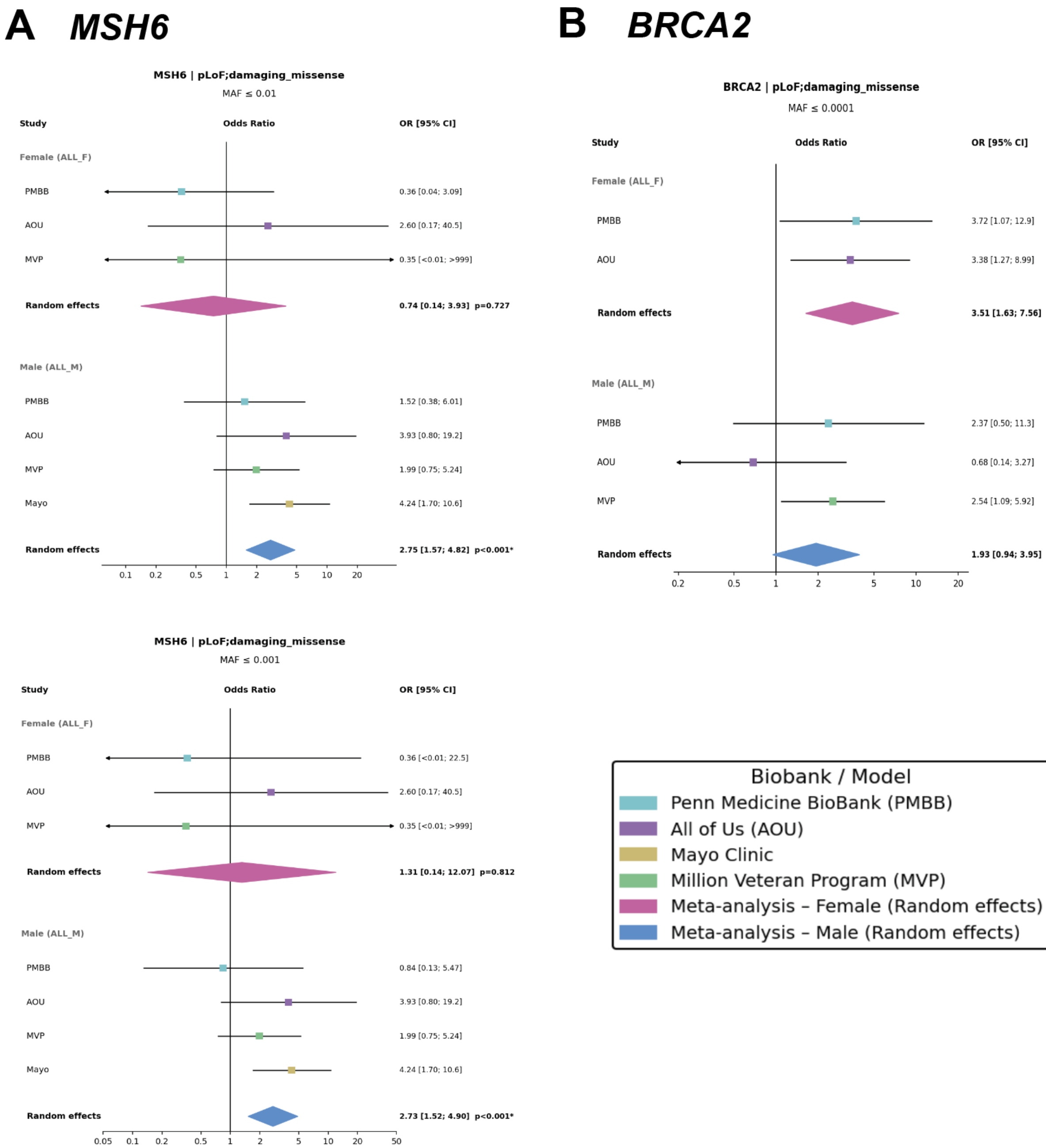
A
MSH6
MSH6 | pLoF;damaging_missense
MAF ≤ 0.01
Study
Odds Ratio
OR [95% CI]
Female (ALL_F)
PMBB
0.36 [0.04; 3.09]
AOU
2.60 [0.17; 40.5]
MVP
0.35 [<0.01; >999]
Random effects
0.74 [0.14; 3.93] p=0.727
Male (ALL_M)
PMBB
1.52 [0.38; 6.01]
AOU
3.93 [0.80; 19.2]
MVP
1.99 [0.75; 5.24]
Mayo
4.24 [1.70; 10.6]
Random effects
2.75 [1.57; 4.82] p<0.001*
0.1 0.2 0.5 1 2 5 10 20
MSH6 | pLoF;damaging_missense
MAF ≤ 0.001
Study
Odds Ratio
OR [95% CI]
Female (ALL_F)
PMBB
0.36 [<0.01; 22.5]
AOU
2.60 [0.17; 40.5]
MVP
0.35 [<0.01; >999]
Random effects
1.31 [0.14; 12.07] p=0.812
Male (ALL_M)
PMBB
0.84 [0.13; 5.47]
AOU
3.93 [0.80; 19.2]
MVP
1.99 [0.75; 5.24]
Mayo
4.24 [1.70; 10.6]
Random effects
2.73 [1.52; 4.90] p<0.001*
0.05 0.1 0.2 0.5 1 2 5 10 20 50
B
BRCA2
BRCA2 | pLoF;damaging_missense
MAF ≤ 0.0001
Study
Odds Ratio
OR [95% CI]
Female (ALL_F)
PMBB
3.72 [1.07; 12.9]
AOU
3.38 [1.27; 8.99]
Random effects
3.51 [1.63; 7.56] p=0.001*
Male (ALL_M)
PMBB
2.37 [0.50; 11.3]
AOU
0.68 [0.14; 3.27]
MVP
2.54 [1.09; 5.92]
Random effects
1.93 [0.94; 3.95] p=0.072
0.2 0.5 1 2 5 10 20
Biobank / Model
Penn Medicine BioBank (PMBB)
All of Us (AOU)
Mayo Clinic
Million Veteran Program (MVP)
Meta-analysis – Female (Random effects)
Meta-analysis – Male (Random effects)

Fig S6. Principal component analysis of genetic ancestry across contributing biobanks. Principal component analysis (PCA) plots for each contributing biobank: (A) Penn Medicine BioBank, (B) All of Us, (C) Mayo Clinic, and (D) Million Veteran Program. Individuals are colored by genetically inferred ancestry group (European [EUR], African [AFR], Admixed American [AMR], East Asian [EAS], South Asian [SAS], and Middle Eastern [MID]) and shaped by case-control status. Ancestry clusters were defined using cohort-specific principal components derived with reference panels (1000 Genomes Project, and additionally HGDP for All of Us). These plots illustrate the distribution of hepatocellular carcinoma cases and controls across ancestry groups within each cohort and reflect the underlying demographic composition of the biobanks.

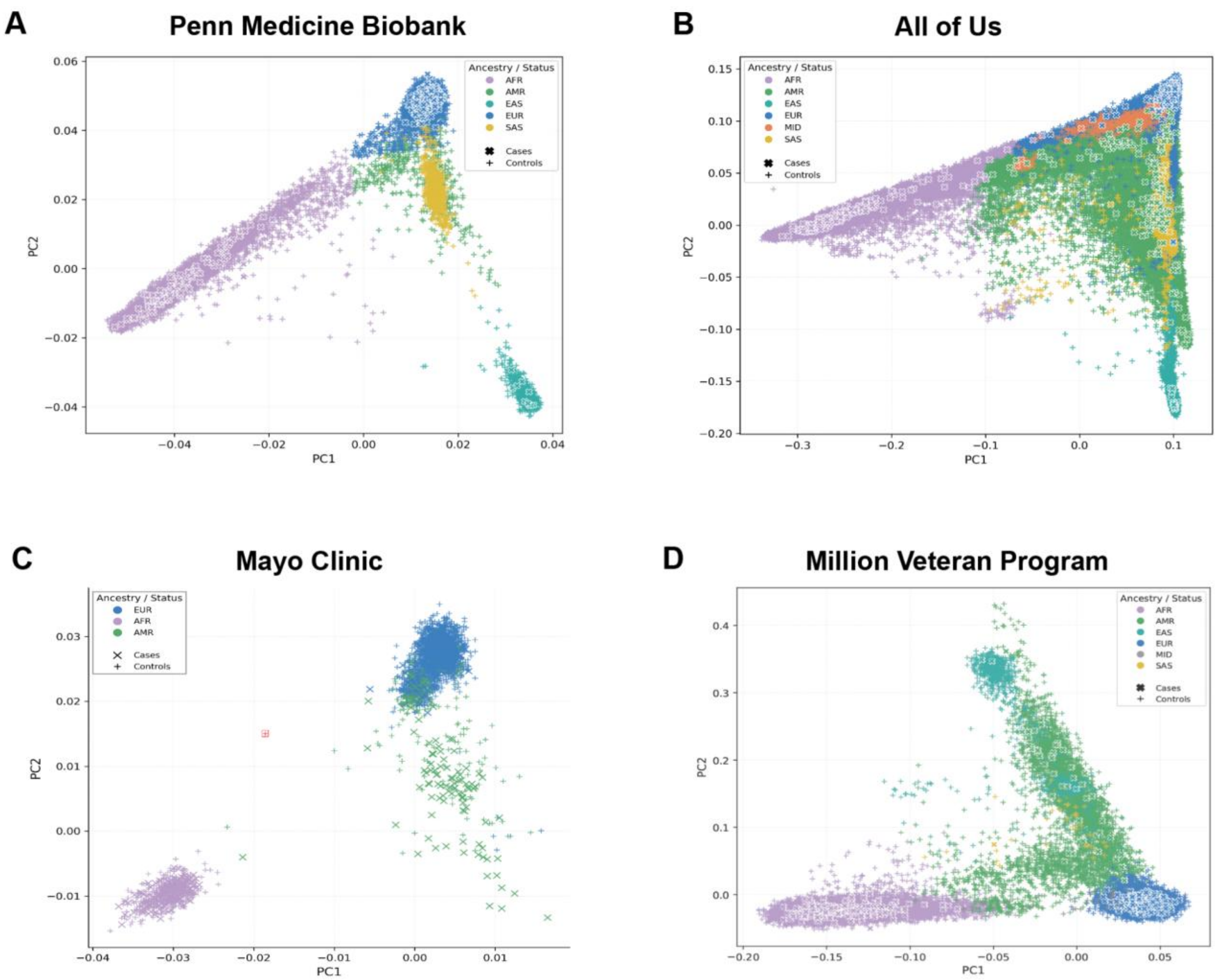

Fig S7. Summary of variant counts by biobank for ClinVar sensitivity analysis showing pLoF and damaging missense classifications. Bar plots showing the number of variants in *MSH6* and *PMS2* that meet inclusion criteria at each filtering step, stratified by biobank (PMBB, AOU, Mayo Clinic, MVP; colors shown in legend) and functional annotation category (pLoF and damaging missense). Left panels show predicted loss-of-function (pLoF) variant counts; right panels show damaging missense variant counts. The "Total" bar represents final variant counts after applying all filtering criteria described in Table S13.

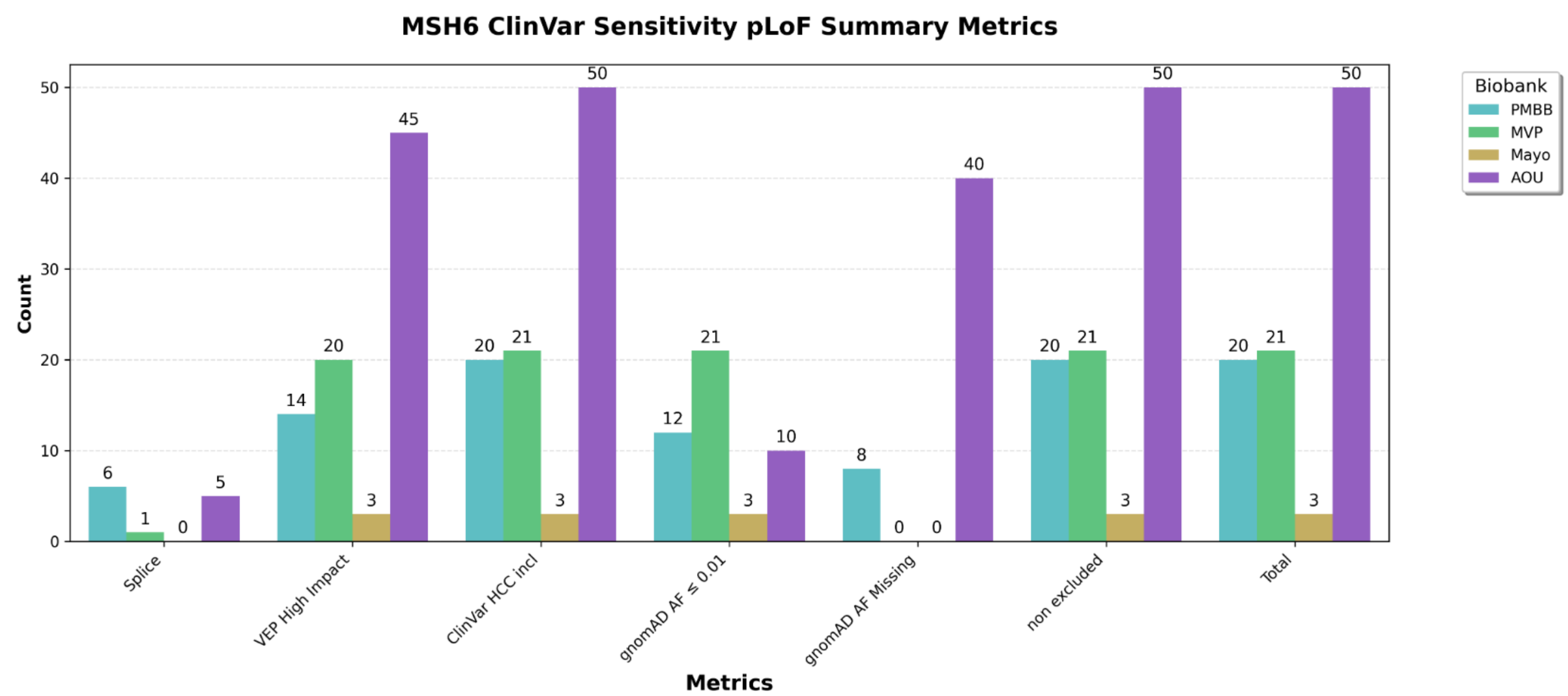

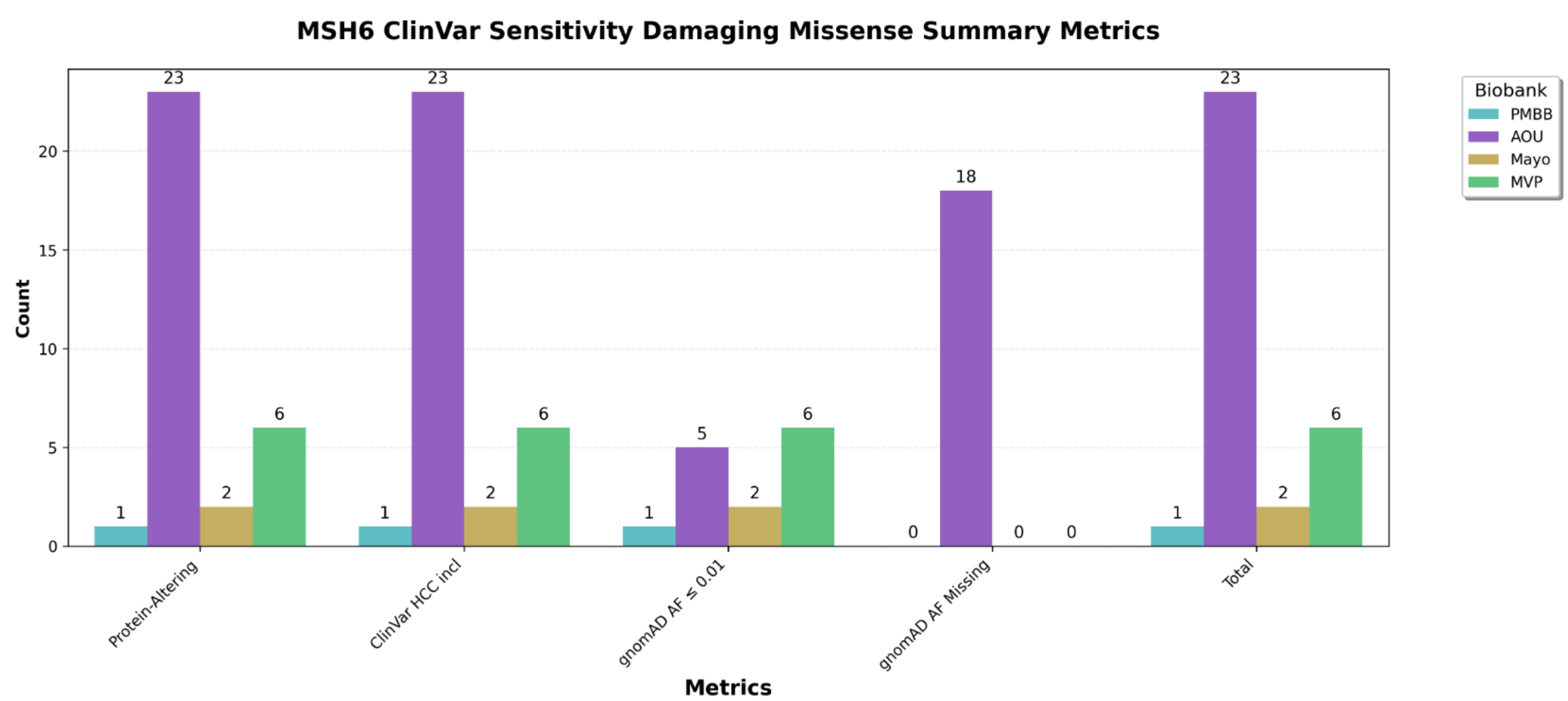

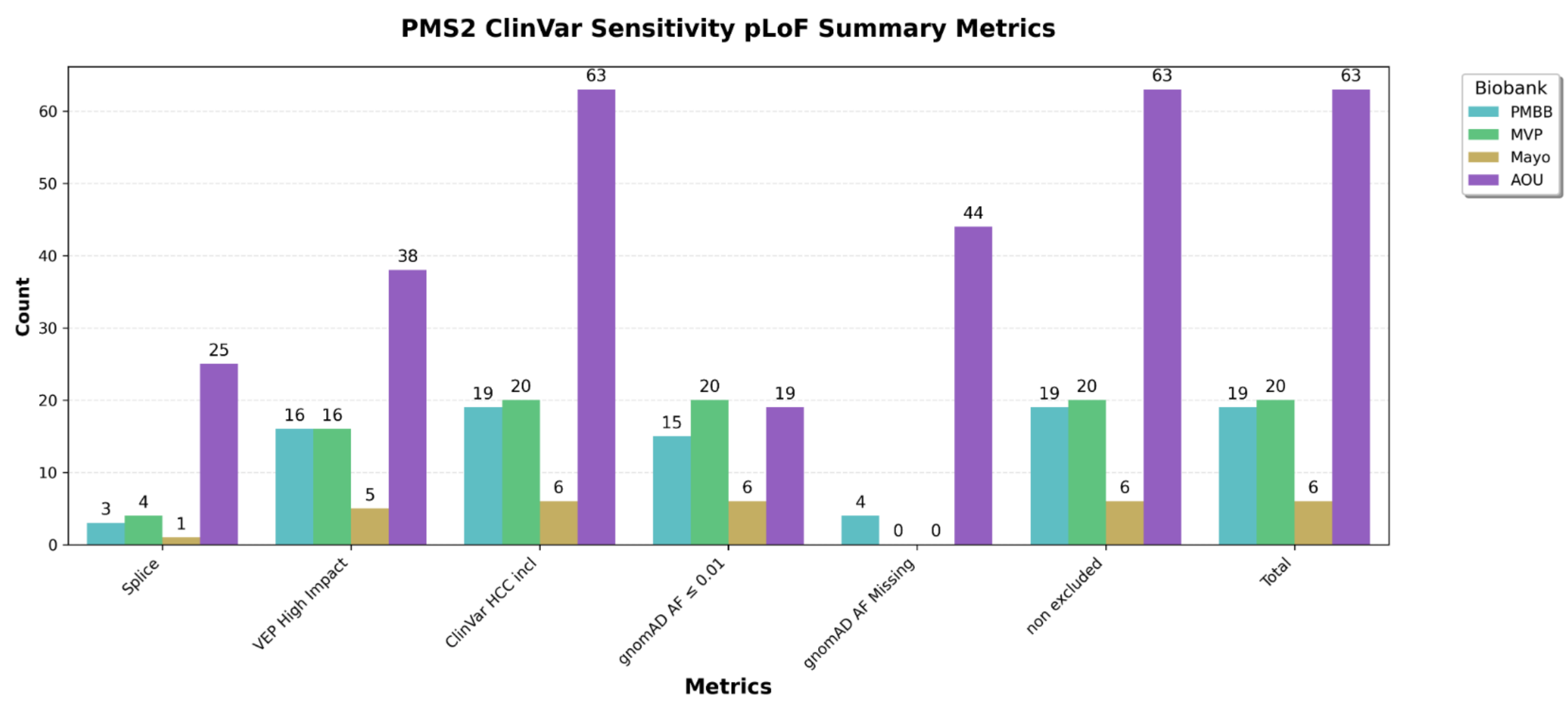
PMS2 ClinVar Sensitivity pLoF Summary Metrics
Biobank
PMBB
MVP
Mayo
AOU
Count
Metrics
Splice
VEP High Impact
ClinVar HCC incl
gnomAD AF ≤ 0.01
gnomAD AF Missing
non excluded
Total
3
4
1
25
16
16
5
38
19
20
6
63
15
20
6
19
4
0
0
44
19
20
6
63
19
20
6
63

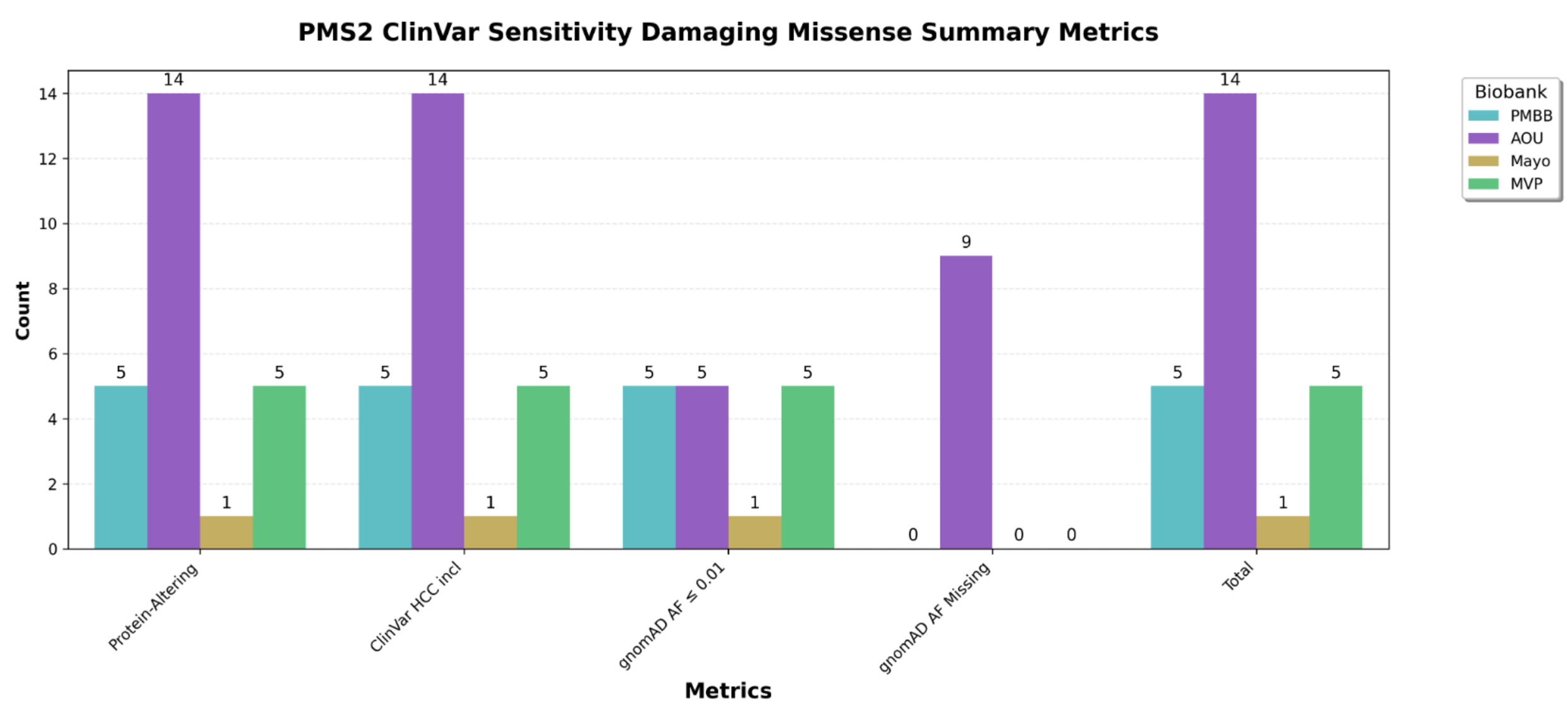
PMS2 ClinVar Sensitivity Damaging Missense Summary Metrics
Biobank
PMBB
AOU
Mayo
MVP
Count
Metrics
Protein-Altering
ClinVar HCC incl
gnomAD AF ≤ 0.01
gnomAD AF Missing
Total
5
14
1
5
5
14
1
5
5
5
1
5
0
9
0
0
5
14
1
5

Acknowledgements

Penn Medicine BioBank

PMBB Leadership Team

Daniel J. Rader, M.D.

Contribution: All authors contributed to securing funding, study design and oversight. All authors reviewed the final version of the manuscript.

Patient Recruitment and Regulatory Oversight

JoEllen Weaver, Nawar Naseer, Ph.D., M.P.H., Giorgio Sirugo, M.D., P.h.D., Afiya Poindexter, Jenna Dever, Aidan Harvey, Sydney Linn, Naman Srivastava

Contributions: JW manages patient recruitment and regulatory oversight of study. NN manages participant engagement, assists with regulatory oversight, and researcher access. GS assists with researcher access. AP, JD, AH, SL, and NS perform recruitment and enrollment of study participants.

Lab Operations

JoEllen Weaver, Meghan Livingstone, Fred Vadivieso, Stephanie DerOhannessian, Teo Tran, Julia Stephanowski, Salma Santos, Ned Haubein, P.h.D., Joseph Dunn

Contribution: JW, ML, FV, SD conduct oversight of lab operations. ML, FV, AK, SD, TT, JS, SS perform sample processing. NH, JD are responsible for sample tracking and the laboratory information management system.

Clinical Informatics

Anurag Verma, Ph.D., Colleen Morse Kripke, M.S. DPT, MSA, Marjorie Risman, M.S., Renae Judy, B.S., Colin Wollack, M.S.

Contribution: All authors contributed to the development and validation of clinical phenotypes used to identify study subjects and (when applicable) controls.

Genome Informatics

Anurag Verma Ph.D., Shefali S. Verma, Ph.D., Scott Damrauer, M.D., Yuki Bradford, M.S., Scott Dudek, M.S., Theodore Drivas, M.D., Ph.D., Zachary Rodriguez, Ph.D,

Contribution: AV, SSV, and SD are responsible for the analysis, design, and infrastructure needed to quality control genotype and exome data. YB performs

the analysis. TD and AV provides variant and gene annotations and their functional interpretation of variants.

VA Million Veteran Program |Core Acknowledgements for Publications

MVP Program Office
- Sumitra Muralidhar, Ph.D., Program Director

US Department of Veterans Affairs, 810 Vermont Avenue NW, Washington, DC 20420
- Jennifer Moser, Ph.D., Associate Director, Scientific Programs

US Department of Veterans Affairs, 810 Vermont Avenue NW, Washington, DC 20420
- Jennifer E. Deen, B.S., Associate Director, Cohort & Public Relations

US Department of Veterans Affairs, 810 Vermont Avenue NW, Washington, DC 20420

MVP Steering Committee
- Co-Chair: Philip S. Tsao, Ph.D.
VA Palo Alto Health Care System, 3801 Miranda Avenue, Palo Alto, CA 94304
- Co-Chair: Sumitra Muralidhar, Ph.D.
US Department of Veterans Affairs, 810 Vermont Avenue NW, Washington, DC 20420
- J. Michael Gaziano, M.D., M.P.H.
VA Boston Healthcare System, 150 S. Huntington Avenue, Boston, MA 02130
- Adriana Hung, M.D., M.P.H.,

VA Tennessee Valley Healthcare System, 1310 24th Avenue, South Nashville, TN 37212
- Dave Oslin, M.D.
Philadelphia VA Medical Center, 3900 Woodland Avenue, Philadelphia, PA 19104
- Deepak Voora, M.D.
Durham VA Medical Center, 508 Fulton Street, Durham, NC 27705

MVP Co-Principal Investigators
- J. Michael Gaziano, M.D., M.P.H.

VA Boston Healthcare System, 150 S. Huntington Avenue, Boston, MA 02130
- Philip S. Tsao, Ph.D.

VA Palo Alto Health Care System, 3801 Miranda Avenue, Palo Alto, CA 94304

MVP Core Operations

- Jessica V. Brewer, M.P.H., Director, MVP Cohort Operations

VA Boston Healthcare System, 150 S. Huntington Avenue, Boston, MA 02130
- Mary T. Brophy M.D., M.P.H., Director, VA Central Biorepository

VA Boston Healthcare System, 150 S. Huntington Avenue, Boston, MA 02130
- Kelly Cho, M.P.H, Ph.D., Director, MVP Phenomics

VA Boston Healthcare System, 150 S. Huntington Avenue, Boston, MA 02130
- Lori Churby, B.S., Director, MVP Regulatory Affairs

VA Palo Alto Health Care System, 3801 Miranda Avenue, Palo Alto, CA 94304
- Jacob T. Kean, Ph.D., Acting Director, VA Informatics and Computing Infrastructure (VINCI)

MVP Core Acknowledgements for Publications_October 2025
VA Salt Lake City Health Care System, 500 Foothill Drive, Salt Lake City, UT 84148
- Saiju Pyarajan Ph.D., Director, Data and Computational Sciences

VA Boston Healthcare System, 150 S. Huntington Avenue, Boston, MA 02130
- Robert Ringer, Pharm.D., Director, VA Albuquerque Central Biorepository

New Mexico VA Health Care System, 1501 San Pedro Drive SE, Albuquerque, NM 87108
- Luis E. Selva, Ph.D., Director, MVP Biorepository Coordination

VA Boston Healthcare System, 150 S. Huntington Avenue, Boston, MA 02130
- Shahpoor (Alex) Shayan, M.S., Director, MVP PRE Informatics

VA Boston Healthcare System, 150 S. Huntington Avenue, Boston, MA 02130
- Brady Stephens, M.S., Principal Investigator, MVP Information Center

Canandaigua VA Medical Center, 400 Fort Hill Avenue, Canandaigua, NY 14424
- Stacey B. Whitbourne, Ph.D., Director, MVP Cohort Development and Management

VA Boston Healthcare System, 150 S. Huntington Avenue, Boston, MA 02130

Supplementary references

1. Marrero, J. A. et al. Diagnosis, Staging, and Management of Hepatocellular Carcinoma: 2018 Practice Guidance by the American Association for the Study of Liver Diseases. Hepatology 68, 723 (2018).
2. Verma, A. et al. The Penn Medicine BioBank: Towards a Genomics-Enabled Learning Healthcare System to Accelerate Precision Medicine in a Diverse Population. Journal of Personalized Medicine 12, 1974 (2022).
3. Li, H. & Durbin, R. Fast and accurate short read alignment with Burrows-Wheeler transform. Bioinformatics 25, 1754–1760 (2009).
4. Krasheninina, O. et al. Open-source mapping and variant calling for large-scale NGS data from original base-quality scores. 2020.12.15.356360 Preprint at https://doi.org/10.1101/2020.12.15.356360 (2020).
5. Lin, M. F. et al. GLnexus: joint variant calling for large cohort sequencing. 343970 Preprint at https://doi.org/10.1101/343970 (2018).
6. Chang, C. C. et al. Second-generation PLINK: rising to the challenge of larger and richer datasets. Gigascience 4, 7 (2015).
7. Bick, A. G. et al. Genomic data in the All of Us Research Program. Nature 627, 340–346 (2024).
8. nep63. nep63/AllofUs. (2025).
9. Jawaid, T. et al. Characterization of the Cystic Phenotype Associated with Monoallelic ALG8 and ALG9 Pathogenic Variants. J Am Soc Nephrol 36, 1056–1071 (2025).
10. Verma-Lab. HCC_Analysis_Plan/variant_group_summary.py at main · Verma-

Lab/HCC_Analysis_Plan. GitHub https://github.com/Verma-Lab/HCC_Analysis_Plan/blob/main/variant_group_summary.py.
