# Supplementary material for: Multi-ancestry sequencing analysis in 293,141 participants identifies predisposition DNA-repair genes associated with HCC risk
Source: JHEP Rep. 2026 Aug 29;8(12):102019. doi: 10.1016/j.jhepr.2026.102019 (PMC13755941; doi:10.1016/j.jhepr.2026.102019)
Supplement: Supplementary file 3 [file mmc5.pdf]

JHEP|Reports

Multi-ancestry sequencing analysis in 293,141 participants identifies predisposition DNA-repair genes associated with HCC risk

Authors

Alexis M. Garofalo Perapa Chotiprasidhi, Josephine Princy Johnson, …, Marijana Vujković, Anurag Verma, Kirk J. Wangensteen

Correspondence

anurag.verma@pennmedicine.upenn.edu (A. Verma).

Graphical abstract

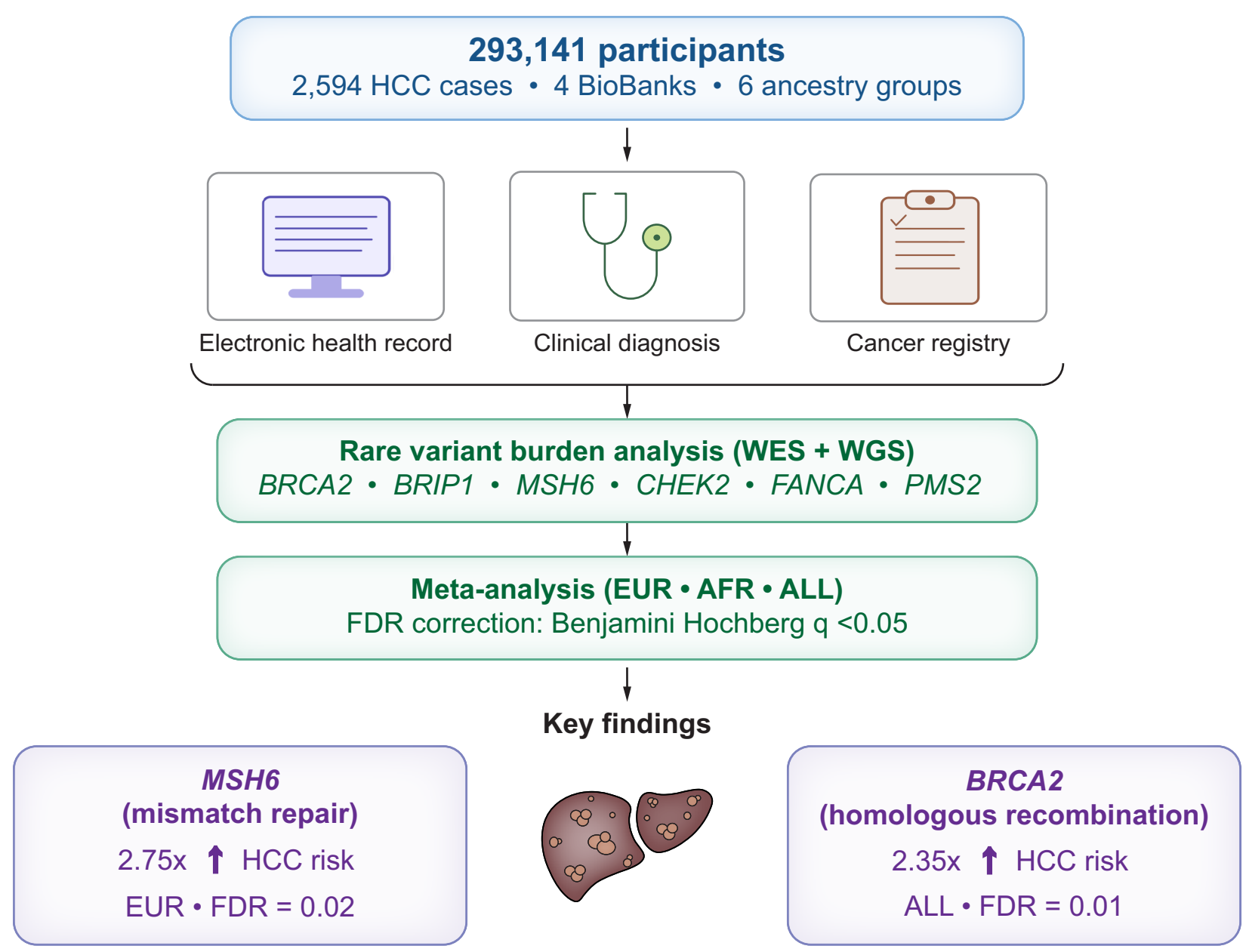

Highlights:

- Rare variants in *MSH6* and *BRCA2* increase hepatocellular carcinoma risk two-to threefold.
- *MSH6* and *BRCA2* variants linked to liver cancer risk across diverse ancestries.
- *BRCA2* liver cancer risk persists after adjusting for liver disease comorbidities.
- *BRCA2* risk is stronger in females; *MSH6* association is driven primarily by males.

Impact and implications:

Rare variants in *MSH6* and *BRCA2* genes are associated with 2.3 to 2.8-fold higher HCC risk. These findings may warrant further evaluation of liver cancer risk in individuals with *MSH6*-associated Lynch syndrome or *BRCA2*-associated hereditary cancer syndromes.

https://doi.org/10.1016/j.jhepr.2026.102019

© 2026 The Authors. Published by Elsevier B.V. on behalf of European Association for the Study of the Liver (EASL). This is an open access article under the CC BY license (http://creativecommons.org/licenses/by/4.0/). JHEP Reports, 2026, 8, 1–6

Multi-ancestry sequencing analysis in 293,141 participants identifies predisposition DNA-repair genes associated with HCC risk

Alexis M. Garofalo[1,†], Perapa Chotiprasidhi[2,†], Josephine Princy Johnson[1,3], Karina Sato-Espinoza[2], Jun Ma[2], Hunter Miller[2], Daniel O'Brien[2], Lindsay Guare[4], Katie M. Cardone[5], Nicole Palmiero[5], Zachary Rodriguez[1,4], David E. Kaplan[3,6], Julie A. Lynch[7,8], Philip S. Tsao[9,10], Daniel J. Rader[1], Lewis R. Roberts[2], Jose D. Debes[11], Emuobor Odeghe[12], Olufunmilayo Lesi[12], Ganiyat Oyeleke[12], Ângelo Z. Mattos[13], Marco Arrese[14], Enrique Carrera[15,16], Jhon Prieto[17], Andre Boonstra[18], Javier Diaz-Ferrer[19], Edith N. Okeke[20], Jun Wang[21], Lifang Hou[21], Adwoa Agyei-Nkansah[22], Mary Yeboah Afihene[23], Yaw Asante Awuku[24], Albert Nyanga[25], Kyong-Mi Chang[3,6], Samuel O. Antwi[26], Marijana Vujković[1,3,27], Anurag Verma[1,3,*,‡], Kirk J. Wangensteen[2,‡]

JHEP Reports **2026**. vol. 8 | 1–6

Check for updates

Background & Aims: Genetic testing for Lynch and *BRCA1/2*-associated hereditary cancer syndromes is recommended in patients with colon or pancreatic cancer, but their association with hepatocellular carcinoma (HCC) risk is unknown. We evaluated associations between rare germline variants in DNA-repair genes and HCC risk across ancestrally diverse cohorts.

Methods: We analyzed whole exome and whole genome sequencing data from 2,594 HCC cases and 290,547 cancer-free controls from diverse biobanks and cohorts: Penn Medicine BioBank, All of Us, Mayo Clinic, ESCALON, and the Million Veteran Program. Participants were classified into six population groups. We focused on six DNA-repair genes previously implicated in HCC: *BRCA2*, *BRIP1*, *MSH6*, *PMS2*, *CHEK2*, and *FANCA*. Gene-level burden analyses of rare predicted loss-of-function (pLoF) and damaging missense variants were performed in European and African populations, and across all six ancestry groups.

Results: In the European population, *MSH6*, a Lynch syndrome-associated gene, had the strongest association with HCC, with a 2.75-fold increased HCC risk at 1% minor allele frequency (MAF) (odds ratio [OR] = 2.75 [1.50, 5.04], p = 0.001, false discovery rate [FDR] q = 0.02), while *PMS2*, showed a nominally significant association at the same MAF threshold (OR = 1.90 [1.08, 3.34], p = 0.03, FDR q = 0.09). Combined analysis across all populations strengthened the *MSH6* finding (OR = 2.53 [1.43, 4.49], p = 0.001, FDR q = 0.01) at an MAF of 0.1%. A significant *BRCA2* association was also observed in the combined analysis at an MAF of 0.1% (OR = 2.26 [1.39, 3.67], p = 0.001, FDR q = 0.01).

Conclusions: Rare variants in *MSH6* and *BRCA2* are significantly associated with increased HCC risk, revealing a previously unconfirmed role for DNA-repair genes in HCC susceptibility across ancestrally diverse populations. These findings may inform genetic risk stratification and surveillance strategies.

© 2026 The Authors. Published by Elsevier B.V. on behalf of European Association for the Study of the Liver (EASL). This is an open access article under the CC BY license (http://creativecommons.org/licenses/by/4.0/).

Introduction

Hepatocellular carcinoma (HCC) is linked to environmental risk factors, but a family history of liver cancer increases HCC risk two-to fourfold, regardless of those factors, suggesting a strong hereditary component.[1,2] Genome-wide association studies (GWAS) have identified about a dozen common variants associated with HCC.[3,4] While common variants explain a modest proportion of HCC heritability (10–30%), rare variants with larger effect sizes may account for the remainder.[5] Recent large-scale studies in Asian biobank populations reported a nominal *BRCA2* liver cancer association and found no clear association for *MSH6*.[6,7] We conducted a case-control association analysis of rare variants in six DNA-repair genes previously identified in HCC cohort studies,[8,9] using diverse biobanks with sequencing and electronic health record data.

Materials and methods

Study design and participants

This study included participants from several diverse biobanks: Penn Medicine BioBank (PMBB), All of Us Research Program (AOU), Mayo Clinic, and Million Veteran Program (MVP).[10–12] At the Mayo Clinic the samples were collected from internal and external biobanks and cohort studies including ESCALON[13] (Table S1). HCC cases were identified using standardized algorithms based on International Classification of Diseases

* Corresponding author. Address: 3800 Hamilton Walk, A202 Richards Laboratory, Philadelphia, PA 19104, USA. Tel.: +518-605-6631.
E-mail address: anurag.verma@pennmedicine.upenn.edu (A. Verma).
† These authors contributed equally to this work.
‡ These authors jointly supervised this work.
https://doi.org/10.1016/j.jhepr.2026.102019

(ICD)-9/10 diagnosis codes (PMBB, AOU), AASLD guidelines (Mayo Clinic),[14] and cancer registry data (MVP). Cancer-free controls were individuals without cancer diagnosis codes (MVP, PMBB, AOU) or cancer diagnosis (Mayo Clinic). Specific diagnostic codes, inclusion/exclusion criteria, and baseline liver disease characteristics are provided (Table S2 and S12).

Participants were classified into six population groups based on genetic similarity to the 1000 Genomes Project and, for AOU, additionally the Human Genome Diversity Project: European (EUR), African (AFR), Admixed American (AMR), East Asian (EAS), South Asian (SAS), and Middle Eastern (MID) (Fig. S6). We performed separate analyses for EUR and AFR, and a combined multi-population analysis (ALL) that includes all six population groups.

Genomic sequencing and quality control

Genomic DNA extracted from participants' blood or saliva samples were used for whole exome sequencing (WES) or whole exome sequencing (WGS). Detailed information on the sequencing protocols, variant calling workflows and quality control metrics for each biobank is provided in the Supplementary Methods.

Variant annotation and classification

We developed a standardized workflow for consistent variant annotation across biobanks. Variants were functionally annotated using Ensembl Variant Effect Predictor (VEP) and filtered to rare variants (minor allele frequency [MAF] ≤1% or missing in the Genome Aggregation Database [gnomAD]) with matched annotation from NCBI and EBI (MANE) transcripts.[15–17] We classified variants into three functional categories: predicted loss-of-function (pLoF), damaging missense, and combined pLoF + damaging missense variants. The pLoF tier was the highest level of confidence of pathogenic/likely pathogenic status according to ClinVar and/or predicted impact (Table S3–S5). A complete list of rare variants included in the six-gene-burden analysis across all four biobanks is provided (Table S6).

Statistical analysis

For each biobank, we performed gene-burden association tests using the Scalable and Accurate Implementation of Generalized mixed model (SAIGE-GENE+) for EUR, AFR, and ALL populations.[18] Models were adjusted for age, sex, and genetic ancestry principal components. We tested three maximum MAF thresholds (1%, 0.1%, and 0.01%) to evaluate the contribution of rare to ultra-rare variants.

Next, we conducted fixed-effects inverse variance weighted meta-analysis for EUR and AFR populations, with random-effects inverse variance weighted meta-analysis for ALL populations using the metafor R package (R Foundation for Statistical Computing, Vienna, Austria). Statistical significance was determined by false discovery rate (FDR) correction (Benjamini–Hochberg, q <0.05) across all genes, variant bins, and MAF thresholds.

Results

Study population and variant distribution

We analyzed genetic sequencing data on 293,141 participants across several diverse biobanks, including 2,594 HCC cases and 290,547 cancer-free controls. HCC cases were predominantly male and older than controls (Table 1).

Variant carriers were identified for each gene in all biobanks, with counts varying by gene and biobank (Table 1; Fig. S1 and Table S10 and S11).

Rare variants in *MSH6, PMS2,* and *BRCA2* are associated with higher HCC risk

Gene-burden meta-analyses in the EUR population showed a 2.75-fold increased risk of HCC in the pLoF + damaging missense burden analysis for *MSH6*, at 1% MAF (odds ratio [OR] = 2.75 [1.50, 5.04], p = 0.004, FDR q = 0.02) (Fig. 1A and Table S7). We observed similar findings in the ALL group at 1% MAF (OR = 2.39 [1.33, 4.29], p = 0.004, FDR q = 0.02) and 0.1% MAF (OR = 2.53 [1.43, 4.49], p = 0.001, FDR q = 0.01) (Fig. 1A and Table S9). We also observed a nominally significant association for the pLoF + damaging missense burden in *PMS2* in EUR populations at 1% MAF (OR = 1.90 [1.08, 3.34], p = 0.03, FDR q = 0.09) (Fig. 1B and Table S7). In the ALL group, *BRCA2* pLoF burden was significantly associated with increased risk of HCC at both 1% and 0.1% MAF thresholds (OR = 2.35 [1.39, 3.60], p = 0.001, FDR q = 0.01) and (OR = 2.26 [1.39, 3.67], p = 0.001, FDR q = 0.01) (Fig. 1C and Table S9). In the AFR population, a nominally significant association for the pLoF + damaging missense burden in *BRCA2* at 0.01% MAF was not significant after multiple testing correction (OR = 3.28 [0.99, 10.92], p = 0.05, FDR q = 0.26) (Fig. 1C and Table S8). A sensitivity analysis requiring ClinVar pathogenic/likely pathogenic annotation for both pLoF and damaging missense categories was not feasible for *MSH6* or *PMS2*, as too few HCC cases carried qualifying variants in these genes (Table S13 and Fig. S7).

The rare variant association in *BRCA2* is independent of liver disease comorbidities

Carriers of *BRCA2* and *MSH6* rare variants were not enriched for specific liver disease comorbidities among HCC cases (Table S14 and Fig. S3). Comorbidity-adjusted burden analyses confirmed *BRCA2* pLoF remained significant in the ALL group at the 1% MAF threshold (OR = 2.37 [1.42, 3.97], p = 0.001, FDR q = 0.02), 0.1% MAF (OR = 2.35 [1.39, 3.98], p = 0.001, FDR q = 0.02), and 0.01% MAF (OR = 2.88 [1.59, 5.20], $p = 4.56 \times 10^{-4}$, FDR q = 0.02) (Table S15 and Fig. S2).

Sex-stratified associations and co-occurring cancer enrichment in variant carriers

Sex-stratified burden analyses revealed significant associations for *BRCA2* pLoF + damaging missense in females (ALL_F: OR = 3.51 [1.63, 7.56], p = 0.001, FDR q = 0.05) and *MSH6* pLoF + damaging missense in males (ALL_M: OR = 2.75 [1.57, 4.82], $p = 3.98 \times 10^{-4}$, FDR q = 0.04) at MAF thresholds of 0.01% and 1%, respectively (Table S16 and Fig. S5). Age at HCC diagnosis did not differ significantly between carriers and non-carriers of *BRCA2* or *MSH6* variants across cohorts (Fig. S4). Among HCC cases, carriers of damaging missense variants showed significant enrichment for co-occurring cancers compared to non-carriers: *MSH6* carriers were enriched for breast cancer (OR = 22.6 [2.93, 174.7], p = 0.003, FDR q = 0.033) and ovarian cancer (OR = 24.5 [3.18, 189.8], p = 0.002,

Table 1. Baseline characteristics of hepatocellular carcinoma cases and cancer-free controls by study site.

Metric	PMBB	AOU	Mayo	MVP
Demographics, % male				
HCC cases	354 (72.10)	483 (54.58)	459 (71.50)	569 (98.78)
Cancer-free controls	13,422 (47.23)	76,895 (37.01)	1,903 (65.15)	47,735 (92.79)
Demographics, % female				
HCC cases	137 (27.90)	402 (45.42)	183 (28.50)	7 (1.22)
Cancer-free controls	14,999 (52.77)	130,866 (62.99)	1,018 (34.85)	3,709 (7.21)
Age				
HCC cases	60.44 ± 10.99	63.30 ± 10.60	59.2 ±13.63	63.5 ± 6.8
Cancer-free controls	50.35 ± 16.99	49.50 ± 16.64	62.04 ± 12.83	62.5 ± 13.9
Total N (case | control)				
HCC cases | cancer-free controls	491 | 28,421	885 | 207,761	642 | 2,921	576 | 51,444
Ancestry subgroups (case | control)				
EUR: HCC cases | cancer-free controls	352 | 19,002	515 | 111,262	314 | 2,162	326 | 32,406
AFR: HCC cases | cancer-free controls	121 | 7,518	166 | 45,261	192 | 468	191 | 14,316
AMR: HCC cases | cancer-free controls	5 | 457	177 | 43,040	120 | 214	54 | 3,938
EAS: HCC cases | cancer-free controls	10 | 582	15 | 4,539	0 | 0	4 | 683
SAS: HCC cases | cancer-free controls	0 | 524	7 | 2,825	0 | 0	1 | 33
MID: HCC cases | cancer-free controls	0 | 0	5 | 834	0 | 0	0 | 68
Total carrier counts case + control (npLOF | ndamag | npLOF + damaging)*				
BRCA2	287 | 93 | 380	934 | 538 | 1,469	9 | 0 | 9	277 | 120 | 397
FANCA	116 | 40 | 156	771 | 286 | 1,055	20 | 36 | 56	437 | 74 | 511
MSH6	180 | 10 | 190	233 | 44 | 277	5 | 18 | 23	82 | 299 | 381
BRIP1	56 | 95 | 151	398 | 802 | 1,200	6 | 9 | 14	93 | 96 | 189
CHEK2	328 | 99 | 427	2,077 | 505 | 2,582	44 | 8 | 52	539 | 33 | 572
PMS2	283 | 218 | 501	1,831 | 1,513 | 3,344	11 | 36 | 45	938 | 230 | 1,168

Baseline characteristics of study participants across biobanks. Data are presented as n (%) for categorical variables and mean ± standard deviation for continuous variables. *Carrier counts are shown for gene annotations at an MAF of 0.01 only. Carrier counts presented as npLOF | ndamaging | npLOF + damaging for each gene. AFR, African; AMR, Admixed American; EAS, East Asian; EUR, European; HCC, hepatocellular carcinoma; MAF, minor allele frequency; MID, Middle Eastern; SAS, South Asian.

FDR q = 0.031), and *FANCA* carriers were enriched for prostate cancer (OR = 4.66 [1.61, 13.49], p = 0.005, FDR q = 0.046). *CHEK2* results were excluded because of insufficient carrier counts (Table S17).

Discussion

Our study provides evidence that rare variants in *MSH6* and *BRCA2* are associated with increased HCC risk across ancestrally diverse populations, supporting a role for these genes in liver cancer susceptibility. These associations are biologically plausible given the roles of *MSH6* and *PMS2* in mismatch repair and *BRCA2* in homologous recombination, pathways whose disruption increases cancer susceptibility.

Analyses of Asian populations by other groups identified a nominal *BRCA2*-liver cancer signal that did not reach stringent significance thresholds and no *MSH6* association.[6,7] In contrast, our analysis across diverse biobanks represents a comprehensive and ancestrally diverse investigation of rare germline HCC susceptibility.

The *BRCA2* pLoF association remained significant after adjusting for major liver disease comorbidities (HBV, HCV, alcohol-related liver disease, and metabolic dysfunction-associated steatohepatitis/metabolic dysfunction-associated steatotic liver disease), suggesting the risk does not depend on the type of environmental factor. Sex-stratified analyses revealed that the pLoF + damaging missense association for *BRCA2* was stronger in females and the *MSH6* association was driven primarily by males, highlighting possible sex-related heterogeneity in genetic susceptibility to HCC. Among HCC cases, *MSH6* damaging missense carriers showed enrichment for breast and ovarian cancers, consistent with known *MSH6* biology, while *BRCA2* carriers showed no such enrichment. These results should be interpreted with caution given the limited number of participants with variants in these genes. Together, these findings have potential clinical implications. While genetic testing is already recommended for patients with pancreatic and colon cancers,[19,20] its role in HCC requires further study. Carrier status for variants in these genes may inform HCC surveillance strategies. While HCC risk in the general population is relatively low, underlying liver disease increases this risk by more than 40-fold.[21] Lynch syndrome or *BRCA2* carrier status could further increase this risk. Whether Lynch syndrome or *BRCA2* carrier status should inform treatment selection with immune checkpoint inhibitors or poly ADP-ribose polymerase (PARP) inhibitors in HCC warrants further investigation.[22,23] Patients with HCC who also have Lynch syndrome or *BRCA2* variants are at heightened risk for developing additional primary cancers in other organs, making comprehensive surveillance important.

Several limitations of our study should be acknowledged. First, variant carriers were not present for all six genes across all biobanks, limiting meta-analyses. Second, although we used a multi-tiered approach for high-confidence pLoF variant classification, only *BRCA2* achieved statistical significance for pLoF variants alone. *MSH6* and *PMS2* associations required inclusion of damaging missense variants, indicating lower confidence in the specific variant classes driving these associations. A ClinVar-restricted sensitivity analysis was not feasible because of insufficient case carriers, underscoring the necessity of computational predictions for rare variant burden analyses at biobank scale. Many rare variants from populations of African and Latin American ancestry may lack ClinVar classifications due to ancestral ascertainment bias in clinical variant databases.[24,25] Third, broader analyses beyond the six genes tested here are needed to identify additional HCC susceptibility genes.

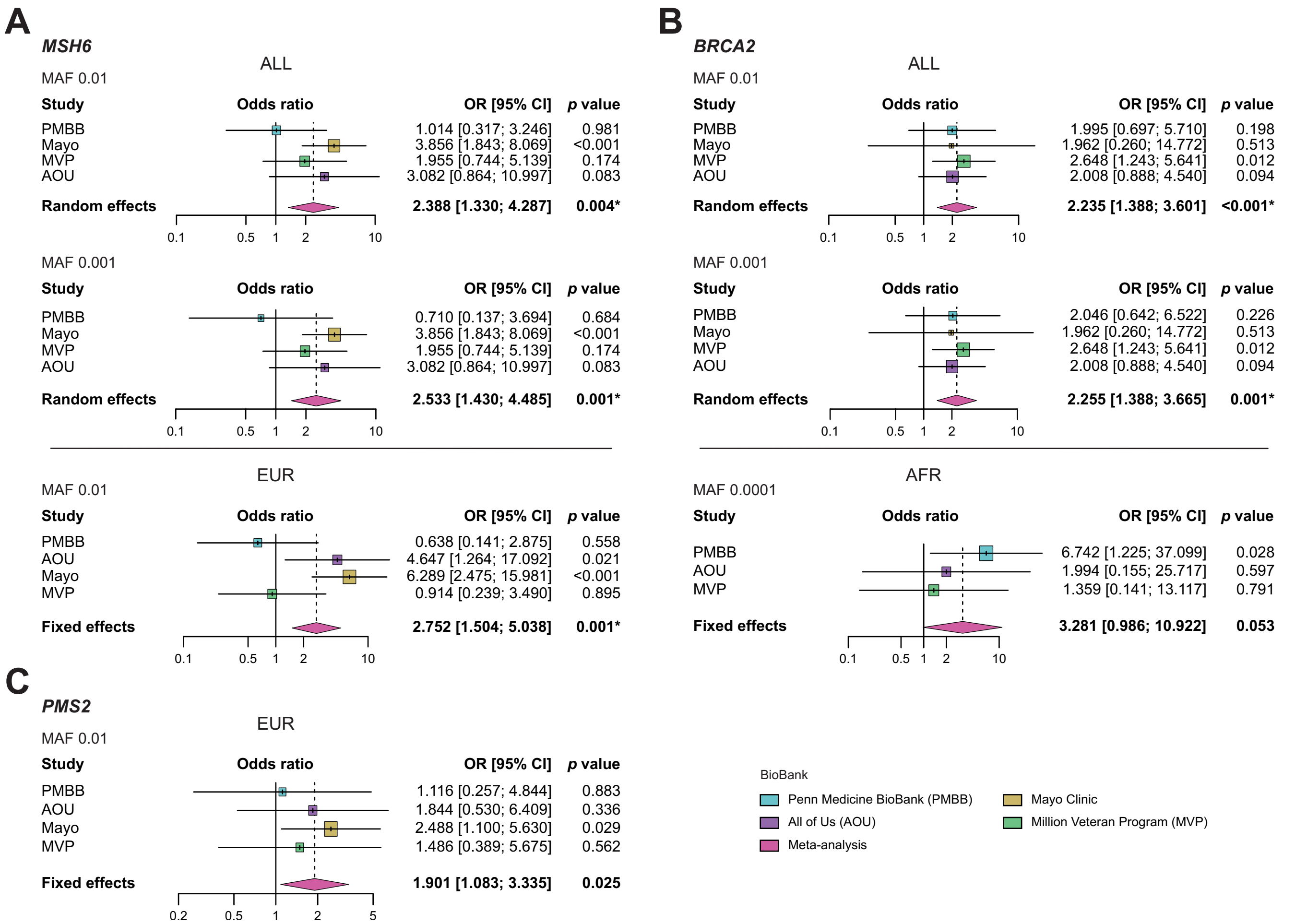

Fig. 1. Meta-analysis forest plots of six-gene-burden for HCC risk. Forest plots of odds ratios and 95% confidence intervals for gene-burden associations with HCC risk for: (A) *MSH6*; (B) *PMS2*; and (C) *BRCA2*. Results stratified by population: ALL, EUR (European), and AFR (African) with MAF thresholds (0.01, 0.001, and 0.0001). BioBank results: PMBB, AOU, Mayo, and MVP. Box size = study weight; diamonds = pooled estimates. Asterisks (*) indicate FDR <0.05 (Benjamini–Hochberg). The *p* values were from inverse variance weighted meta-analysis. Significant: *MSH6* EUR/ALL (p ≤0.004, FDR ≤0.02) and *BRCA2* ALL (p = 0.001, FDR = 0.01). Non-significant: *PMS2* EUR (p = 0.03, FDR = 0.09) and *BRCA2* AFR (p = 0.05, FDR = 0.26). ALL, all ancestry groups combined; AOU, All of Us Research Program; FDR, false discovery rate; HCC, hepatocellular carcinoma; MAF, minor allele frequency; Mayo, Mayo Clinic; MVP, Million Veteran Program.

Affiliations

[1]*Department of Medicine, Division of Translational Medicine and Human Genetics, University of Pennsylvania Perelman School of Medicine, Philadelphia, PA, USA;* [2]*Department of Medicine, Division of Gastroenterology and Hepatology, Mayo Clinic, Rochester, MN, USA;* [3]*Corporal Michael J. Crescenz VA Medical Center, Philadelphia, PA, USA;* [4]*Department of Pathology, University of Pennsylvania Perelman School of Medicine, Philadelphia, PA, USA;* [5]*Department of Genetics, University of Pennsylvania Perelman School of Medicine, Philadelphia, PA, USA;* [6]*Department of Medicine, Division of Gastroenterology and Hepatology, University of Pennsylvania Perelman School of Medicine, Philadelphia, PA, USA;* [7]*VA Informatics and Computing Infrastructure, VA Salt Lake City Health Care System, Salt Lake City, UT, USA;* [8]*Department of Internal Medicine, Division of Epidemiology, University of Utah School of Medicine, Salt Lake City, UT, USA;* [9]*VA Palo Alto Healthcare System, Palo Alto, CA, USA;* [10]*Department of Medicine, Stanford University School of Medicine, Stanford, CA, USA;* [11]*Department of Medicine, University of Minnesota, Minneapolis, MN, USA;* [12]*University of Lagos, Lagos, Nigeria;* [13]*Graduate Program in Medicine, Division of Hepatology, Federal University of Health Sciences of Porto Alegre, Porto Alegre, Brazil;* [14]*Departamento de Gastroenterología, Escuela de Medicina, Pontificia Universidad Católica de Chile, Santiago, Chile;* [15]*Department of Gastroenterology and Hepatology, Hospital Especialidades Eugenio Espejo, Quito, Ecuador;* [16]*School of Medicine, Universidad San Francisco de Quito, Quito, Ecuador;* [17]*Centro de Enfermedades Hepáticas y Digestivas, Bogotá, Colombia;* [18]*Department of Gastroenterology and Hepatology, Erasmus University Medical Center, Rotterdam, The Netherlands;* [19]*Department of Digestive Diseases, Division of Hepatology, Hospital Nacional Edgardo Rebagliati Martins, Lima, Peru;* [20]*Jos University Teaching Hospital, Jos, Nigeria;* [21]*Department of Preventive Medicine, Division of Cancer Epidemiology and Prevention, Northwestern University, Chicago, IL, USA;* [22]*Korle Bu Teaching Hospital, Accra, Ghana;* [23]*Kwame Nkrumah University of Science and Technology, Kumasi, Ghana;* [24]*University of Health and Allied Sciences, Ho, Ghana;* [25]*Mbingo Baptist Hospital, Mbingo, Cameroon;* [26]*Department of Quantitative Health Sciences, Division of Epidemiology, Mayo Clinic, Jacksonville, FL, USA;* [27]*Department of Epidemiology, University of Pennsylvania Perelman School of Medicine, Philadelphia, PA, USA*

Abbreviations

AFR, African; ALL, all ancestry groups combined; AMR, Admixed American; AOU, All of Us Research Program; EAS, East Asian; EUR, European; FDR, false discovery rate; gnomAD, Genome Aggregation Database; GWAS, genome-wide association study; HCC, hepatocellular carcinoma; MAF, minor allele frequency; MANE, Matched Annotation from NCBI and EMBL-EBI; MID, Middle Eastern; MVP, Million Veteran Program; pLoF, predicted loss-of-function; PMBB, Penn Medicine BioBank; SAIGE-GENE+, Scalable and Accurate Implementation of Generalized mixed model; SAS, South Asian; WES, whole exome sequencing; WGS, whole genome sequencing.

Financial support

NIH R37CA259201, NIH R37CA297814, NIH K01CA237875, Mayo Clinic Office of Research Diversity and Inclusion, National Cancer Institute (NCI) through the Mayo Clinic Hepatobiliary SPORE (P50 CA210964), National Cancer Institute. Grant Number: NIH/NCI U54CA221205, Swedish Research Council, and International Collaboration Grant ID: dnr 2015–05928.

Conflicts of interest

JAL reports grants from Alnylam Pharmaceuticals, Inc., AstraZeneca Pharmaceuticals LP, Biodesix, Inc, Janssen Pharmaceuticals, Inc., Novartis International AG, Parexel International Corporation through the University of Utah or Western Institute for Veteran Research outside the submitted work. The remaining authors declare no conflicts of interest that pertain to this work.

Please refer to the accompanying ICMJE disclosure forms for further details.

Authors' contributions

Conceptualization: AV KJW; Methodology: AMG, PC, JPJ, KS, JM, HM, DO, AV, KJW; Investigation: AMG, PC, JPJ, KSE, JM, HM, DO, LG, KMC, NP, ZR; Visualization: AMG, AV ; Funding acquisition: KJW, AV,JD,LR,KMC; Project administration: KJW, AV; Supervision: KJW, AV, MV; Writing – original draft: AMG, PC, JPJ, JM, AV, KJW; Writing – review & editing: HM, DEK, JAL, PST, DJR, LRR, JDD, EO, OL, GO, AZM, MA, EC, JP, AB, JDF, ENO, JW, LH, AAN, MYA, YAA, AN, KMC, SOA

Data availability

Data supporting the findings of this study are derived from four biobank resources, none of which are publicly available because of participant privacy and confidentiality protections. All of Us Research Program Controlled Tier Dataset v8 data are accessible to authorized researchers through the All of Us Researcher Workbench (https://allofus.nih.gov/article/opportunities-researchers). Million Veteran Program (MVP) data access is restricted to VA investigators on approved federally funded projects; requests can be made to the MVP Program Office. Mayo Clinic Biobank data are available to authorized researchers in collaboration with a Mayo Clinic investigator; requests can be directed to Biobank@mayo.edu. Penn Medicine BioBank (PMBB) data may be available upon reasonable request and with PMBB permission. Analysis code is deposited in the GitHub repository: https://github.com/Verma-Lab/hcc-rare-variant-analysis.

Acknowledgements

We acknowledge the Penn Medicine BioBank (PMBB) for providing data and thank the patient- participants of Penn Medicine who consented to participate in this research program. We would also like to thank the Penn Medicine BioBank team and Regeneron Genetics Center for providing genetic variant data for analysis. The PMBB is approved under IRB protocol# 813913 and supported by Perelman School of Medicine at University of Pennsylvania, a gift from the Smilow family, and the National Center for Advancing Translational Sciences of the National Institutes of Health under CTSA award number UL1TR001878. The authors thank the Million Veteran Program (MVP) staff, researchers, and volunteers, who have contributed to MVP, and especially who previously served their country in the military and now generously agreed to enroll in the study (see mvp.va.gov for more information). The underlying work was based on data from the Million Veteran Program,Office of Research and Development, Veterans Heath Administration, and was supported by the Veterans Administration MVP award #000, VA Merit Award #I01-BX003362 (Chang/Tsao) and the Department of Veterans Affairs (VA) Informatics and Computing Infrastructure (VINCI), including data analytics conducted by its Precision Medicine research team, which is funded under the research priority to Put VA Data to Work for Veterans (VA ORD 24-D4V-02). This publication does not represent the views of the Department of Veterans Affairs or the United States Government. We gratefully acknowledge All of Us participants for their contributions, without whom this research would not have been possible. We also thank the National Institutes of Health's All of Us Research Program for making available the participant data examined in this study.

Supplementary data

Supplementary data to this article can be found online at https://doi.org/10.1016/j.jhepr.2026.102019.

References

Author names in bold designate shared co-first authorship

[1] Turati F, Edefonti V, Talamini R, et al. Family history of liver cancer and hepatocellular carcinoma. Hepatology 2012;55:1416–1425.
[2] Hassan MM, Spitz MR, Thomas MB, et al. The association of family history of liver cancer with hepatocellular carcinoma: a case-control study in the United States. J Hepatol 2009;50. 334–1.
[3] Hassan MM, Li D, Han Y, et al. Genome-wide association study identifies high-impact susceptibility loci for HCC in North America. Hepatology 2024;80:87–101.
[4] **Ghouse J**, **Gellert-Kristensen H**, O'Rourke CJ, et al. Genome-wide meta-analysis identifies nine loci associated with higher risk of hepatocellular carcinoma development. JHEP Rep 2025;7:101485.
[5] **Dai J**, **Shen W**, **Wen W**, et al. Estimation of heritability for nine common cancers using data from genome-wide association studies in Chinese population. Int J Cancer 2017;140:329–336.
[6] Mizukami K, Usui Y, Iwasaki Y, et al. Pan-cancer prevalence, risk, and clinical and demographic characteristics of Lynch Syndrome-associated variants in BioBank Japan. Commun Med 2025;5:522.
[7] Momozawa Y, Sasai R, Usui Y, et al. Expansion of cancer risk Profile for BRCA1 and BRCA2 pathogenic variants. JAMA Oncol 2022;8:871–878.
[8] **Chotiprasidhi P**, **Sato-Espinoza AK**, Wangensteen KJ. Germline genetic associations for Hepatobiliary cancers. Cell Mol Gastroenterol Hepatol 2024;17:623–638.
[9] Mezina A, Philips N, Bogus Z, et al. Multigene panel testing in individuals with hepatocellular carcinoma identifies pathogenic germline variants. JCO Precis Oncol 2021;5. PO.21.00079.
[10] Verma A, Damrauer SM, Naseer N, et al. The Penn Medicine BioBank: towards a genomics-enabled learning healthcare system to accelerate precision medicine in a diverse population. J Pers Med 2022;12:1974.
[11] Bick AG, Metcalf GA, Mayo KR, et al. Genomic data in the all of Us research Program. Nature 2024;627:340–346.
[12] **Gaziano JM**, **Concato J**, Brophy M, et al. Million Veteran Program: a mega-biobank to study genetic influences on health and disease. J Clin Epidemiol 2016;70:214–223.
[13] Ayoub A, Anugwom CM, Prieto J, et al. Assessment of STAT4 variants and risk of hepatocellular carcinoma in Latin Americans and Europeans. Cancers (Basel) 2023;15:4530.
[14] Marrero JA, Kulik LM, Sirlin CB, et al. Diagnosis, staging, and management of hepatocellular carcinoma: 2018 practice guidance by the American Association for the Study of Liver Diseases. Hepatology 2018;68:723–750.
[15] McLaren W, Gil L, Hunt SE, et al. The Ensembl variant effect predictor. Genome Biol 2016;17:122.
[16] Karczewski KJ, Francioli LC, Tiao G, et al. The mutational constraint spectrum quantified from variation in 141,456 humans. Nature 2020;581:434–443.
[17] Morales J, Pujar S, Loveland JE, et al. A joint NCBI and EMBL-EBI transcript set for clinical genomics and research. Nature 2022;604:310–315.
[18] Zhou W, Bi W, Zhao Z, et al. SAIGE-GENE+ improves the efficiency and accuracy of set-based rare variant association tests. Nat Genet 2022;54:1466–1469.
[19] Stoffel EM, McKernin SE, Brand R, et al. Evaluating susceptibility to pancreatic cancer: ASCO Provisional clinical Opinion. J Clin Oncol 2019;37:153–164.
[20] Tung N, Ricker C, Messersmith H, et al. Selection of germline genetic testing panels in patients with cancer: ASCO Guideline. J Clin Oncol 2024;42:2599–2615.
[21] Tarao K, Nozaki A, Ikeda T, et al. Real impact of liver cirrhosis on the development of hepatocellular carcinoma in various liver diseases-meta-analytic assessment. Cancer Med 2019;8:1054–1065.

[22] **Williams MH**, **Hadjinicolaou AV**, **Norton BC**, et al. Lynch syndrome: from detection to treatment. Front Oncol 2023;13:1166238.
[23] Ragupathi A, Singh M, Perez AM, et al. Targeting the BRCA1/2 deficient cancer with PARP inhibitors: clinical outcomes and mechanistic insights. Front Cel Dev Biol 2023;11:1133472.
[24] Kessler MD, Yerges-Armstrong L, Taub MA, et al. Challenges and disparities in the application of personalized genomic medicine to populations with African ancestry. Nat Commun 2016;7:12521.
[25] Venner E, Patterson K, Kalra D, et al. The frequency of pathogenic variation in the All of Us cohort reveals ancestry-driven disparities. Commun Biol 2024;7:174.

Keywords: Liver cancer; Rare variants; Clinical genetics; Genetic association study; Biobanks; Electronic health records; Precision medicine; Cancer genetics; Lynch syndrome; *BRCA2*; *MSH6*.
Received 12 December 2025; received in revised form 20 August 2026; accepted 26 August 2026; Available online 29 August 2026

Journal of Hepatology, Volume *8*

Supplemental information

Multi-ancestry sequencing analysis in 293,141 participants identifies predisposition DNA-repair genes associated with HCC risk

Alexis M. Garofalo, Perapa Chotiprasidhi, Josephine Princy Johnson, Karina Sato-Espinoza, Jun Ma, Hunter Miller, Daniel O'Brien, Lindsay Guare, Katie M. Cardone, Nicole Palmiero, Zachary Rodriguez, David E. Kaplan, Julie A. Lynch, Philip S. Tsao, Daniel J. Rader, Lewis R. Roberts, Jose D. Debes, Emuobor Odeghe, Olufunmilayo Lesi, Ganiyat Oyeleke, Ângelo Z. Mattos, Marco Arrese, Enrique Carrera, Jhon Prieto, Andre Boonstra, Javier Diaz-Ferrer, Edith N. Okeke, Jun Wang, Lifang Hou, Adwoa Agyei-Nkansah, Mary Yeboah Afihene, Yaw Asante Awuku, Albert Nyanga, Kyong-Mi Chang, Samuel O. Antwi, Marijana Vujković, Anurag Verma, and Kirk J. Wangensteen

Multi-ancestry sequencing analysis in 293,141 participants identifies predisposition DNA repair genes associated with HCC risk

Alexis M. Garofalo, Perapa Chotiprasidhi, Josephine Princy Johnson, Karina Sato-Espinoza, Jun Ma, Hunter Miller, Daniel O'Brien, Lindsay Guare, Katie M. Cardone, Nicole Palmiero, Zachary Rodriguez, David E. Kaplan, Julie A. Lynch, Philip S. Tsao, Daniel J. Rader, Lewis R. Roberts, Jose D. Debes, Emuobor Odeghe, Funmilayo Lesi, Ganiyat Oyeleke, Ângelo Z. Mattos, Marco Arrese, Enrique Carrera, Jhon Prieto, Andre Boonstra, Javier Diaz-Ferrer, Edith N. Okeke, Jun Wang, Lifang Hou, Adwoa Agyei-Nkansah, Mary Yeboah Afihene, Yaw Asante Awuku, Albert Nyanga, Kyong-Mi Chang, Samuel O. Antwi, Marijana Vujković, Anurag Verma*, Kirk Joseph Wangensteen*

Table of contents

Materials and methods…………………………………………………………………...2
Supplementary table legends…………………………………......………………..….7
Fig S1……………………………………………………………………………………...11
Fig. S2……………………………………………………………………………..……...17
Fig. S3……………………………………………………………………………..…....18
Fig. S4…………………………………………………………………………………......20
Fig. S5……………………………………………………………………………………..22
Fig. S6……………………………………………………………………………………..24
Fig. S7……………………………………………………………………………..…....25
Acknowledgements…………………………………………………………………….....27
Supplementary references ……………………………………….……………………….31

Materials and methods

Phenotype definitions

For this multi-center sequencing-based association analysis, we developed harmonized definitions of HCC cases and cancer-free controls that were adapted to the available data sources across participating biobanks. In MVP, HCC cases were identified using cancer registry data, while cancer-free controls were defined based on the absence of any malignant neoplasm ICD-9/10 diagnosis codes. In Mayo Clinic, cases were defined as individuals diagnosed with HCC based on criteria consistent with the American Association for the Study of Liver Diseases (AASLD) guidelines[1], and controls were individuals without any clinical evidence of cancer. Details about the Mayo Clinic sample collection from biobanks around the world are provided in Supplementary Table S1.

In PMBB and AOU, where cancer registry was not available, we identified HCC cases using a standardized algorithm based on the presence of at least one HCC-related ICD-9 or ICD-10 diagnosis code, with cancer-free controls defined as individuals without any malignant neoplasm ICD-9 or ICD-10 codes. The specific diagnostic codes and inclusion/exclusion criteria used for case and control classification are provided in Supplementary Table S2.

In ESCALON, a prospective multi-center study across seven medical institutions in six Latin American countries (Argentina, Brazil, Chile, Colombia, Ecuador, and Peru), participants were enrolled as part of ongoing HCC biomarker surveillance and not randomly.[2] HCC cases were identified using criteria consistent with the American Association for the Study of Liver Diseases (AASLD) guidelines, based on either histological confirmation via liver biopsy or characteristic imaging findings. For imaging-based diagnosis, multiphasic computed tomography (CT) or magnetic resonance imaging (MRI) demonstrating arterial phase hyperenhancement with venous phase washout was required. Liver biopsy was reserved for cases with inconclusive imaging results or when additional histopathological characterization was clinically indicated.

Germline genetic sequencing

Genomic DNA from participants underwent different sequencing approaches across biobanks. In PMBB, whole exome sequencing (WES) was performed on 57,170 participants across two batches using different capture products. The first batch utilized a modified version of IDT's xGen probe library while the second batch used the Twist Comprehensive Exome panel, sample collections details are outlined elsewhere.[3] Sequencing and variant calling were conducted by the Regeneron Genetics Center

(RGC) using the Twist Comprehensive Exome panel and the Illumina NovaSeq 6000 platform. FASTQ files were aligned to GRCh38 using BWA-MEM[4] using alt-aware mapping following the original quality functional equivalent (OQFE) protocol[5]. Variant calling was performed using a Parabricks-accelerated version of DeepVariant v0.10 with a custom WES model. Individual sample variant calls were generated in genomic VCF (gVCF) format and aggregated using GLnexus v1.4.3[6] with the DeepVariantWES configuration to create a joint genotyped project level VCF (pVCF). The final dataset was converted to PLINK bed/bim/fam format using PLINK v1.9[7] for downstream analyses.

In AOU, short-read whole genome sequencing (WGS) was performed on more than 414,000 participants as part of the curated data repository (CDR) version 8, sample collection details, sequencing, and variant calling details are provided here.[8] Additional variant normalization and annotation preprocessing specific to AOU data were performed as detailed in the project repository.[9]

In Mayo Clinic, whole-exome sequencing (WES) was performed using two platforms: the Regeneron “Project Generation” platform and, for Tapestry program participants, the Helix Exome+ platform. ESCALON samples were also sequenced using the Helix Exome+ platform. For samples sequenced by Regeneron, sequencing and quality control procedures have been previously described in supplementary methods.[10] Sample-level QC filtering removed samples with gender discordance, exome coverage below 80% at 20X depth, contamination exceeding 5%, or identified as duplicates.

For samples sequenced by Helix, the Exome+ assay is an augmented exome capture that additionally includes capture regions outside the exome for purposes of capturing common tag SNPs. This is intended to provide a genome-wide “backbone” of interrogated genetic variation to emulate traditional genome-wide genotyping array data typically used for genome-wide association studies. The Helix Pipeline follows the GATK best practices for variant processing with the BWA-MEM aligner. It achieves a mean SNP and indel call rate of ≥99.5% for approximately 600 genes associated with hereditary cancer, cardiovascular disease, pharmacogenomics, and carrier screening; ≥98% for roughly 3,000 genes linked to single-gene disorders; and ≥96% for the whole exome.

In MVP, deep whole-genome sequencing (WGS) was performed on 109,826 participants. Sequence reads were aligned to GRCh38 using BWA-MEM v0.7.15, alignments were compressed to CRAM. Variant calling was performed with GATK 4.1.0.0 and variant calls were generated in gVCF format. Sample-level QC filtering removed samples with per-base sequence quality score <28, GC content outside 40%-41%, <95% properly mapped reads, contamination exceeding 5%, or mean sequencing depth <18x.

Genetic Ancestry Classification

Genetic ancestry was determined using cohort-specific pre-computed principal components (PCs), all derived using the 1000 Genomes Project (1KG) as a reference panel. For All of Us, ancestry classification incorporated both 1KG and the Human Genome Diversity Project (HGDP) as reference panels. Participants were classified into six ancestry groups (European [EUR], African [AFR], Admixed American [AMR], East Asian [EAS], South Asian [SAS], and Middle Eastern [MID]) based on genetic similarity to reference population clusters in PC space. To visualize ancestry distributions, HCC cases and controls were overlaid onto cohort-level PCA plots, with HCC cases and cancer-free controls distinguished by marker shape (Fig. S6). Individuals with admixed backgrounds were assigned to the ancestry group with the highest genetic similarity. Primary analyses were conducted separately for EUR and AFR populations, with an additional combined multi-population analysis (ALL) inclusive of all six groups. The relatively low representation of EAS and SAS participants reflects the underlying demographic composition of the contributing biobanks.

Variant Annotation

Variants from all biobanks were functionally annotated using the Ensembl Variant Effect Predictor (VEP) v113.0 with consistent annotation criteria applied across datasets, except MVP which used v110. All analyses used the GRCh38 reference genome. The annotation pipeline incorporated multiple specialized plugins and databases to comprehensively characterize variant functional consequences.

Core annotation plugins included Loss-of-Function Transcript Effect Estimator (LoFTEE) for identifying high-confidence loss-of-function variants, Combined Annotation Dependent Depletion (CADD) for deleteriousness prediction, Rare Exome Variant Ensemble Learner (REVEL) for predicting the pathogenicity of missense variants, and SpliceAI for splice site prediction. Additional annotations were derived from ClinVar (accessed in October, 2024, Tables S4, S5). These include variants in the six genes designated as pathogenic or likely pathogenic (P/LP) by a majority of laboratories after

2015, when ACMG first released the modern guidelines for variant annotation.[11] These P/LP variants were included in an inclusion list (Table S4). Variants for which a majority of laboratories classified them as “uncertain significance” or “benign” were placed in an exclusion list (Table S5).

Following annotation, variants were classified into three functional categories using standardized criteria applied across all biobanks: (1) High Confidence Predicted Loss-of-Function (pLoF) variants, (2) damaging missense variants, and (3) combined pLoF+damaging missense variants. To ensure consistent variant grouping across the biobanks we used the Python script detailed in the project repository[12] to classify annotated variants into the functional bins.The detailed variant classification workflow is provided in Supplementary Table S3. The number of pLoF and damaging missense variants meeting inclusion criteria at each filtering step across all biobanks is provided in Supplementary Figure S2.

Only rare variants were retained, defined as those with maximum broad ancestry minor allele frequency ≤1% in gnomAD or absent from gnomAD. Variants were then categorized using MANE (Matched Annotation from NCBI and EMBL-EBI) transcripts into two main functional classes as outlined in Supplementary Table 3, with the third category representing the union of these classes.

pLoF variants included: ClinVar pathogenic or likely pathogenic variants (curated post-2015), LOFTEE high-confidence loss-of-function variants, VEP high-impact consequences (transcript_ablation, stop_gained, frameshift_variant, stop_lost, start_lost, transcript_amplification, feature_elongation, feature_truncation), and splice site variants with SpliceAI scores ≥0.2. Variants classified as ClinVar variants of uncertain significance, likely benign, or benign were excluded. The curated ClinVar inclusion criteria are detailed in Supplementary Table S4, and exclusion criteria are provided in Supplementary Table S5.

Damaging missense protein-altering variants included missense, start-loss, stop-loss, in-frame indel, or splicing variants (not already classified as high-confidence pLoF) meeting at least one of the following criteria: REVEL score ≥0.773, CADD

score ≥28.1, SpliceAI score ≥0.2, or LOFTEE low-confidence loss-of-function classification. A complete list of rare variants included in the six-gene burden analysis across all four biobanks for the ALL population group is provided in Supplementary Table S6.

Statistical analysis

Based on previous studies detecting germline variants in *FANCA*, *BRIP1*, *CHEK2*, and *BRCA2, MSH6* and *PMS2* among HCC patients across independent cohorts, we focused our rare variant burden analysis on these six genes. This approach was chosen to maximize statistical power for detecting associations while minimizing the multiple testing burden. Gene-burden association tests were performed within each biobank to assess associations between germline variants in these six genes and HCC risk. Analyses were conducted separately for European ancestry (EUR), African ancestry (AFR), and combined multi-ancestry (ALL) populations using the SAIGE-GENE+ framework. All models included age, sex, and principal components of genetic ancestry as covariates (10 PCs for MVP, AOU, and Mayo Clinic; 6 PCs for PMBB). Three maximum minor allele frequency (MAF) thresholds were tested for each gene: 1%, 0.1%, and 0.01%.

After obtaining study-level results, to increase the number of cases and controls and gain statistical power we performed fixed effects inverse variance weighted meta-analysis for EUR and AFR across the four biobanks and random effects inverse variance weighted meta-analysis for ALL. Meta-analyses were conducted using the metafor package in R. Statistical significance was determined using a False Discovery Rate (FDR) correction with the Benjamini-Hochberg method at a cutoff of 0.05. Complete association results for EUR, AFR, and ALL populations are provided in Supplementary Tables S7, S8, and S9, respectively.

Supplementary table legends

- Table S1. Mayo Clinic Site Breakdown by Population Group. Sample distribution across different data sources and sites for Mayo Clinic stratified by population groups and case-control status. Data are presented for three population groups: European (EUR), Admixed American (AMR), and African (AFR) populations. Numbers represent individual participants classified as either hepatocellular carcinoma (HCC) cases or cancer-free controls.
- Table S2. Phenotyping algorithms and criteria for hepatocellular carcinoma case and control identification across biobanks. Inclusion and exclusion criteria used for case-control classification across participating biobanks. HCC cases were identified using ICD (International Classification of Diseases) codes in PMBB and AOU, clinical diagnosis in Mayo Clinic, and cancer registry data in MVP. Cancer-free controls excluded individuals with any cancer diagnosis. Specific ICD codes for HCC inclusion and exclusion of other hepatobiliary cancers ensured accurate phenotyping across biobanks. PMBB = Penn Medicine BioBank; AOU = All of Us Research Program; MVP = Million Veteran Program.
- Table S3. Inclusion and exclusion criteria for binning variants into functional categories. Filtering criteria and functional annotation pipeline used to classify genetic variants for burden testing. Variants were filtered by frequency (MAF ≤ 1% in gnomAD) and analyzed using MANE (Matched Annotation from NCBI and EMBL-EBI) transcripts. Three functional categories were defined: High Confidence predicted Loss-of-Function (pLoF) variants identified through ClinVar pathogenic classifications, high-impact VEP consequences, or SpliceAI scores ≥ 0.2; Damaging Missense variants determined by REVEL ≥ 0.773, CADD ≥ 28.1, or SpliceAI ≥ 0.2; and a combined category including both pLoF and damaging missense variants. ClinVar variants classified as variants of uncertain significance (VUS), likely benign, or benign were excluded from high confidence pLoF categories to ensure stringent functional impact prediction.
- Table S4. Curated ClinVar Inclusion list. Curated list of ClinVar pathogenic and likely pathogenic variants used for inclusion in the pLoF classification. This list includes variants with review status annotations curated after 2015 and expert-reviewed classifications that meet ACMG criteria for pathogenic or likely pathogenic designations. These variants were prioritized for inclusion in gene burden analyses based on their established clinical significance.
- Table S5. Curated ClinVar Exclusion list. Curated list of ClinVar variants classified as variants of uncertain significance (VUS), likely benign, or benign that were excluded from pLoF classification. These variants were excluded to ensure specificity and reduce potential false positive associations from variants lacking sufficient evidence of pathogenicity.

- Table S6. Rare variants included across four biobanks for six-gene burden analysis in ALL populations. Rare variants meeting pLoF or damaging missense criteria that were tested in the gene burden analysis across Penn Medicine BioBank (PMBB), Mayo Clinic, All of Us (AOU), and Million Veteran Program (MVP). For pLoF variants, ClinVar annotations derive from the curated Pathogenic/Likely Pathogenic inclusion list (Supplementary Table S4), with "NOT_FOUND_IN_CLINVAR" indicating absence from this list. For damaging missense variants, ClinVar annotations derive from VEP annotation files, with "-" indicating no ClinVar classification. Values <5 for MAC_Case and MAC_Control are censored as "<5" for privacy protection.
- Table S7. Fixed-effects meta-analysis results for six-gene burden associations with HCC risk in EUR populations. Results from fixed-effects meta-analysis across four biobanks (PMBB, AOU, Mayo Clinic, MVP) showing gene burden associations with HCC risk in European populations. Analysis includes pLoF, damaging missense, and pLoF+damaging missense variants across six DNA repair genes at three MAF thresholds: 1%, 0.1%, and 0.01%. Results indicate FDR significance status and corresponding FDR q-values after Benjamini-Hochberg correction for multiple testing.
- Table S8. Fixed-effects meta-analysis results for six-gene burden associations with HCC risk in AFR populations. Results from fixed-effects meta-analysis across three biobanks (PMBB, AOU, MVP) showing gene burden associations with HCC risk in African populations. Analysis includes pLoF, damaging missense, and pLoF+damaging missense variants across six DNA repair genes at three MAF thresholds: 1%, 0.1%, and 0.01%. Results indicate FDR significance status and corresponding FDR q-values after Benjamini-Hochberg correction for multiple testing.
- Table S9. Random-effects meta-analysis results for six-gene burden associations with HCC risk in ALL populations. Results from random-effects meta-analysis across four biobanks (PMBB, AOU, Mayo Clinic, MVP) showing gene burden associations with HCC risk in ALL populations (including EUR, AFR, AMR, EAS, SAS, MID). Analysis includes pLoF, damaging missense, and pLoF+damaging missense variants across six DNA repair genes at three MAF thresholds: 1%, 0.1%, and 0.01%. Results indicate FDR significance status and corresponding FDR q-values after Benjamini-Hochberg correction for multiple testing.
- Table S10. Cross-biobank average of carrier frequencies of rare variant categories in ALL HCC cases and cancer-free controls. Cross-biobank average carrier frequencies of rare variant categories in ALL HCC cases and cancer-free controls across six DNA repair genes. Values represent the mean carrier frequency averaged across four biobanks (PMBB, AOU, Mayo Clinic, MVP) and are reported as case frequency | control frequency for three variant categories:

pLoF, damaging missense, and pLoF+damaging missense. Variants are restricted to those included in the SAIGE-GENE+ burden test group files.

- Table S11. Cross-biobank average of ancestry-stratified rare variant carrier frequencies in HCC cases and cancer-free controls. Cross-biobank average carrier frequencies of rare pathogenic variant categories in HCC cases and cancer-free controls, stratified by genetic ancestry (EUR, AFR, AMR, EAS, SAS, MID) across six DNA repair genes. Values represent mean carrier frequency across up to four biobanks (PMBB, AOU, Mayo Clinic, MVP), reported as case frequency | control frequency for three variant categories: pLoF, damaging missense, and pLoF+damaging missense. Variants are restricted to those included in the SAIGE-GENE+ burden test group files. When a biobank reported N/A for a given ancestry-gene-category combination, reflecting absence of that ancestry cohort rather than zero frequency, it was excluded from the average. Averages were calculated using only available data. Per-biobank frequencies are not reported to protect participant privacy. Values of 0.000 indicate no observed carriers or frequencies too small to display at three decimal places.
- Table S12. Baseline disease characteristics for HCC cases and cancer-free controls across biobanks. Baseline liver disease characteristics among HCC cases and cancer-free controls across four biobanks (PMBB, AOU, Mayo Clinic, MVP). Values are reported as number (percentage) for cases and controls for each condition, including hepatitis B virus (HBV), hepatitis C virus (HCV), alcohol-related liver disease, and MASH/MASLD. Percentages are calculated within each biobank and case-control group. A total of 93 EAS, SAS, and other participants were excluded from Mayo Clinic due to unanalyzable SAIGE results.
- Table S13. Inclusion and exclusion criteria for binning variants into functional categories for ClinVar sensitivity analysis. Variants were filtered by minor allele frequency and transcript, then classified into functional categories (high-confidence pLoF, damaging missense, and combined) using ClinVar pathogenicity and VEP annotations. This sensitivity analysis restricts to higher-confidence pathogenic variants to clarify the contribution of damaging missense variants to gene-based associations.
- Table S14. Random-effects meta-analysis of rare variant carrier enrichment for liver disease comorbidities among HCC cases in six DNA repair genes across biobanks. Carrier status was defined as carrying at least one qualifying rare variant (pLoF, damaging missense, and combined), restricted to HCC cases across PMBB, AOU, Mayo Clinic, and MVP. Liver disease comorbidities: hepatitis B virus (HBV), hepatitis C virus (HCV), alcohol-related liver disease, and MASH/MASLD. Pooled odds ratios were estimated using the DerSimonian-Laird random-effects model. Q: Cochran's Q; I^2: between-study heterogeneity; τ^2: between-study variance.

- Table S15. Random-effects meta-analysis results for *BRCA2* and *MSH6* gene burden associations with HCC risk in ALL populations after adjustment for liver disease comorbidities. Results from random-effects meta-analysis across four biobanks (PMBB, AOU, Mayo Clinic, MVP) showing *MSH6* and *BRCA2* gene burden associations with HCC risk in ALL populations (including EUR, AFR, AMR, EAS, SAS, MID). Analysis includes pLoF, damaging missense, and pLoF+damaging missense variants at three MAF thresholds: 1%, 0.1%, and 0.01%. Results indicate FDR significance status and corresponding FDR q-values after Benjamini-Hochberg correction for multiple testing.
- Table S16. Random-effects sex-stratified meta-analysis results of six-gene burden associations with HCC risk. Results from random-effects meta-analysis across four biobanks (PMBB, AOU, Mayo Clinic, MVP) showing gene burden associations with HCC risk stratified by sex (male and female) in ALL populations (including EUR, AFR, AMR, EAS, SAS, MID). Analysis includes pLoF, damaging missense, and pLoF+damaging missense variants across six DNA repair genes at three MAF thresholds: 1%, 0.1%, and 0.01%. Results indicate FDR significance status and corresponding FDR q-values after Benjamini-Hochberg correction for multiple testing.
- Table S17. Random-effects meta-analysis of co-occurring cancer enrichment among HCC cases carrying rare variants in six DNA repair genes. Within each biobank (PMBB, AOU, Mayo Clinic, MVP), Fisher's exact test was used to assess enrichment of breast, ovarian, pancreatic, or prostate cancer diagnoses among HCC cases carrying rare variants (pLoF, damaging missense, or pLoF+damaging missense; MAF ≤ 1%) compared to non-carriers. Cross-biobank pooling used inverse variance-weighted meta-analysis with a DerSimonian-Laird random-effects model. Log odds ratios and standard errors were derived from 2×2 contingency tables per biobank. Columns include: gene, annotation mask, cancer type, number of contributing biobanks, total carriers, Cochran's Q statistic, I^2, Tau^2, pooled odds ratio (RE_OR), 95% confidence interval bounds, Z-value, p-value, FDR q-value (Benjamini-Hochberg), and FDR significance.

Fig S1. Summary of variant counts by biobank showing pLoF and damaging missense classifications. Bar plots showing the number of variants in six DNA repair genes (*BRCA2*, *BRIP1*, *CHEK2*, *FANCA*, *MSH6*, *PMS2*) that meet inclusion criteria at each filtering step, stratified by biobank (PMBB, AOU, Mayo Clinic, MVP; colors shown in legend) and functional annotation category (pLoF and damaging missense). Left panels show predicted loss-of-function (pLoF) variant counts; right panels show damaging missense variant counts. The "Total" bar represents final variant counts after applying all filtering criteria described in Table S3. Results demonstrate differences in variant detection across biobanks, with whole genome sequencing biobanks (AOU, MVP) identifying more variants overall than whole exome sequencing biobanks (PMBB, Mayo Clinic).

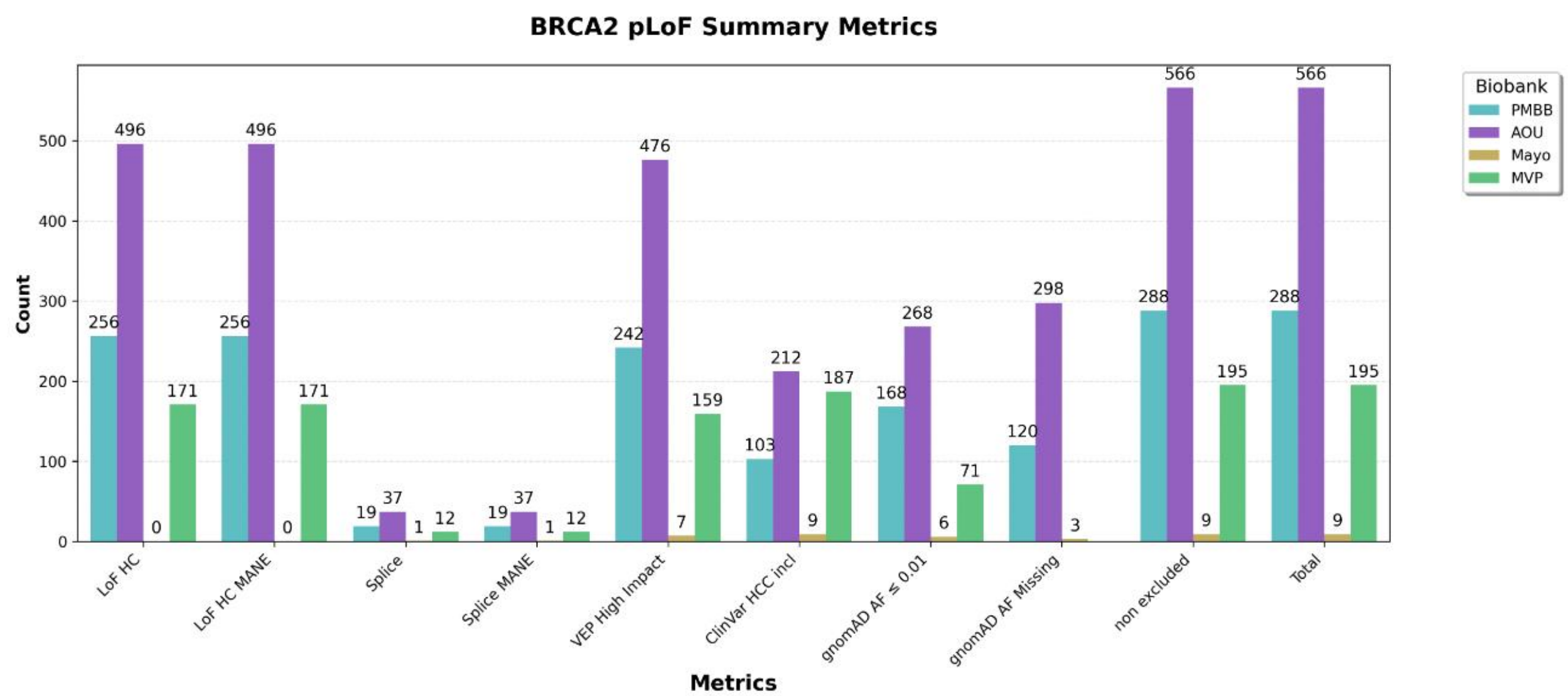

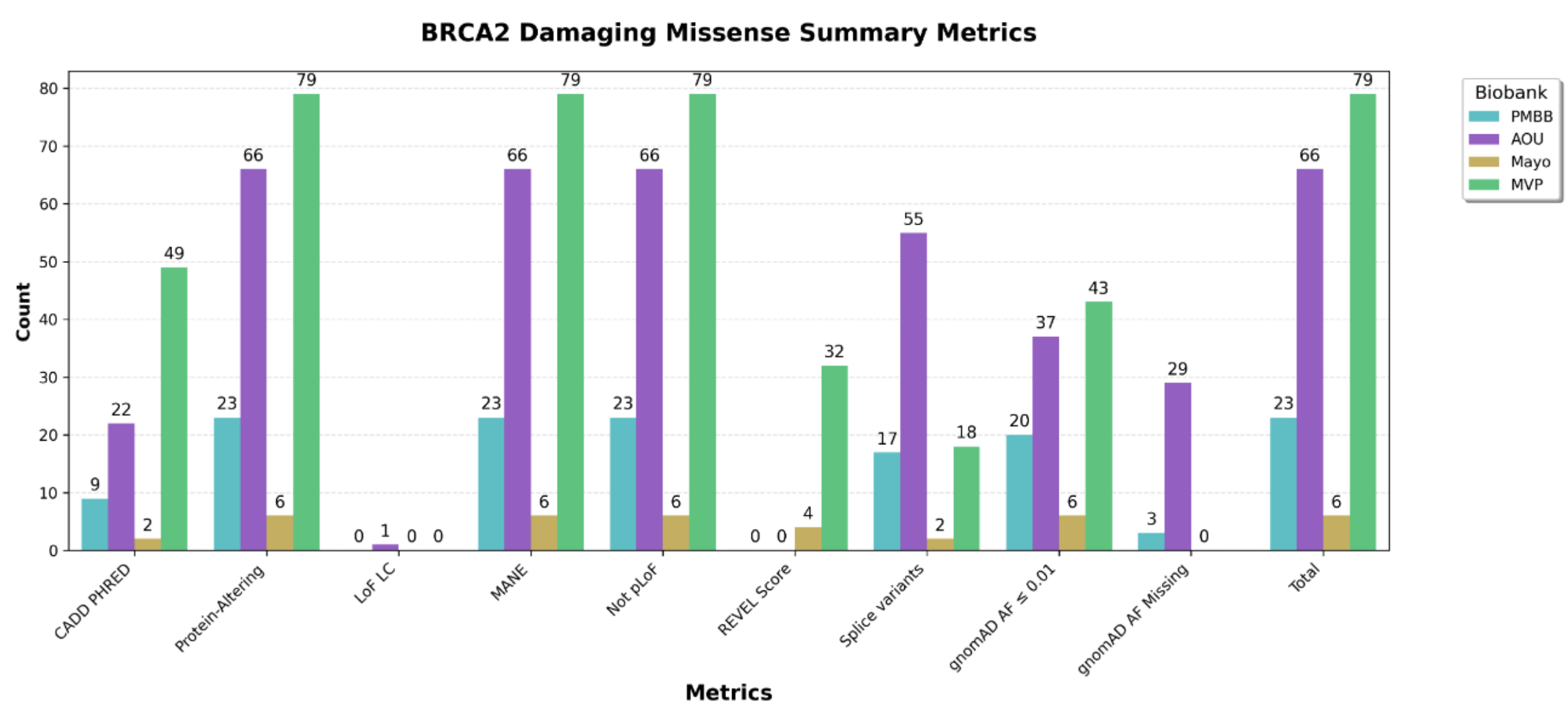

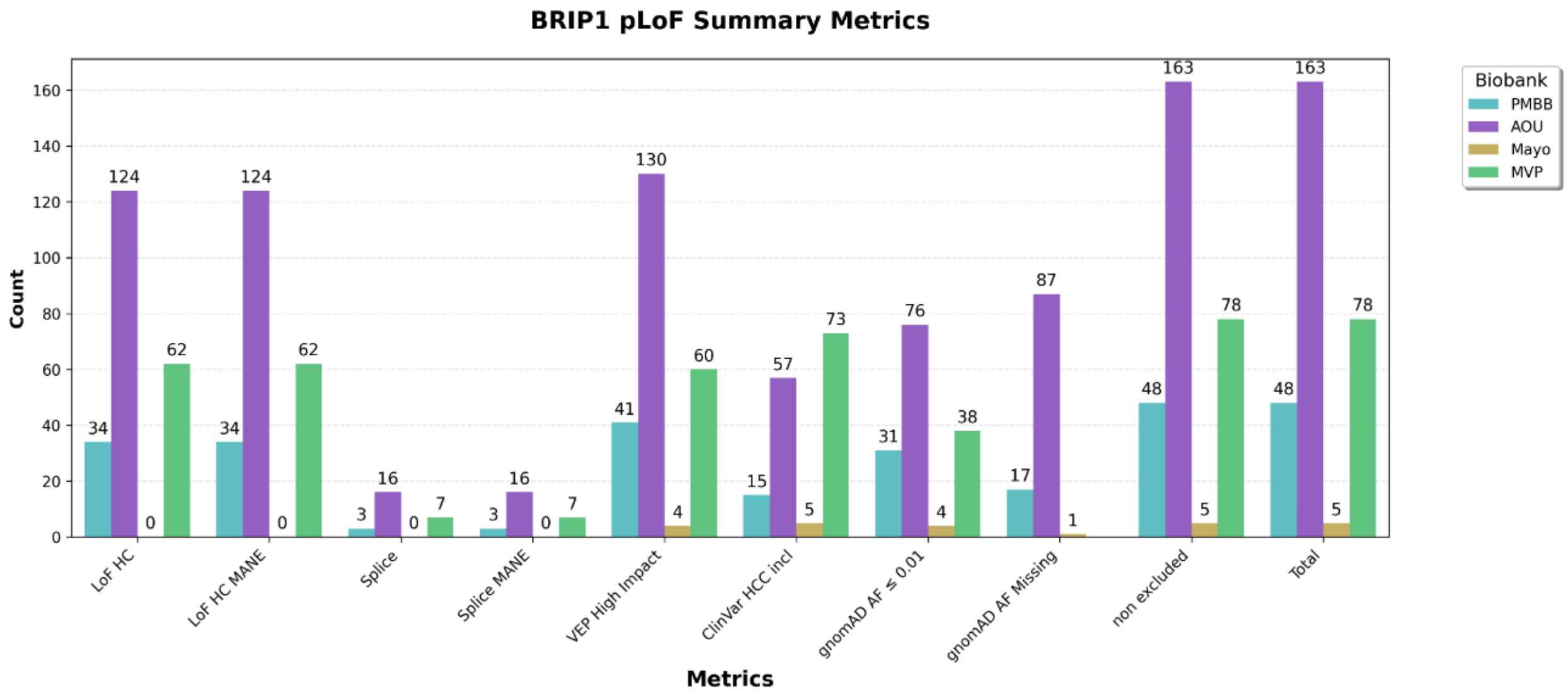
BRIP1 pLoF Summary Metrics
Count
Metrics
Biobank
PMBB
AOU
Mayo
MVP
LoF HC
LoF HC MANE
Splice
Splice MANE
VEP High Impact
ClinVar HCC incl
gnomAD AF ≤ 0.01
gnomAD AF Missing
non excluded
Total
34 124 0 62
34 124 0 62
3 16 0 7
3 16 0 7
41 130 4 60
15 57 5 73
31 76 4 38
17 87 1
48 163 5 78
48 163 5 78

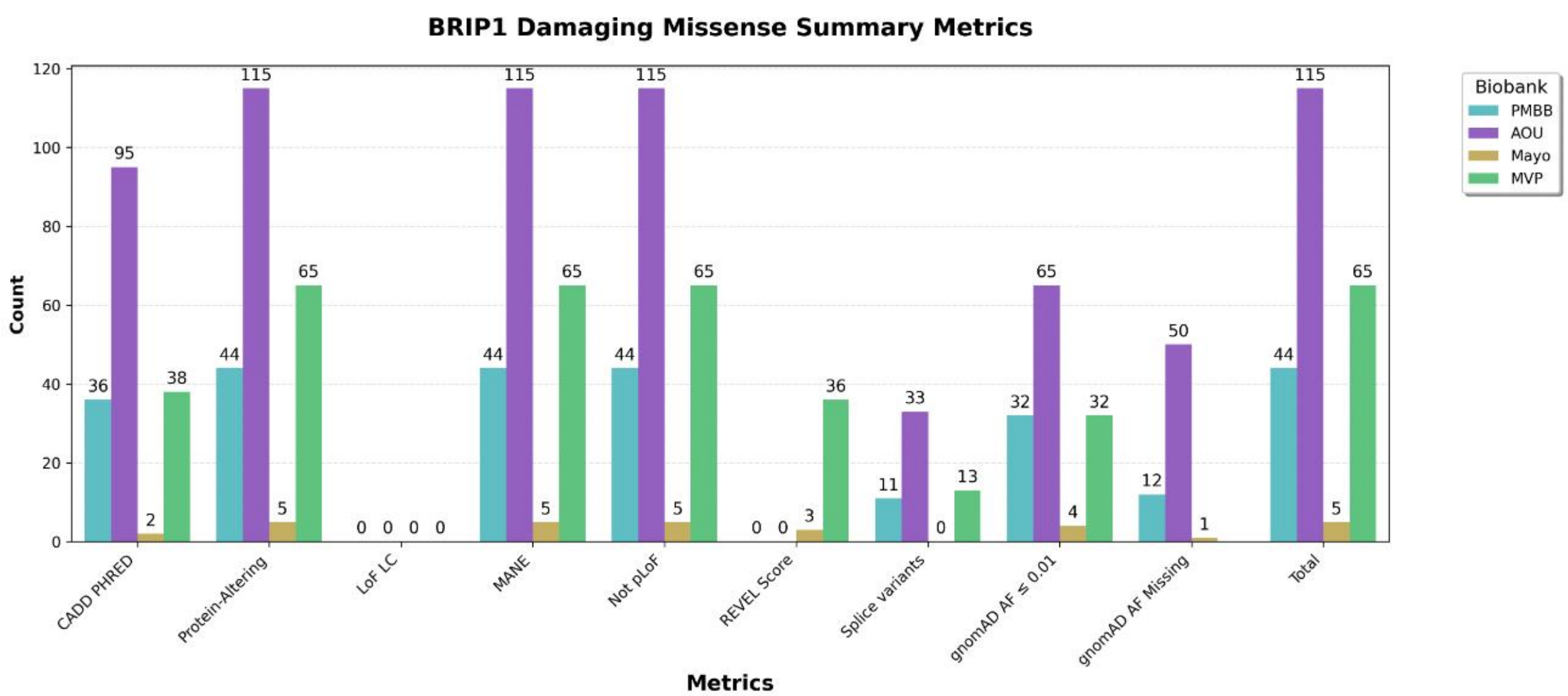
BRIP1 Damaging Missense Summary Metrics
Count
Metrics
Biobank
PMBB
AOU
Mayo
MVP
CADD PHRED
Protein-Altering
LoF LC
MANE
Not pLoF
REVEL Score
Splice variants
gnomAD AF ≤ 0.01
gnomAD AF Missing
Total
36 95 2 38
44 115 5 65
0 0 0 0
44 115 5 65
44 115 5 65
0 0 3 36
11 33 0 13
32 65 4 32
12 50 1
44 115 5 65

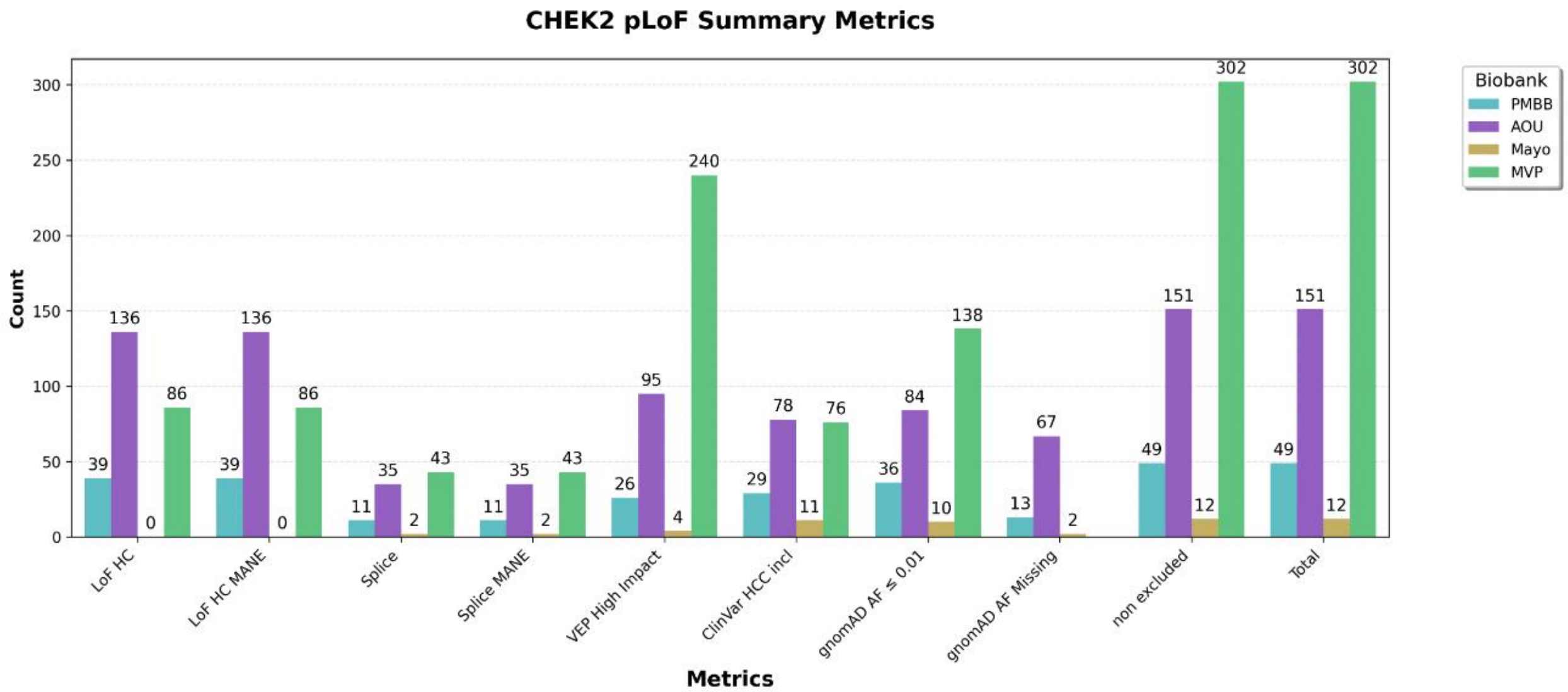
CHEK2 pLoF Summary Metrics
Biobank
PMBB
AOU
Mayo
MVP
Count
Metrics
LoF HC
LoF HC MANE
Splice
Splice MANE
VEP High Impact
ClinVar HCC incl
gnomAD AF ≤ 0.01
gnomAD AF Missing
non excluded
Total

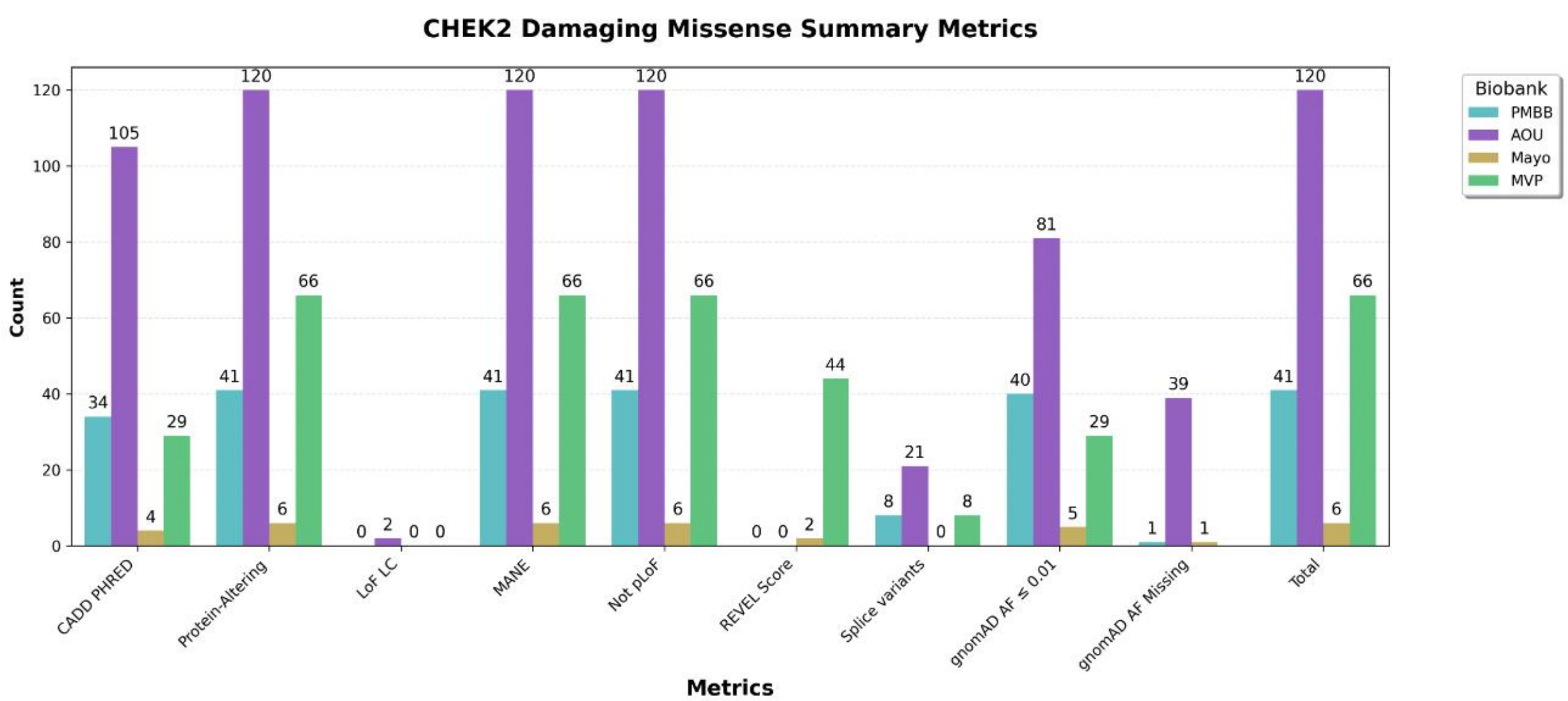
CHEK2 Damaging Missense Summary Metrics
Biobank
PMBB
AOU
Mayo
MVP
Count
Metrics
CADD PHRED
Protein-Altering
LoF LC
MANE
Not pLoF
REVEL Score
Splice variants
gnomAD AF ≤ 0.01
gnomAD AF Missing
Total

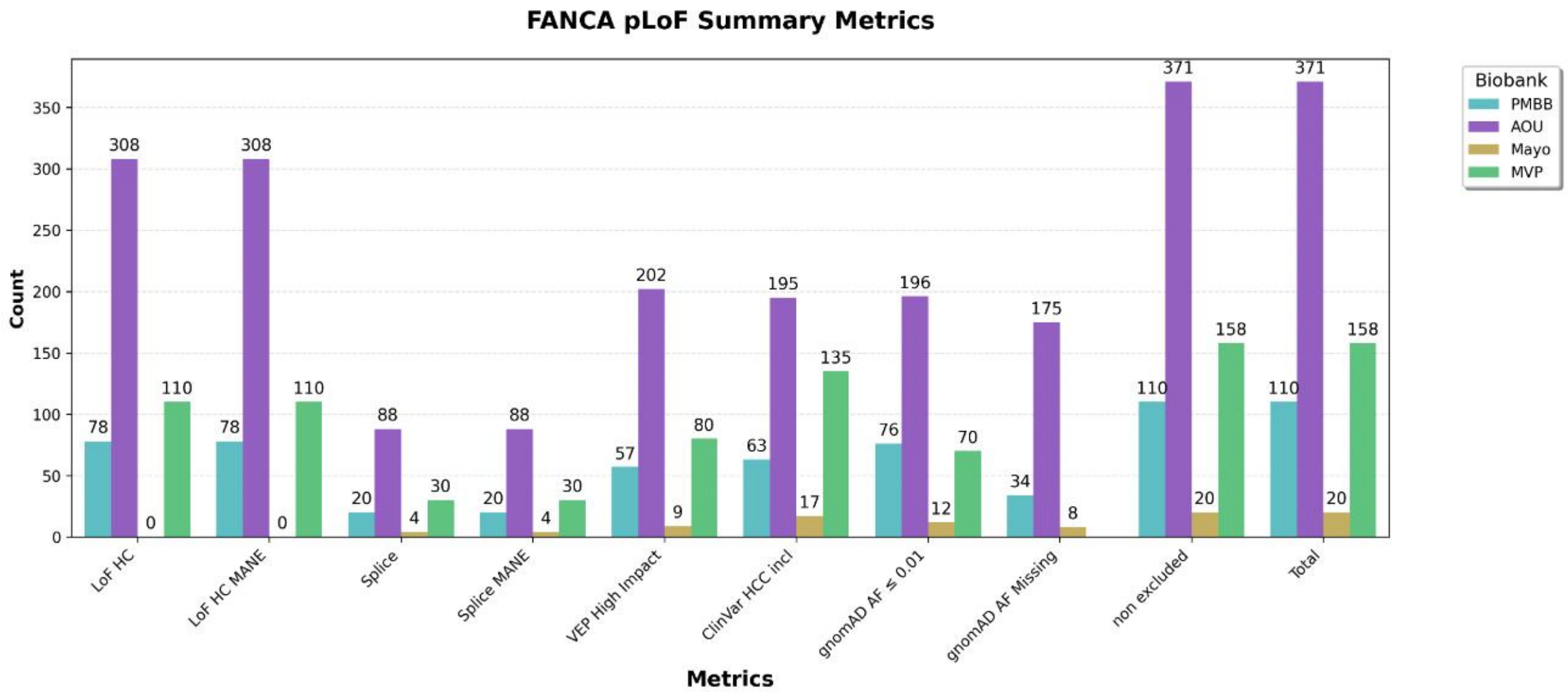
FANCA pLoF Summary Metrics
Count
Metrics
Biobank
PMBB
AOU
Mayo
MVP
LoF HC
LoF HC MANE
Splice
Splice MANE
VEP High Impact
ClinVar HCC incl
gnomAD AF ≤ 0.01
gnomAD AF Missing
non excluded
Total

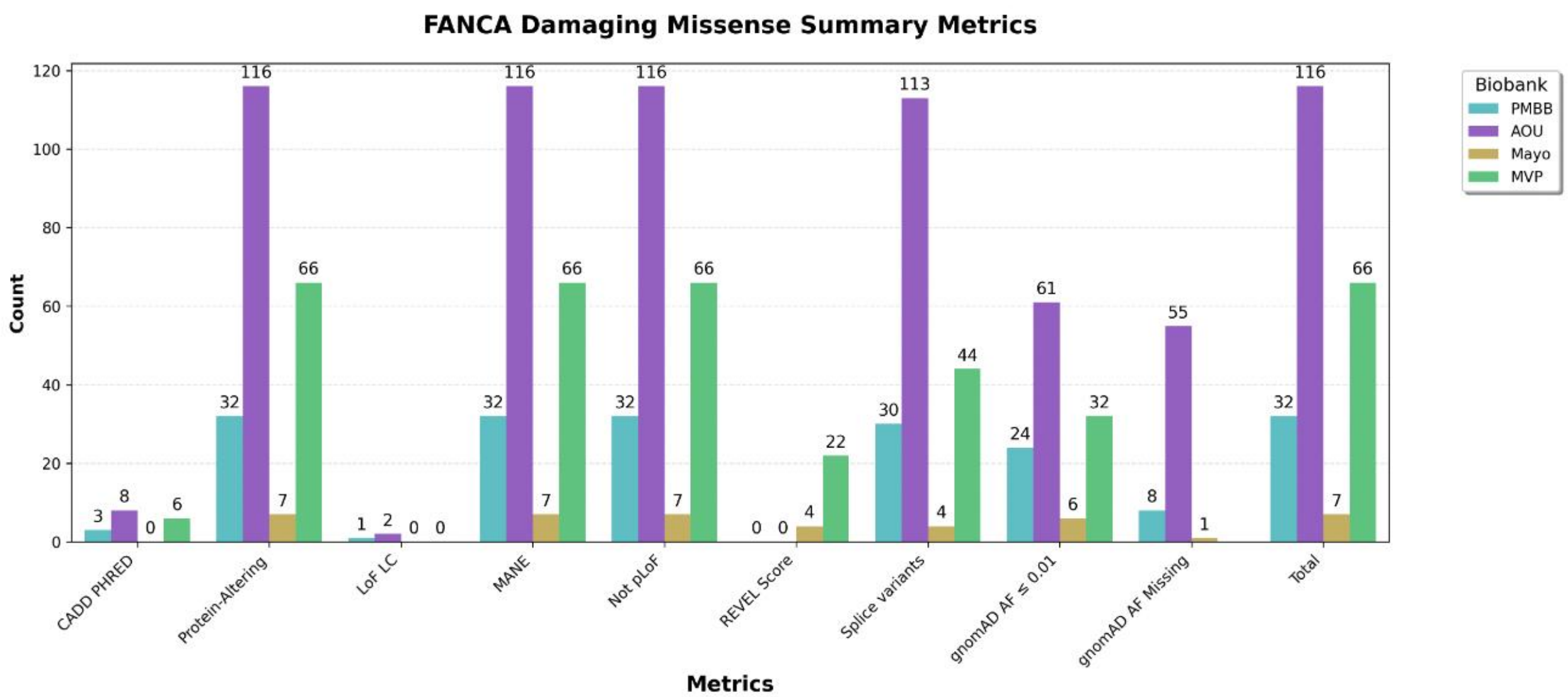
FANCA Damaging Missense Summary Metrics
Count
Metrics
Biobank
PMBB
AOU
Mayo
MVP
CADD PHRED
Protein-Altering
LoF LC
MANE
Not pLoF
REVEL Score
Splice variants
gnomAD AF ≤ 0.01
gnomAD AF Missing
Total

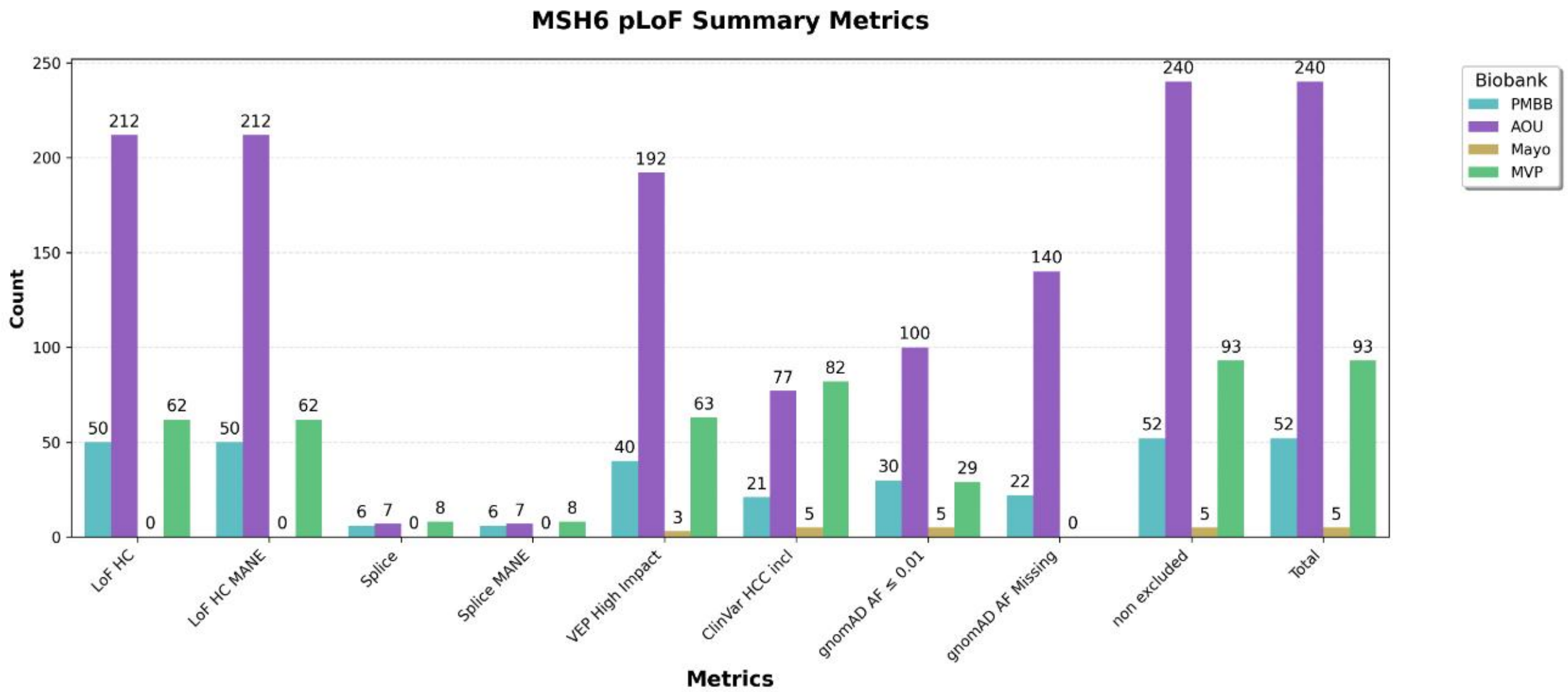
MSH6 pLoF Summary Metrics
Count
Metrics
Biobank
PMBB
AOU
Mayo
MVP
LoF HC
LoF HC MANE
Splice
Splice MANE
VEP High Impact
ClinVar HCC incl
gnomAD AF ≤ 0.01
gnomAD AF Missing
non excluded
Total

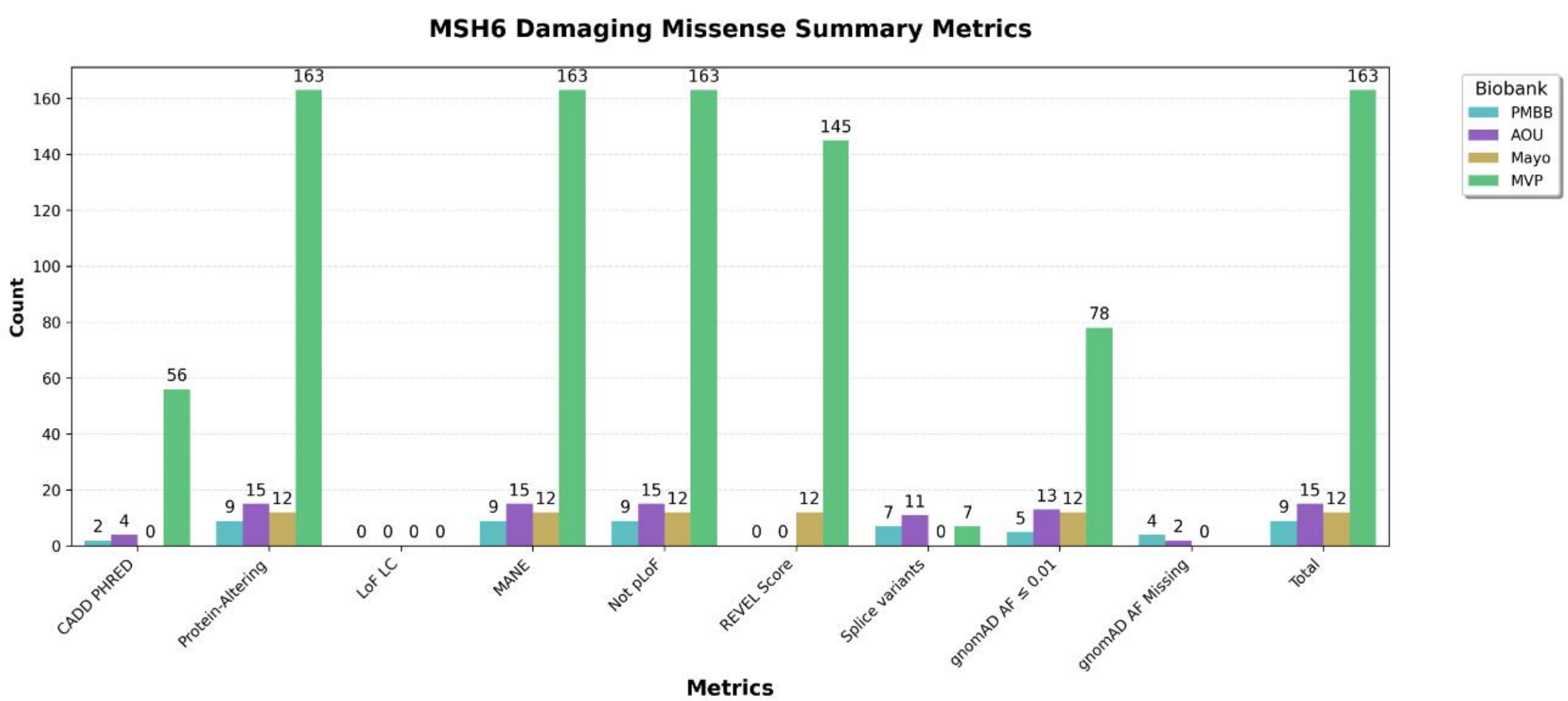
MSH6 Damaging Missense Summary Metrics
Count
Metrics
Biobank
PMBB
AOU
Mayo
MVP
CADD PHRED
Protein-Altering
LoF LC
MANE
Not pLoF
REVEL Score
Splice variants
gnomAD AF ≤ 0.01
gnomAD AF Missing
Total

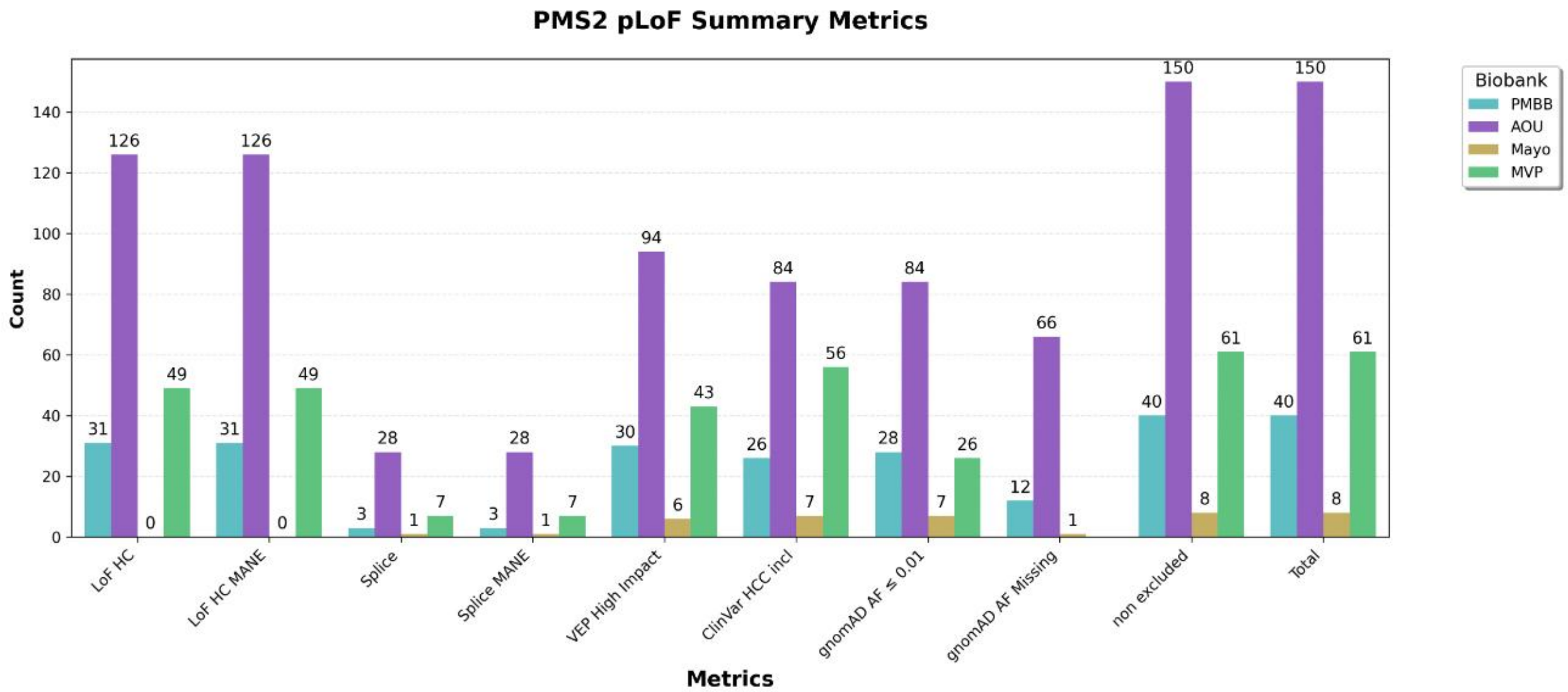
PMS2 pLoF Summary Metrics
Count
Metrics
Biobank
PMBB
AOU
Mayo
MVP
LoF HC
LoF HC MANE
Splice
Splice MANE
VEP High Impact
ClinVar HCC incl
gnomAD AF ≤ 0.01
gnomAD AF Missing
non excluded
Total
31
126
0
49
31
126
0
49
3
28
1
7
3
28
1
7
30
94
6
43
26
84
7
56
28
84
7
26
12
66
1
40
150
8
61
40
150
8
61

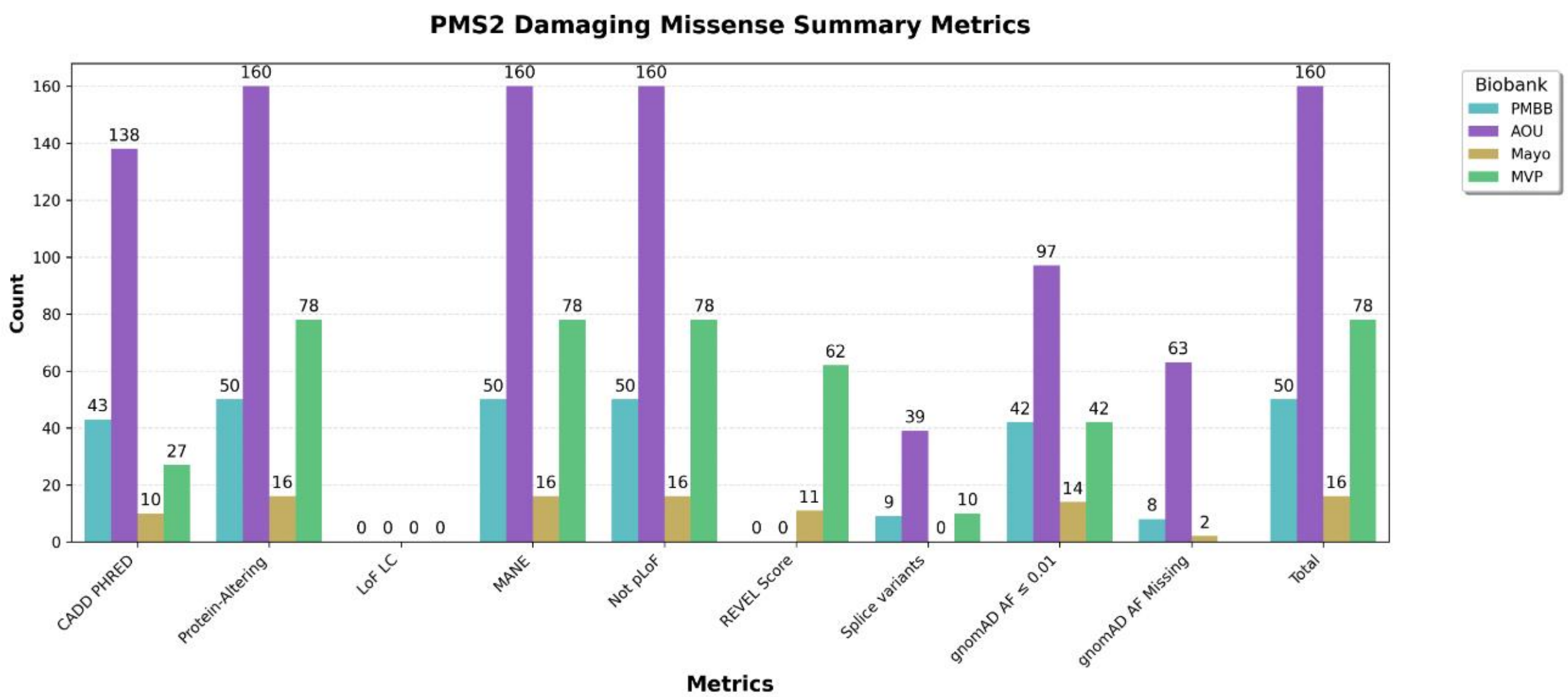
PMS2 Damaging Missense Summary Metrics
Count
Metrics
Biobank
PMBB
AOU
Mayo
MVP
CADD PHRED
Protein-Altering
LoF LC
MANE
Not pLoF
REVEL Score
Splice variants
gnomAD AF ≤ 0.01
gnomAD AF Missing
Total
43
138
10
27
50
160
16
78
0
0
0
0
50
160
16
78
50
160
16
78
0
0
11
62
9
39
0
10
42
97
14
42
8
63
2
50
160
16
78

Fig S2. Meta-analysis forest plot of *BRCA2* and *MSH6* gene burden associations with HCC risk in ALL populations after adjustment for liver disease comorbidities. Forest plots of odds ratios and 95% confidence intervals for gene burden associations with HCC risk across four biobanks (PMBB, MVP, AOU, Mayo Clinic) for: (A) MSH6 (B) BRCA2. Asterisks (*) indicate FDR < 0.05 (Benjamini-Hochberg). P-values from inverse variance weighted random-effects meta-analysis. Colored squares represent per-biobank odds ratios (OR); horizontal lines indicate 95% confidence intervals. The pink diamond represents the pooled random-effects meta-analytic estimate.

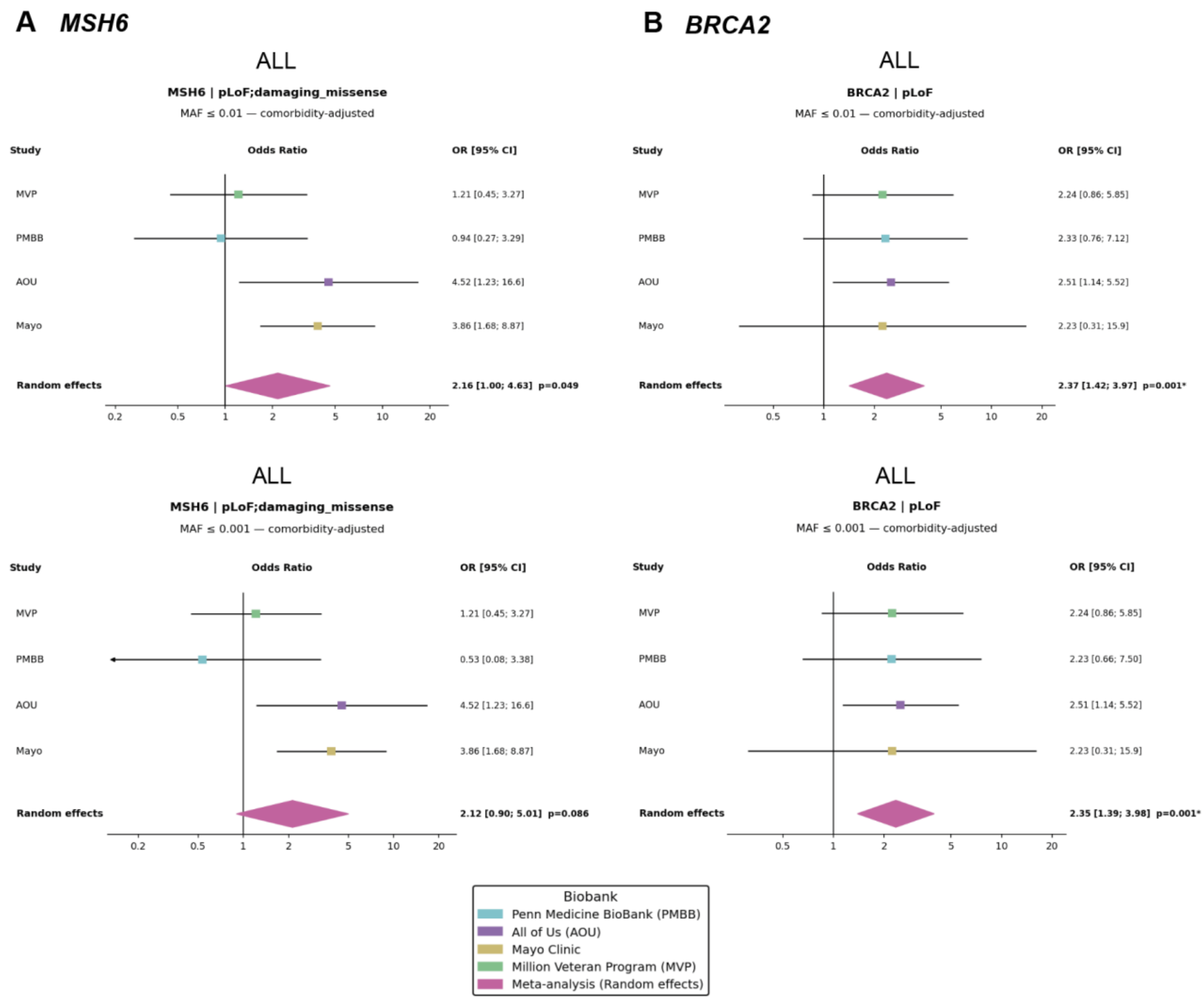

Fig S3. Meta-analysis forest plot of liver disease comorbidity enrichment in *BRCA2* and *MSH6* rare variant carriers among HCC cases in ALL populations. Forest plots of odds ratios and 95% confidence intervals comparing liver disease comorbidity prevalence in rare variant carriers versus non-carriers within HCC cases across four biobanks (PMBB, MVP, AOU, Mayo Clinic) for (A) *MSH6* and (B) *BRCA2*. Per-biobank estimates were derived using Fisher's exact test. Pooled odds ratios were estimated under a DerSimonian-Laird random-effects model.

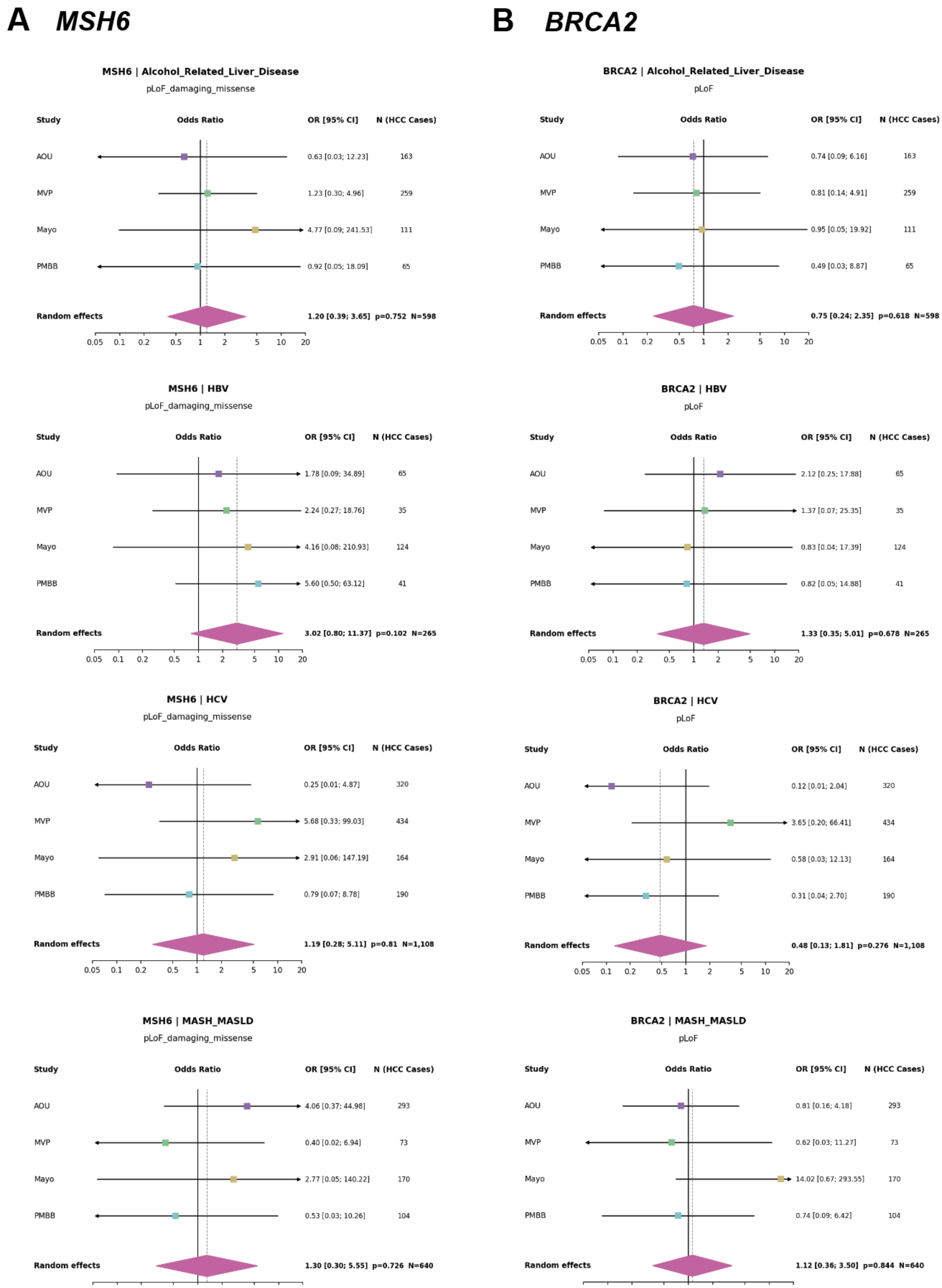
A MSH6
MSH6 | Alcohol_Related_Liver_Disease
pLoF_damaging_missense
Study
Odds Ratio
OR [95% CI]
N (HCC Cases)
AOU 0.63 [0.03; 12.23] 163
MVP 1.23 [0.30; 4.96] 259
Mayo 4.77 [0.09; 241.53] 111
PMBB 0.92 [0.05; 18.09] 65
Random effects 1.20 [0.39; 3.65] p=0.752 N=598
0.05 0.1 0.2 0.5 1 2 5 10 20
MSH6 | HBV
pLoF_damaging_missense
Study
Odds Ratio
OR [95% CI]
N (HCC Cases)
AOU 1.78 [0.09; 34.89] 65
MVP 2.24 [0.27; 18.76] 35
Mayo 4.16 [0.08; 210.93] 124
PMBB 5.60 [0.50; 63.12] 41
Random effects 3.02 [0.80; 11.37] p=0.102 N=265
0.05 0.1 0.2 0.5 1 2 5 10 20
MSH6 | HCV
pLoF_damaging_missense
Study
Odds Ratio
OR [95% CI]
N (HCC Cases)
AOU 0.25 [0.01; 4.87] 320
MVP 5.68 [0.33; 99.03] 434
Mayo 2.91 [0.06; 147.19] 164
PMBB 0.79 [0.07; 8.78] 190
Random effects 1.19 [0.28; 5.11] p=0.81 N=1,108
0.05 0.1 0.2 0.5 1 2 5 10 20
MSH6 | MASH_MASLD
pLoF_damaging_missense
Study
Odds Ratio
OR [95% CI]
N (HCC Cases)
AOU 4.06 [0.37; 44.98] 293
MVP 0.40 [0.02; 6.94] 73
Mayo 2.77 [0.05; 140.22] 170
PMBB 0.53 [0.03; 10.26] 104
Random effects 1.30 [0.30; 5.55] p=0.726 N=640
0.05 0.1 0.2 0.5 1 2 5 10 20
B BRCA2
BRCA2 | Alcohol_Related_Liver_Disease
pLoF
Study
Odds Ratio
OR [95% CI]
N (HCC Cases)
AOU 0.74 [0.09; 6.16] 163
MVP 0.81 [0.14; 4.91] 259
Mayo 0.95 [0.05; 19.92] 111
PMBB 0.49 [0.03; 8.87] 65
Random effects 0.75 [0.24; 2.35] p=0.618 N=598
0.05 0.1 0.2 0.5 1 2 5 10 20
BRCA2 | HBV
pLoF
Study
Odds Ratio
OR [95% CI]
N (HCC Cases)
AOU 2.12 [0.25; 17.88] 65
MVP 1.37 [0.07; 25.35] 35
Mayo 0.83 [0.04; 17.39] 124
PMBB 0.82 [0.05; 14.88] 41
Random effects 1.33 [0.35; 5.01] p=0.678 N=265
0.05 0.1 0.2 0.5 1 2 5 10 20
BRCA2 | HCV
pLoF
Study
Odds Ratio
OR [95% CI]
N (HCC Cases)
AOU 0.12 [0.01; 2.04] 320
MVP 3.65 [0.20; 66.41] 434
Mayo 0.58 [0.03; 12.13] 164
PMBB 0.31 [0.04; 2.70] 190
Random effects 0.48 [0.13; 1.81] p=0.276 N=1,108
0.05 0.1 0.2 0.5 1 2 5 10 20
BRCA2 | MASH_MASLD
pLoF
Study
Odds Ratio
OR [95% CI]
N (HCC Cases)
AOU 0.81 [0.16; 4.18] 293
MVP 0.62 [0.03; 11.27] 73
Mayo 14.02 [0.67; 293.55] 170
PMBB 0.74 [0.09; 6.42] 104
Random effects 1.12 [0.36; 3.50] p=0.844 N=640
0.05 0.1 0.2 0.5 1 2 5 10 20

Fig S4. Age at HCC diagnosis in *BRCA2* and *MSH6* rare variant carriers versus non-carriers among HCC cases in ALL populations. Mean age at hepatocellular carcinoma (HCC) diagnosis for carriers of rare variants in BRCA2 and MSH6 compared to non-carriers, stratified by variant category (pLoF, damaging missense, pLoF+damaging missense). Age at HCC diagnosis was defined as the interval from birth to the earliest recorded HCC diagnosis code within each biobank (PMBB, AOU, Mayo Clinic, MVP). Within each biobank, carriers were compared to non-carriers using Welch's two-sample t-test (two-sided, unequal variances). Bars represent the sample-size–weighted pooled mean age across biobanks; error bars indicate the pooled standard deviation. Δ denotes the meta-analytic mean age difference (carriers − non-carriers) estimated by the DerSimonian–Laird random-effects model. A negative Δ indicates earlier HCC diagnosis in carriers. Statistical significance was assessed using Benjamini–Hochberg false discovery rate (FDR) correction across all gene–category groups tested ($q < 0.05$). Carrier groups with fewer than 5 individuals ($n < 5$) are displayed as hatched bars with counts not shown to protect participant privacy.

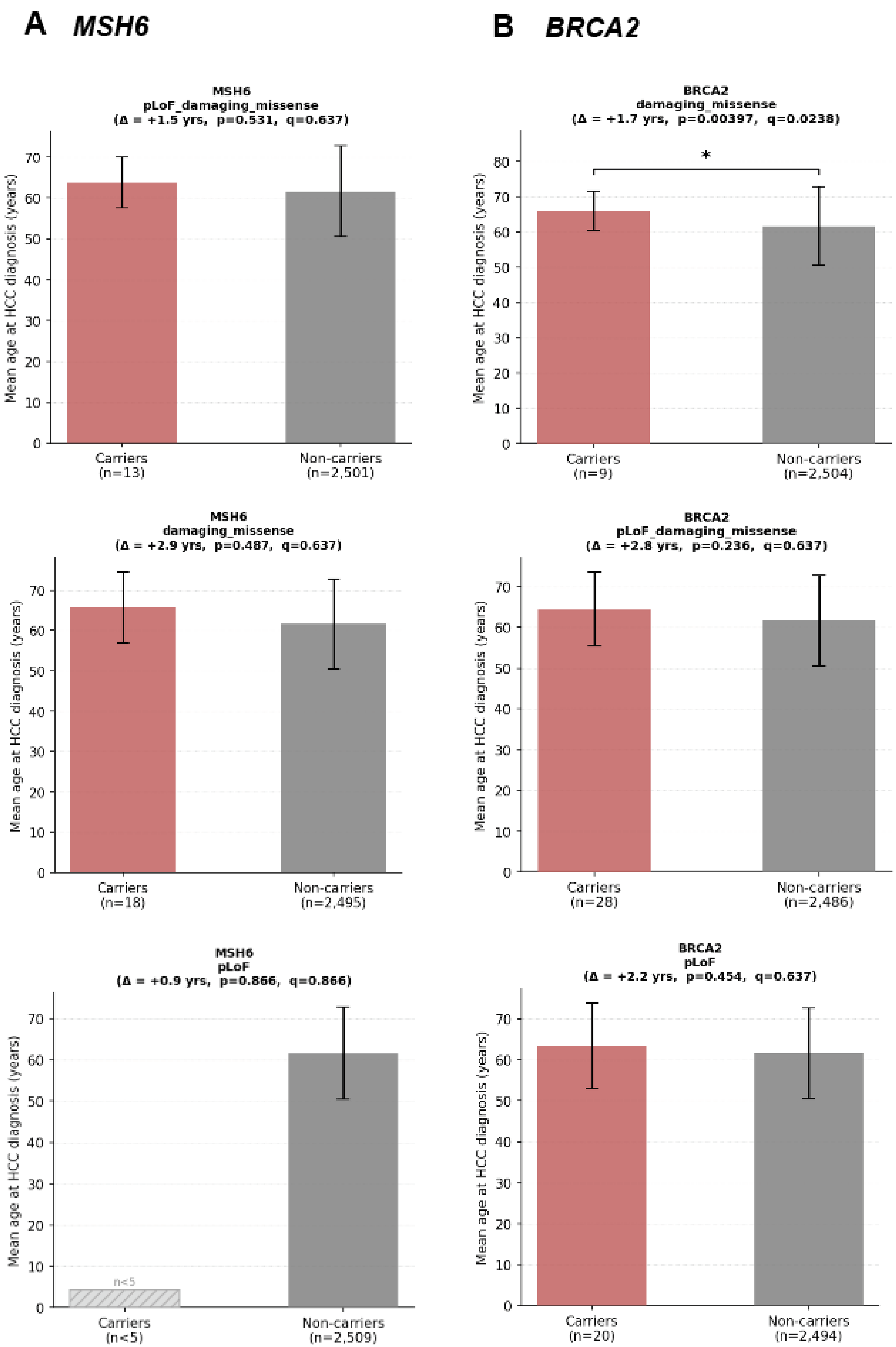
A MSH6
B BRCA2
MSH6
pLoF_damaging_missense
(Δ = +1.5 yrs, p=0.531, q=0.637)
Mean age at HCC diagnosis (years)
Carriers
(n=13)
Non-carriers
(n=2,501)
BRCA2
damaging_missense
(Δ = +1.7 yrs, p=0.00397, q=0.0238)
*
Carriers
(n=9)
Non-carriers
(n=2,504)
MSH6
damaging_missense
(Δ = +2.9 yrs, p=0.487, q=0.637)
Carriers
(n=18)
Non-carriers
(n=2,495)
BRCA2
pLoF_damaging_missense
(Δ = +2.8 yrs, p=0.236, q=0.637)
Carriers
(n=28)
Non-carriers
(n=2,486)
MSH6
pLoF
(Δ = +0.9 yrs, p=0.866, q=0.866)
n<5
Carriers
(n<5)
Non-carriers
(n=2,509)
BRCA2
pLoF
(Δ = +2.2 yrs, p=0.454, q=0.637)
Carriers
(n=20)
Non-carriers
(n=2,494)

Fig S5. Forest plots of significant sex-stratified meta-analysis of gene burden for HCC risk for *BRCA2* and *MSH6* in ALL populations. Forest plots of significant sex-stratified meta-analysis of gene burden for HCC Risk for (A) *MSH6* and (B) *BRCA2* pLoF+damaging missense burden associations shown at the indicated minor allele frequency (MAF) threshold. Squares indicate per-biobank odds ratio (OR) estimates with 95% confidence intervals from burden tests. Diamonds represent the pooled random-effects meta-analysis estimate (DerSimonian-Laird) for females (pink) and males (blue) separately. Horizontal lines extending beyond the plot range are shown as arrows. * FDR $q < 0.05$ (Benjamini-Hochberg correction across all gene-annotation-MAF-sex groups tested). Full results for all six genes, stratified by variant category (pLoF, damaging missense, pLoF+damaging missense) are available in Table S16.

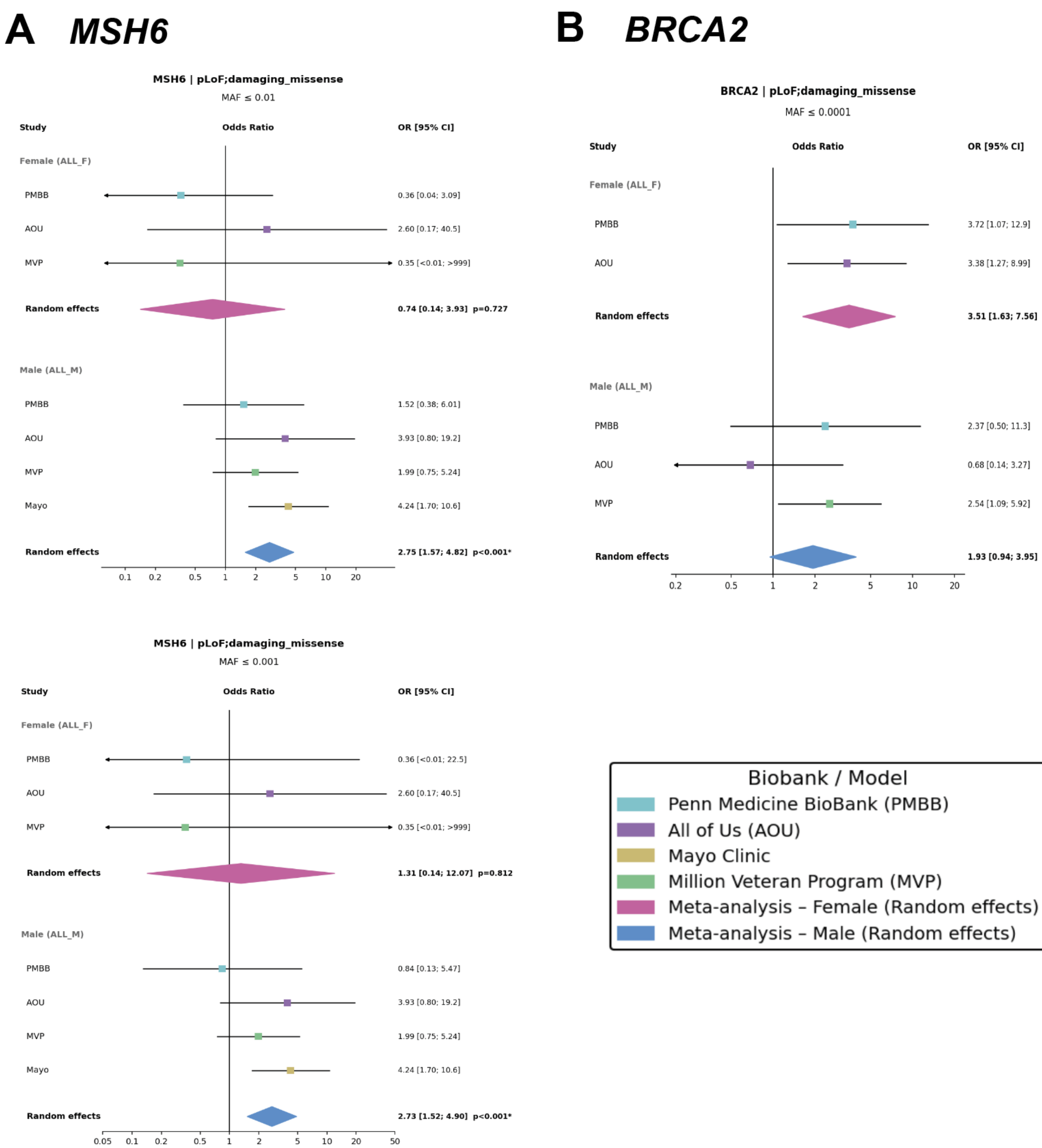
A MSH6
MSH6 | pLoF;damaging_missense
MAF ≤ 0.01
Study
Odds Ratio
OR [95% CI]
Female (ALL_F)
PMBB
0.36 [0.04; 3.09]
AOU
2.60 [0.17; 40.5]
MVP
0.35 [<0.01; >999]
Random effects
0.74 [0.14; 3.93] p=0.727
Male (ALL_M)
PMBB
1.52 [0.38; 6.01]
AOU
3.93 [0.80; 19.2]
MVP
1.99 [0.75; 5.24]
Mayo
4.24 [1.70; 10.6]
Random effects
2.75 [1.57; 4.82] p<0.001*
0.1 0.2 0.5 1 2 5 10 20
MSH6 | pLoF;damaging_missense
MAF ≤ 0.001
Study
Odds Ratio
OR [95% CI]
Female (ALL_F)
PMBB
0.36 [<0.01; 22.5]
AOU
2.60 [0.17; 40.5]
MVP
0.35 [<0.01; >999]
Random effects
1.31 [0.14; 12.07] p=0.812
Male (ALL_M)
PMBB
0.84 [0.13; 5.47]
AOU
3.93 [0.80; 19.2]
MVP
1.99 [0.75; 5.24]
Mayo
4.24 [1.70; 10.6]
Random effects
2.73 [1.52; 4.90] p<0.001*
0.05 0.1 0.2 0.5 1 2 5 10 20 50
B BRCA2
BRCA2 | pLoF;damaging_missense
MAF ≤ 0.0001
Study
Odds Ratio
OR [95% CI]
Female (ALL_F)
PMBB
3.72 [1.07; 12.9]
AOU
3.38 [1.27; 8.99]
Random effects
3.51 [1.63; 7.56] p=0.001*
Male (ALL_M)
PMBB
2.37 [0.50; 11.3]
AOU
0.68 [0.14; 3.27]
MVP
2.54 [1.09; 5.92]
Random effects
1.93 [0.94; 3.95] p=0.072
0.2 0.5 1 2 5 10 20
Biobank / Model
Penn Medicine BioBank (PMBB)
All of Us (AOU)
Mayo Clinic
Million Veteran Program (MVP)
Meta-analysis – Female (Random effects)
Meta-analysis – Male (Random effects)

Fig S6. Principal component analysis of genetic ancestry across contributing biobanks. Principal component analysis (PCA) plots for each contributing biobank: (A) Penn Medicine BioBank, (B) All of Us, (C) Mayo Clinic, and (D) Million Veteran Program. Individuals are colored by genetically inferred ancestry group (European [EUR], African [AFR], Admixed American [AMR], East Asian [EAS], South Asian [SAS], and Middle Eastern [MID]) and shaped by case-control status. Ancestry clusters were defined using cohort-specific principal components derived with reference panels (1000 Genomes Project, and additionally HGDP for All of Us). These plots illustrate the distribution of hepatocellular carcinoma cases and controls across ancestry groups within each cohort and reflect the underlying demographic composition of the biobanks.

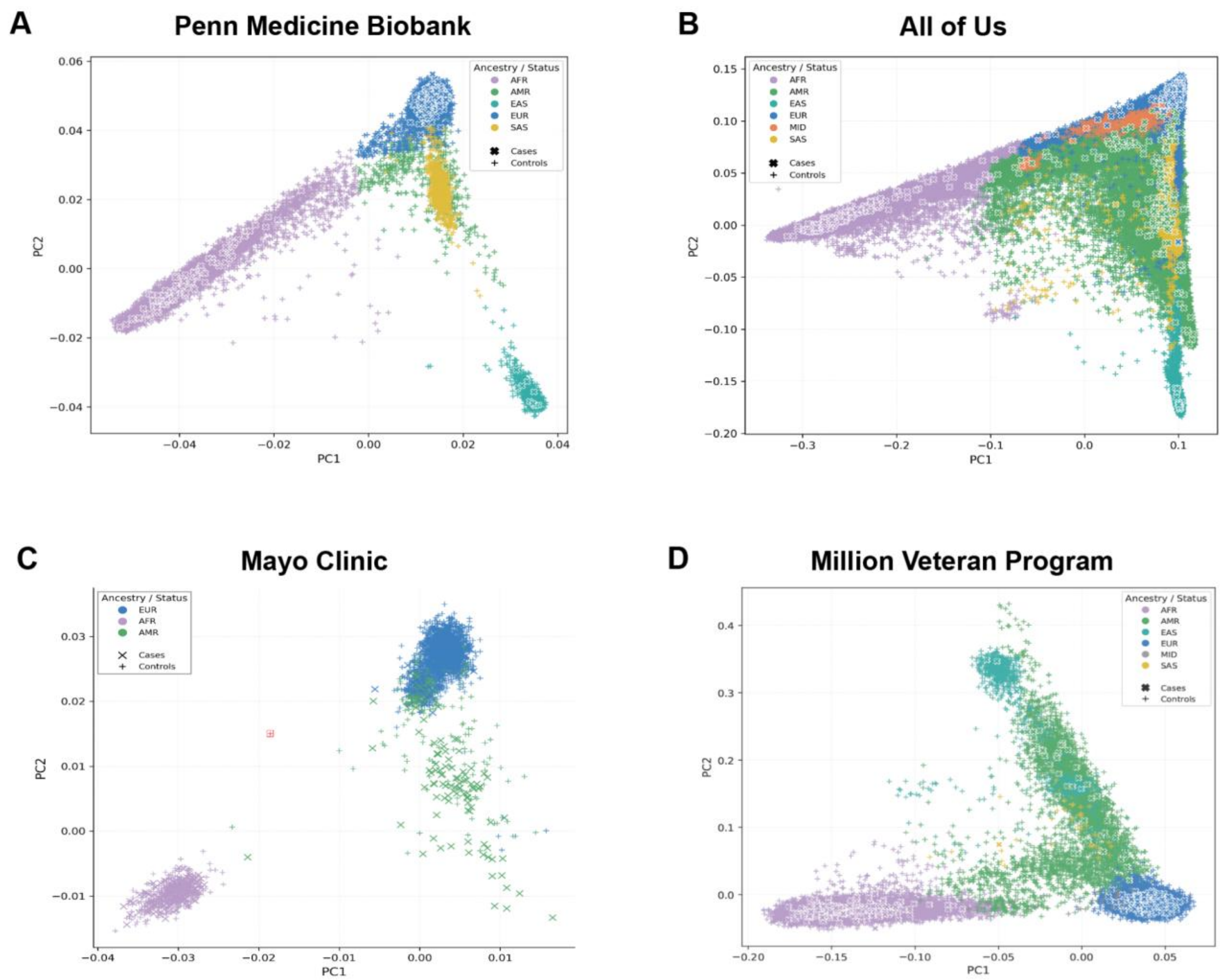

Fig S7. Summary of variant counts by biobank for ClinVar sensitivity analysis showing pLoF and damaging missense classifications. Bar plots showing the number of variants in *MSH6* and *PMS2* that meet inclusion criteria at each filtering step, stratified by biobank (PMBB, AOU, Mayo Clinic, MVP; colors shown in legend) and functional annotation category (pLoF and damaging missense). Left panels show predicted loss-of-function (pLoF) variant counts; right panels show damaging missense variant counts. The "Total" bar represents final variant counts after applying all filtering criteria described in Table S13.

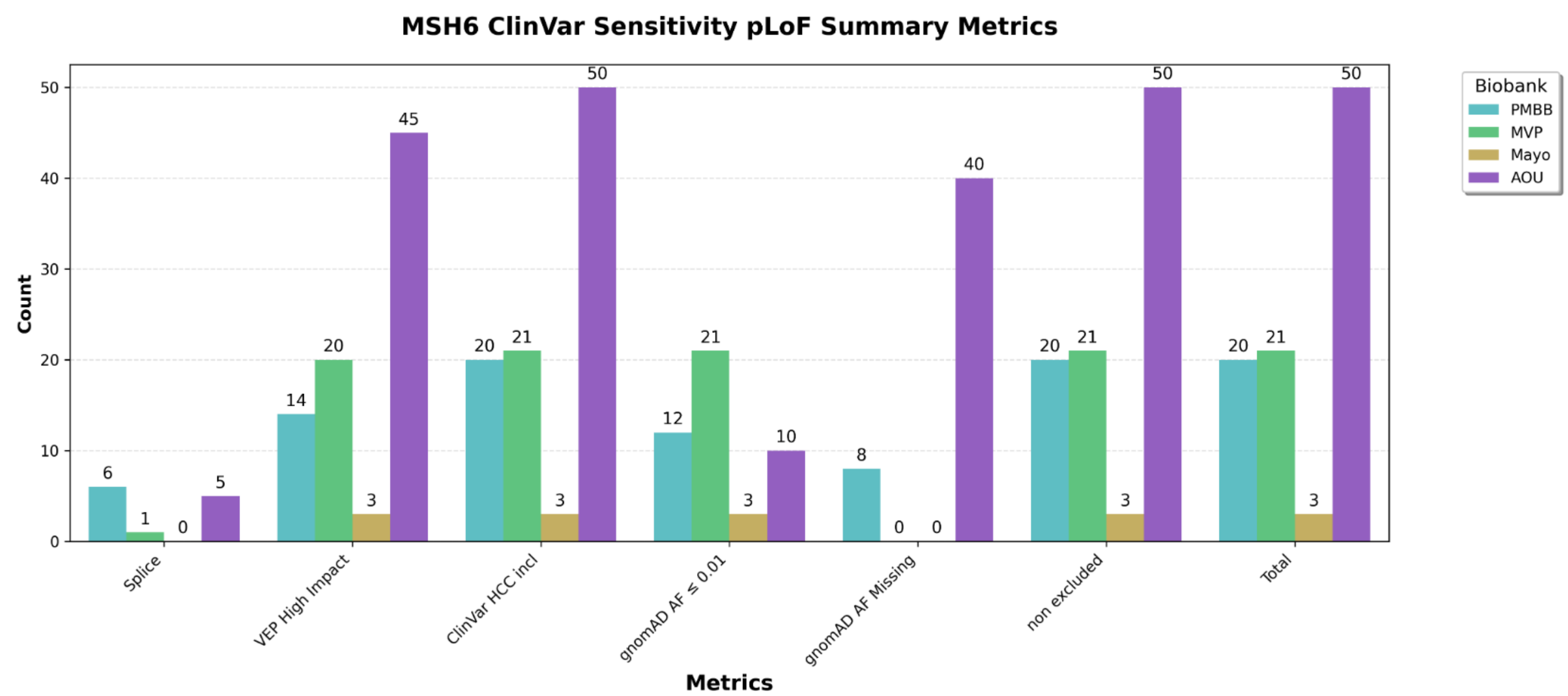

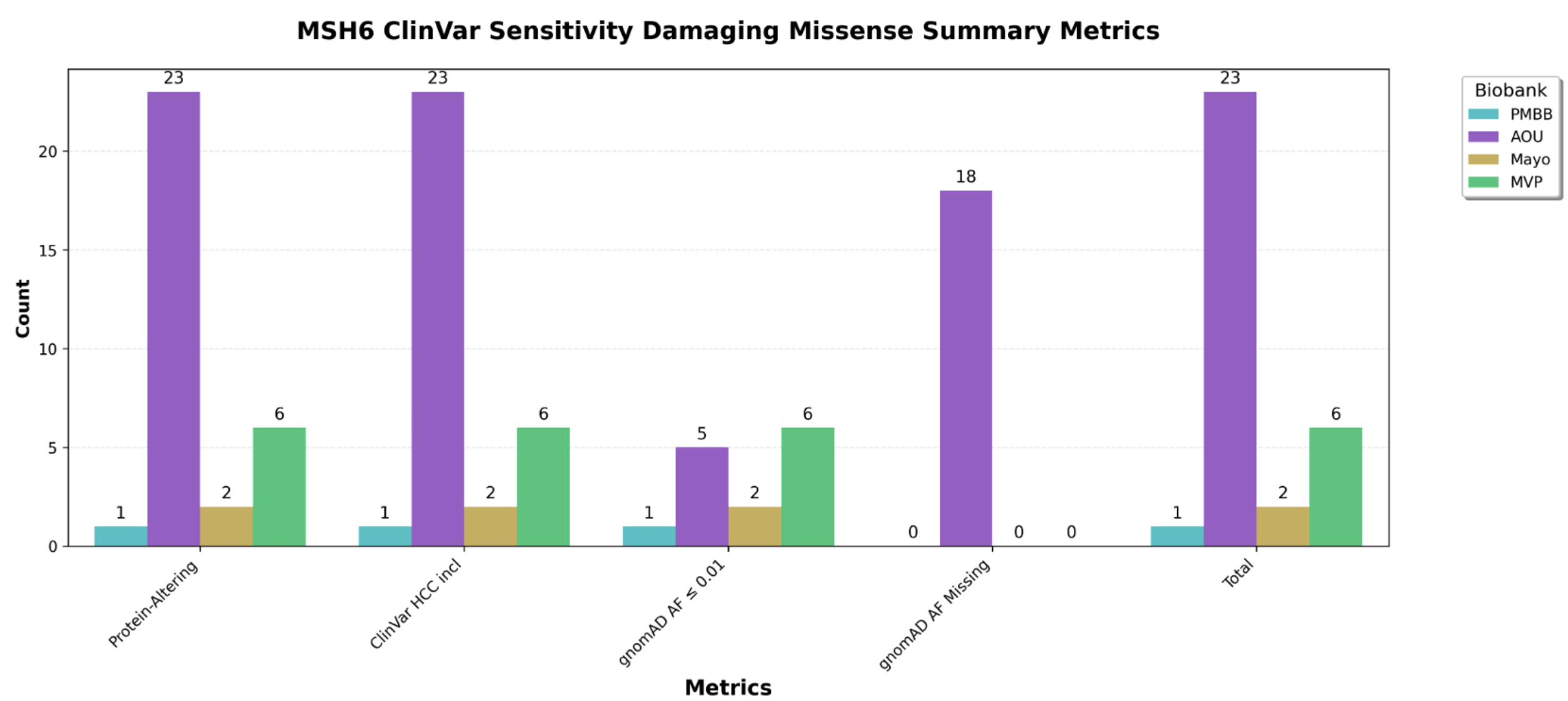

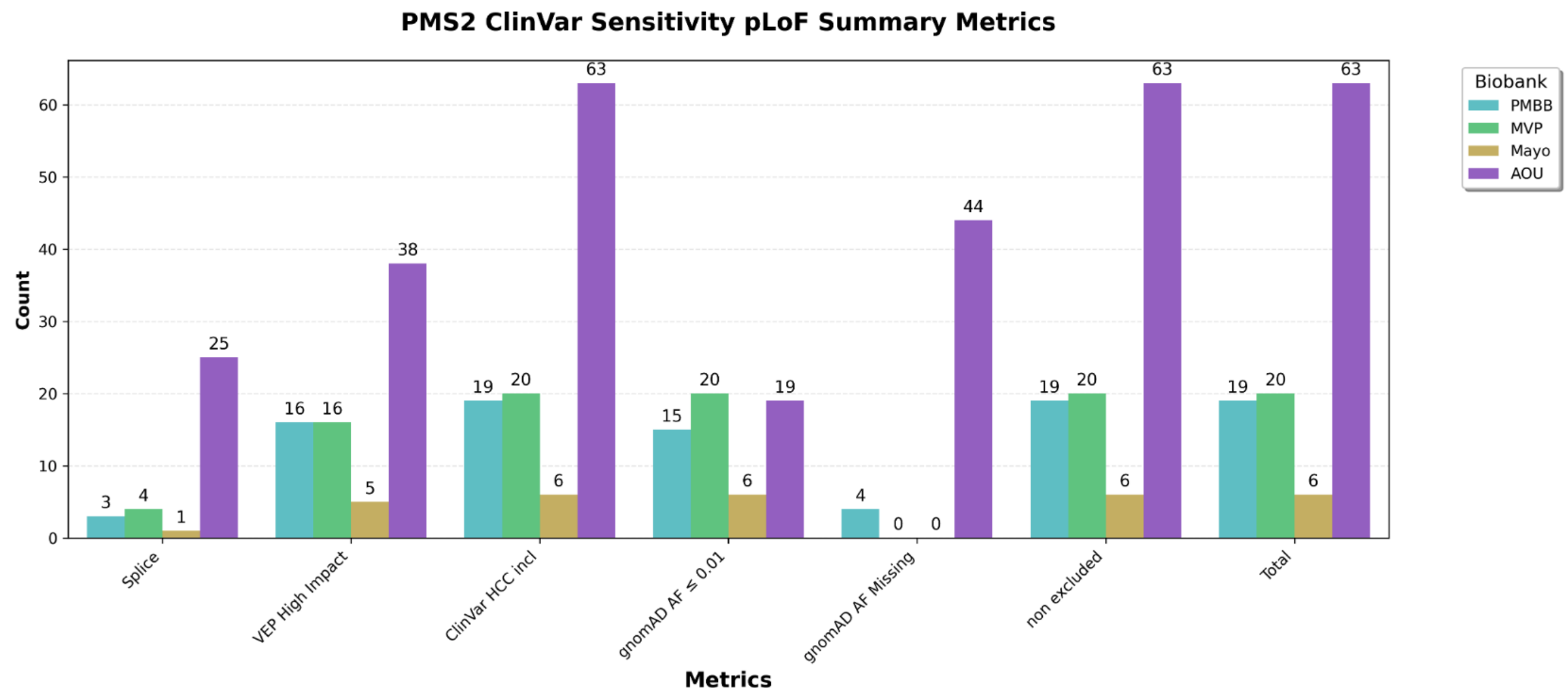
PMS2 ClinVar Sensitivity pLoF Summary Metrics
Count
Metrics
Splice
VEP High Impact
ClinVar HCC incl
gnomAD AF ≤ 0.01
gnomAD AF Missing
non excluded
Total
Biobank
PMBB
MVP
Mayo
AOU
3
4
1
25
16
16
5
38
19
20
6
63
15
20
6
19
4
0
0
44
19
20
6
63
19
20
6
63

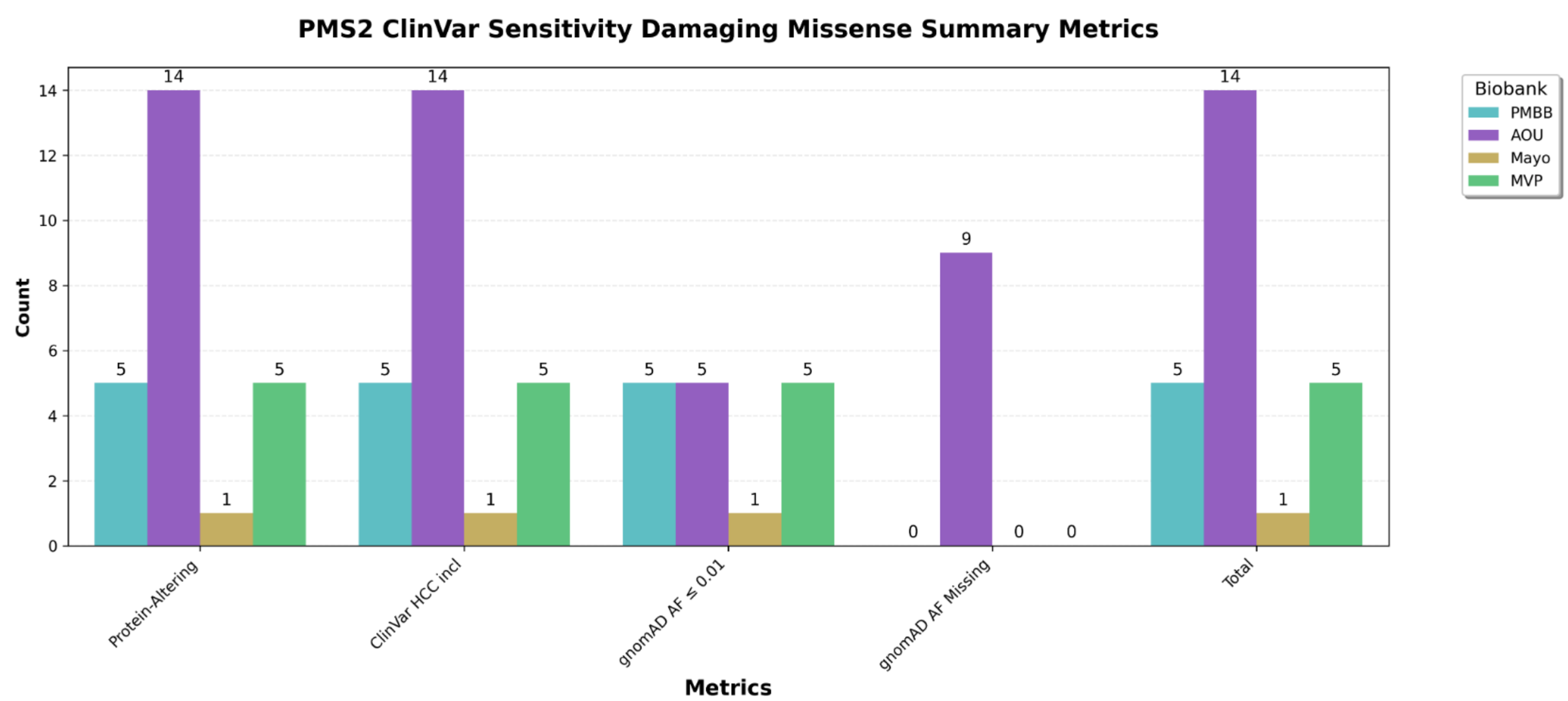
PMS2 ClinVar Sensitivity Damaging Missense Summary Metrics
Count
Metrics
Protein-Altering
ClinVar HCC incl
gnomAD AF ≤ 0.01
gnomAD AF Missing
Total
Biobank
PMBB
AOU
Mayo
MVP
5
14
1
5
5
14
1
5
5
5
1
5
0
9
0
0
5
14
1
5

Acknowledgements

Penn Medicine BioBank

PMBB Leadership Team

Daniel J. Rader, M.D.

Contribution: All authors contributed to securing funding, study design and oversight. All authors reviewed the final version of the manuscript.

Patient Recruitment and Regulatory Oversight

JoEllen Weaver, Nawar Naseer, Ph.D., M.P.H., Giorgio Sirugo, M.D., P.h.D., Afiya Poindexter, Jenna Dever, Aidan Harvey, Sydney Linn, Naman Srivastava

Contributions: JW manages patient recruitment and regulatory oversight of study. NN manages participant engagement, assists with regulatory oversight, and researcher access. GS assists with researcher access. AP, JD, AH, SL, and NS perform recruitment and enrollment of study participants.

Lab Operations

JoEllen Weaver, Meghan Livingstone, Fred Vadivieso, Stephanie DerOhannessian, Teo Tran, Julia Stephanowski, Salma Santos, Ned Haubein, P.h.D., Joseph Dunn

Contribution: JW, ML, FV, SD conduct oversight of lab operations. ML, FV, AK, SD, TT, JS, SS perform sample processing. NH, JD are responsible for sample tracking and the laboratory information management system.

Clinical Informatics

Anurag Verma, Ph.D., Colleen Morse Kripke, M.S. DPT, MSA, Marjorie Risman, M.S., Renae Judy, B.S., Colin Wollack, M.S.

Contribution: All authors contributed to the development and validation of clinical phenotypes used to identify study subjects and (when applicable) controls.

Genome Informatics

Anurag Verma Ph.D., Shefali S. Verma, Ph.D., Scott Damrauer, M.D., Yuki Bradford, M.S., Scott Dudek, M.S., Theodore Drivas, M.D., Ph.D., Zachary Rodriguez, Ph.D,

Contribution: AV, SSV, and SD are responsible for the analysis, design, and infrastructure needed to quality control genotype and exome data. YB performs

the analysis. TD and AV provides variant and gene annotations and their functional interpretation of variants.

VA Million Veteran Program |Core Acknowledgements for Publications

MVP Program Office
- Sumitra Muralidhar, Ph.D., Program Director

US Department of Veterans Affairs, 810 Vermont Avenue NW, Washington, DC 20420
- Jennifer Moser, Ph.D., Associate Director, Scientific Programs

US Department of Veterans Affairs, 810 Vermont Avenue NW, Washington, DC 20420
- Jennifer E. Deen, B.S., Associate Director, Cohort & Public Relations

US Department of Veterans Affairs, 810 Vermont Avenue NW, Washington, DC 20420

MVP Steering Committee
- Co-Chair: Philip S. Tsao, Ph.D.
VA Palo Alto Health Care System, 3801 Miranda Avenue, Palo Alto, CA 94304
- Co-Chair: Sumitra Muralidhar, Ph.D.
US Department of Veterans Affairs, 810 Vermont Avenue NW, Washington, DC 20420
- J. Michael Gaziano, M.D., M.P.H.
VA Boston Healthcare System, 150 S. Huntington Avenue, Boston, MA 02130
- Adriana Hung, M.D., M.P.H.,

VA Tennessee Valley Healthcare System, 1310 24th Avenue, South Nashville, TN 37212
- Dave Oslin, M.D.
Philadelphia VA Medical Center, 3900 Woodland Avenue, Philadelphia, PA 19104
- Deepak Voora, M.D.
Durham VA Medical Center, 508 Fulton Street, Durham, NC 27705

MVP Co-Principal Investigators
- J. Michael Gaziano, M.D., M.P.H.

VA Boston Healthcare System, 150 S. Huntington Avenue, Boston, MA 02130
- Philip S. Tsao, Ph.D.

VA Palo Alto Health Care System, 3801 Miranda Avenue, Palo Alto, CA 94304

MVP Core Operations

- Jessica V. Brewer, M.P.H., Director, MVP Cohort Operations

VA Boston Healthcare System, 150 S. Huntington Avenue, Boston, MA 02130
- Mary T. Brophy M.D., M.P.H., Director, VA Central Biorepository

VA Boston Healthcare System, 150 S. Huntington Avenue, Boston, MA 02130
- Kelly Cho, M.P.H, Ph.D., Director, MVP Phenomics

VA Boston Healthcare System, 150 S. Huntington Avenue, Boston, MA 02130
- Lori Churby, B.S., Director, MVP Regulatory Affairs

VA Palo Alto Health Care System, 3801 Miranda Avenue, Palo Alto, CA 94304
- Jacob T. Kean, Ph.D., Acting Director, VA Informatics and Computing Infrastructure (VINCI)

MVP Core Acknowledgements for Publications_October 2025
VA Salt Lake City Health Care System, 500 Foothill Drive, Salt Lake City, UT 84148
- Saiju Pyarajan Ph.D., Director, Data and Computational Sciences

VA Boston Healthcare System, 150 S. Huntington Avenue, Boston, MA 02130
- Robert Ringer, Pharm.D., Director, VA Albuquerque Central Biorepository

New Mexico VA Health Care System, 1501 San Pedro Drive SE, Albuquerque, NM 87108
- Luis E. Selva, Ph.D., Director, MVP Biorepository Coordination

VA Boston Healthcare System, 150 S. Huntington Avenue, Boston, MA 02130
- Shahpoor (Alex) Shayan, M.S., Director, MVP PRE Informatics

VA Boston Healthcare System, 150 S. Huntington Avenue, Boston, MA 02130
- Brady Stephens, M.S., Principal Investigator, MVP Information Center

Canandaigua VA Medical Center, 400 Fort Hill Avenue, Canandaigua, NY 14424
- Stacey B. Whitbourne, Ph.D., Director, MVP Cohort Development and Management

VA Boston Healthcare System, 150 S. Huntington Avenue, Boston, MA 02130

Supplementary references

1. Marrero, J. A. et al. Diagnosis, Staging, and Management of Hepatocellular Carcinoma: 2018 Practice Guidance by the American Association for the Study of Liver Diseases. Hepatology 68, 723 (2018).
2. Verma, A. et al. The Penn Medicine BioBank: Towards a Genomics-Enabled Learning Healthcare System to Accelerate Precision Medicine in a Diverse Population. Journal of Personalized Medicine 12, 1974 (2022).
3. Li, H. & Durbin, R. Fast and accurate short read alignment with Burrows-Wheeler transform. Bioinformatics 25, 1754–1760 (2009).
4. Krasheninina, O. et al. Open-source mapping and variant calling for large-scale NGS data from original base-quality scores. 2020.12.15.356360 Preprint at https://doi.org/10.1101/2020.12.15.356360 (2020).
5. Lin, M. F. et al. GLnexus: joint variant calling for large cohort sequencing. 343970 Preprint at https://doi.org/10.1101/343970 (2018).
6. Chang, C. C. et al. Second-generation PLINK: rising to the challenge of larger and richer datasets. Gigascience 4, 7 (2015).
7. Bick, A. G. et al. Genomic data in the All of Us Research Program. Nature 627, 340–346 (2024).
8. nep63. nep63/AllofUs. (2025).
9. Jawaid, T. et al. Characterization of the Cystic Phenotype Associated with Monoallelic ALG8 and ALG9 Pathogenic Variants. J Am Soc Nephrol 36, 1056–1071 (2025).
10. Verma-Lab. HCC_Analysis_Plan/variant_group_summary.py at main · Verma-

Lab/HCC_Analysis_Plan. GitHub https://github.com/Verma-Lab/HCC_Analysis_Plan/blob/main/variant_group_summary.py.
