# Supplementary material for: Multi-ancestry sequencing analysis in 293,141 participants identifies predisposition DNA-repair genes associated with HCC risk
Source: JHEP Rep. 2026 Aug 29;8(12):102019. doi: 10.1016/j.jhepr.2026.102019 (PMC13755941; doi:10.1016/j.jhepr.2026.102019)
Supplement: Supplementary file 4 [file mmc2.docx]

**JHEP Reports**

**CTAT methods**

Tables for a “Complete, Transparent, Accurate and Timely account” (CTAT) are now mandatory for all revised submissions. The aim is to enhance the reproducibility of methods.

- Only include the parts relevant to your study
- Refer to the CTAT in the main text as ‘Supplementary CTAT Table’
- Do not add subheadings
- Add as many rows as needed to include all information
- Only include one item per row

**If the CTAT form is not relevant to your study, please outline the reasons why:**

1.1 **Antibodies**

| **Name** | **Citation** | **Supplier** | **Cat no.** | **Clone no.** |
| --- | --- | --- | --- | --- |
|  |  |  |  |  |

1.2 **Cell lines**

| **Name** | **Citation** | **Supplier** | **Cat no.** | **Passage no.** | **Authentication test method** |
| --- | --- | --- | --- | --- | --- |
|  |  |  |  |  |  |

1.3 **Organisms**

| **Name** | **Citation** | **Supplier** | **Strain** | **Sex** | **Age** | **Overall n number** |
| --- | --- | --- | --- | --- | --- | --- |
|  |  |  |  |  |  |  |

1.4 **Sequence based reagents**

| **Name** | **Sequence** | **Supplier** |
| --- | --- | --- |
|  |  |  |

1.5 **Biological samples**

| **Description** | **Source** | **Identifier** |
| --- | --- | --- |
|  |  |  |

1.6 **Deposited data**

| **Name of repository** | **Identifier** | **Link** |
| --- | --- | --- |
| Github | hcc-rare-variant-analysis | https://github.com/Verma-Lab/hcc-rare-variant-analysis |

1.7 **Software**

| **Software name** | **Manufacturer** | **Version** |
| --- | --- | --- |
| R | R Development Core Team | 4.2.0 |
| Python | Python Software Foundation | 3.8.0 |
| SAIGE-GENE+ | Lee Lab/ University of Michigan | 1.4.4 |
| metafor | Wolfgang Viechtbauer | 4.8-0 |
| GATK | Broad Institute | 4.1.0.0 |
| BWA-MEM | Heng Li | 0.7.15 |
| Ensembl VEP | EMBL-EBI | 113.0 (MVP 110) |

1.8 **Other (*e.g*. drugs, proteins, vectors etc.)**

1.9 **Please provide the details of the corresponding methods author for the manuscript:**

Anurag Verma, PhD

3800 Hamilton Walk

A202 Richards Laboratory

Philadelphia, PA 19104

anurag.verma at pennmedicine.upenn.edu

**2.0	Please confirm for randomised controlled trials all versions of the clinical protocol are included in the submission. These will be published online as supplementary information.**
